# Comprehensive functional network analysis and screening of deleterious pathogenic variants in non-syndromic hearing loss causative genes

**DOI:** 10.1042/BSR20211865

**Published:** 2021-10-29

**Authors:** Manisha Ray, Saurav Sarkar, Mukund Namdev Sable

**Affiliations:** 1 Department of Pathology and Lab Medicine, All India Institute of Medical Sciences, Bhubaneswar 751019, Odisha, India; 2 Department of ENT, All India Institute of Medical Sciences, Bhubaneswar 751019, Odisha, India

**Keywords:** Functional network, Genetics, In silico, Non-syndromic hearing loss, Single nucleotide polymorphism

## Abstract

Hearing loss (HL) is a significant public health problem and causes the most frequent congenital disability in developed societies. The genetic analysis of non-syndromic hearing loss (NSHL) may be considered as a complement to the existent plethora of diagnostic modalities available. The present study focuses on exploring more target genes with respective non-synonymous single nucleotide polymorphisms (nsSNPs) involved in the development of NSHL. The functional network analysis and variant study have successfully been carried out from the gene pool retrieved from reported research articles of the last decade. The analyses have been done through STRING. According to predicted biological processes, various variant analysis tools have successfully classified the NSHL causative genes and identified the deleterious nsSNPs, respectively. Among the predicted pathogenic nsSNPs with rsIDs rs80356586 (I515T), rs80356596 (L1011P), rs80356606 (P1987R) in *OTOF* have been reported in NSHL earlier. The rs121909642 (P722S), rs267606805 (P722H) in *FGFR1*, rs121918506 (E565A) and rs121918509 (A628T, A629T) in *FGFR2* have not been reported in NSHL yet, which should be clinically experimented in NSHL. This also indicates this variant’s novelty as its association in NSHL. The findings and the analyzed data have delivered some vibrant genetic pathogenesis of NSHL. These data might be used in the diagnostic and prognostic purposes in non-syndromic congenitally deaf children.

## Introduction

Hearing impairment is the highest age-standardized disability globally [1]. It affects nearly 1 in every 1000 livelihoods worldwide [2]. Hearing impairment affects speech development and language acquirement and hinders children’s education [3]. The causes of hearing loss (HL) can broadly be classified as conductive, sensorineural, and mixed HL. HL, which is predominantly due to genetic etiology, is usually present in an early life without any additional clinical phenotypes.

Seventy percent of neonates with HL are presumed to have inherited HL, classified as non-syndromic hearing loss (NSHL). They are not associated with other distinguishing physical findings [1]. NSHL generally follows simple Mendelian inheritance with a 75–80% transmission rate as autosomal recessive, 20% as autosomal dominant, 25% as X-linked and remaining 1% as mitochondrial mutation [1,2,4]. Fifty percent of congenital sensorineural HL is hereditary, caused by genetic mutations in a single gene or combination of multiple genes [3].

The explosions of genetic information and advancement in technology have radically improved the deep understanding of inherited diseases. In the case of NSHL, genetic correlations are a significant challenge due to wide clinical and genetic heterogeneity. Due to the diverse genetic underlying, the broad subsets of mutated genes associated with the initial development and progression of HL is often indistinguishable [4].

Management options include surgical treatment of craniofacial abnormalities, hearing aids and cochlear implants, depending on the degree, and type of HL. But for an improved understanding of the pathophysiology and molecular mechanisms of the underlying HL, the promotion of genetic testing in the advancement of the new treatment can be used to a greater extent [5]. It will help in earlier detection of HL, and thereby early intervention can also be initiated, with a better outcome in the development of speech and hearing.

Many studies have experimented on the genetic architecture of NSHL, which predicted the role of many genes. Including some most frequently associated genes such as *GJB2, GJB3, GJB6, SLC26A4, KCNQ4, DFNA5, SLC26A5, MYO1A, MYO7A, MYH15A, CDH23* etc. behind the congenital NSHL [6–9]. Among these, *GJB2* is reported as one of the most prominent causes of congenital genetic NSHL [10].

The improved diagnostic, prognostic, and therapeutic options are the potential translational outcomes of systematic elucidation of NSHL genes [4]. Involvement of gene-encoded proteins in hearing function is expected because the inner ear and hearing mechanism has a very complicated structure [11]. Thus, the present study has focused on exploring some other target genes and most deleterious mutations in genes other than *GJB2*, which might lead to genetic NSHL through systematic review since the last decade (2009–2020) and *in silico* analyses like functional network analysis and variant study.

## Materials and methods

### Search process

A systematic search process followed by ‘Preferred Reporting Items for Systematic Reviews and Meta-Analysis’ (PRISMA) guidelines has been carried out through PubMed (https://pubmed.ncbi.nlm.nih.gov/), ScienceDirect (https://www.sciencedirect.com/), Cochrane Library (https://www.cochranelibrary.com/), and JSTOR (https://www.jstor.org/) search database. Medical subject headings (MeSH) terminologies were searched for ‘Non syndromic hearing loss’ (congenital non syndromic hearing loss (MeSH), non-syndromic hearing loss (MeSH), nonsyndromic hearing loss (MeSH)), ‘genetics’ (gene (MeSH), genetics (MeSH)), AND ‘epidemiology and pathogenesis’ (epidemiology (MeSH), pathogenesis* (MeSH)). All original articles with study design published from the year in last decade till December 2020 were selected.

### Filtering of data

The curated articles had been screened, followed by inclusion and exclusion criteria to satisfy the aim and objectives of the present work.

### Inclusion criteria

All the available full-length original research articles and case reports were included. The following criteria were followed for selecting the articles:

- The information about target genes involved in NSHL experimented on human samples.
- The articles which were written in the English language only.
- The full-length articles published during 2009–2020.

### Exclusion criteria

After that, the rest of the literature were excluded based on the following exclusion criteria:

- The review articles, abstracts, articles written in other languages, letter to the editor, short reports, and correspondences.
- The articles that were based on syndromic HL.
- Articles without any genetic information.

### Curation of data

From the shortlisted articles, the reported target genes in the progression of NSHL have been collected using an electronic spreadsheet. Data were extracted on (1) name of the author; (2) publication year; (3) geographical location; (4) study design; (5) the number of patients; (6) PMID; (7) identified target genes; (8) mode of inheritance. The retrieved target genes have been subjected to duplication removal to get the unique genes for further analyses. After the gene sorting, additional gene information including (1) protein name, (2) UniProt ID, (3) chromosomal location, and (4) chromosome number from UniProt and NCBI Gene databases were collected.

### Network analysis

The network analysis was carried out with the screened unique genes to analyze the functional and the physical association between them through STRING (https://string-db.org/) database [12]. A high confidence score of 0.700 was used to build the network between the genes.

The linked genes were grouped based on their involvement in some of the major NSHL associated biological processes resulted from STRING. The functional protein–protein interaction (PPI) networks were built again at a high confidence score of 0.700 between the grouped genes according to the respective biological processes. The highly interacting co-expressed genes were analyzed and processed further for variant study.

### Variant analysis

The highly interactive and their respective co-expressed genes involved in all four biological processes resulting in STRING were listed for the further variant analysis. The rsIDs of single nucleotide polymorphisms (SNPs) for each gene were collected from dbSNP (https://www.ncbi.nlm.nih.gov/snp/) based on pathogenic clinical significance. Online-based platforms, i.e. SIFT (https://sift.bii.a-star.edu.sg/) [13], PredictSNP1 (PredictSNP, HAPP, PhD-SNP, PolyPhen-1, PolyPhen-2, SIFT, and SNAP used in PredictSNP1) (https://loschmidt.chemi.muni.cz/predictsnp1/) [14] and PredictSNP2 (CADD, DANN, FATHMM, FitCons, FunSeq2 and GWAVA processed through PredictSNP2) (https://loschmidt.chemi.muni.cz/predictsnp2/) [15] were used to get highly deleterious non-synonymous single nucleotide polymorphisms (nsSNPs) according to the scoring algorithms of respective tools. The variants found with deleterious effect in all the used prediction tools will be considered as highly deleterious and the remainings will be discarded from the study.

The novelty of most deleterious nsSNPs was searched in the UniProt (https://www.uniprot.org/) [16] and ClinVar (https://www.ncbi.nlm.nih.gov/clinvar/) [17] database, which stores all the updated information including sequence, structure, variants, functions, publications etc. about proteins.

## Results

### Article selection and gene sorting

**Figure 1:**
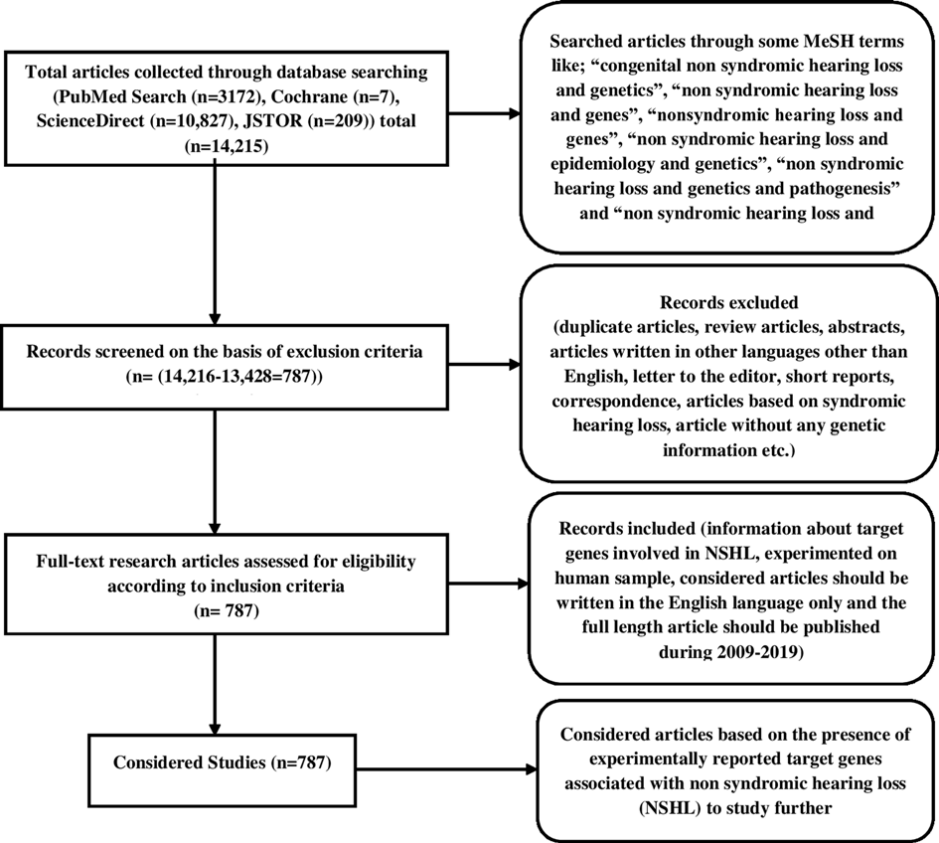
Flowchat of systematic review followed by PRISMA guidelines

The PRISMA guidelines based search methodology identified a total of 14216 articles from the PubMed (3172), JSTOR (209), ScienceDirect (10827), and Cochrane (7) databases based on used MeSH terminologies. Only 787 unique studies, out of 14216 were satisfied all the inclusion criteria and shortlisted for further studies (Figure 1). There was a total of 2707 genes have been collected from 787 original research articles and subjected to duplication removal which resulted 423 unique genes. But among these, mitochondrial RNA (5), DNA (1), and reported miRNAs (7) have been removed, and remaining 382 genes were taken as final unique target genes for network analysis.

### Network analysis

**Figure 2:**
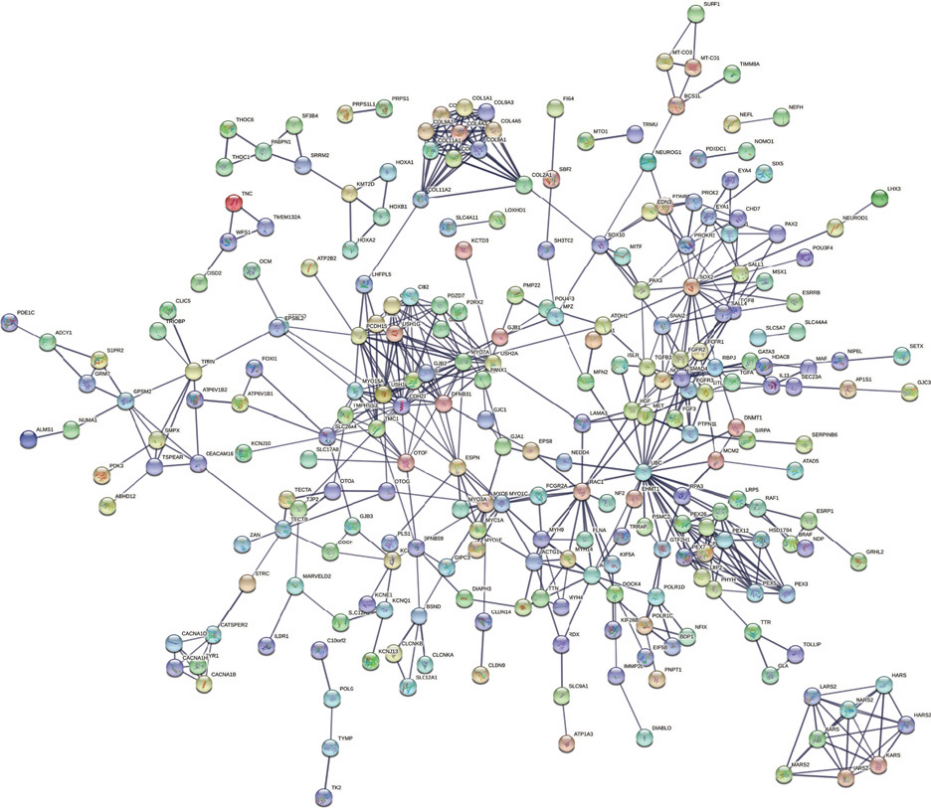
PPI network between all the reviewed target genes (382) through STRING

**Figure 3:**
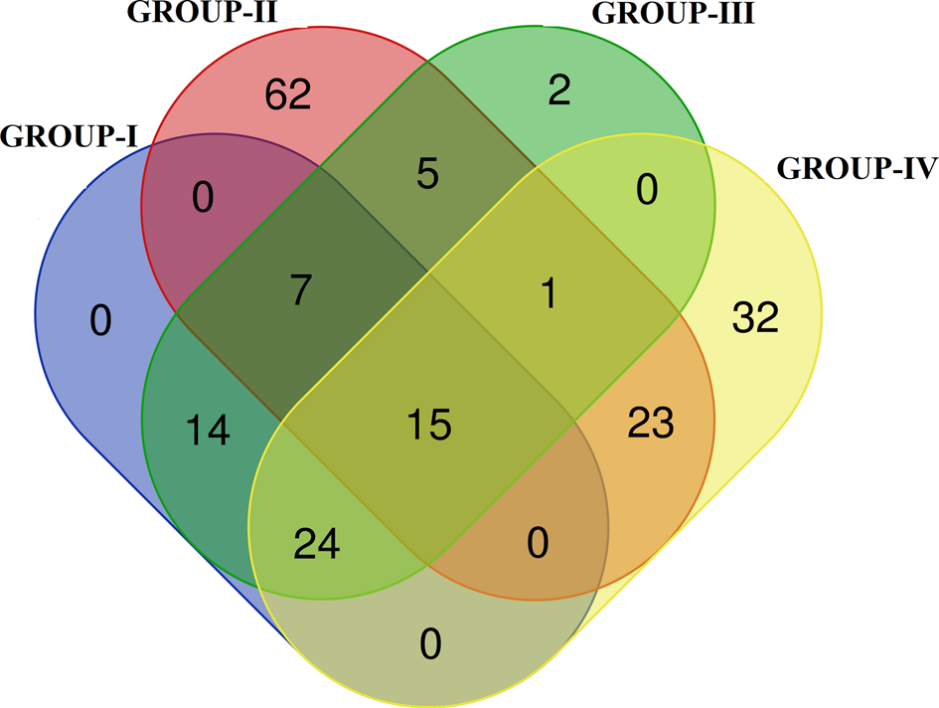
Number of unique and commonly identified genes in four different groups represented through Venn diagram

All the 382 NSHL-associated target genes were subjected to network analysis through the STRING database, which built a strong functional association between the genes with 255 nodes, 571 edges, and PPI with enrichment significant *P*-value <1.0e-16. *UBC* (28 interactions) was the most interacted gene at high confidence score 0.700 (Figure 2), followed by *CDH23* (20 interactions), *SOX2* (19 interactions), *MYO7A* (17 interactions), *PCDH15* (15 interactions) etc. The biological processes resulted in STRING were grouped into four different categories on the basis of important processes associated with HL, i.e. Group I—ear development (ear morphogenesis, inner ear morphogenesis, inner ear receptor cell development, inner ear auditory receptor cell differentiation, inner ear receptor cell stereocilium organization, auditory receptor cell morphogenesis, auditory receptor cell stereocilium organization, cochlea morphogenesis, ear development, outer ear morphogenesis, middle ear morphogenesis, inner ear receptor cell differentiation, cochlea development, vestibulocochlear nerve development, vestibulocochlear nerve formation, auditory receptor cell morphogenesis, auditory receptor cell stereocilium organization, auditory receptor cell development, inner ear development, auditory receptor cell fate commitment); Group II—ion transport (ion transport, cell junction organization, regulation of ion transmembrane transport, cell junction assembly, cell–cell signaling, gap junction assembly, regulation of potassium ion transmembrane transport, potassium ion transmembrane transport, chemical homeostasis, sodium ion transmembrane transport, regulation of cell junction assembly, ion transmembrane transport); Group III—sensory organ development (sensory organ development, sensory organ morphogenesis, sensory system development); and Group IV—sensory signaling pathways (sensory perception of sound, sensory perception, detection of mechanical stimulus involved in sensory perception of sound, response to auditory stimulus) (Table 1). The number of genes were 60, 113, 68, and 95 genes in group I, group II, group III, and group IV, respectively. From the above analysis, it has been observed that there were 62 genes uniquely involved in group II, 2 genes in group III and 32 genes in group IV of biological processes. But there were no unique involvement of genes found in group I of biological processes. Apart from these, there were 15 genes commonly involved in group I, III and IV; where as 7 genes in group I, II and III; 1 gene in group II, III and IV; 23 genes in group II and IV; 14 genes in group I and III; and 5 genes were commonly found in group II and III. Lastly, the involvement of 15 genes was commonly identified in all the four groups of biological processes analyzed in the present study (Table 1 and Figure 3) (Supplementary File S1).

**Table 1:** Studied functional classes and associated target genes involved in NSHL

Function classes	Included functions and GO IDs	Associated genes	Number of genes
Ear development	Ear morphogenesis (GO:0042471)	*USH1C, CLIC5, MYO15A, HOXA2, SLC44A4, ATP6V1B1, SIX1, SALL1, MYO3A, NIPBL, ATOH1, FOXI1, NEUROG1, FGF8, HMX2, EYA1, HOXA1, GJB6, HMX3, LHFPL5, COL11A1, PAX2, POU3F4, GATA3, COL2A1, MSX1, SLITRK6, TRIOBP, MYO7A, CHD7, FGFR1, STRC, FGFR2, USH1G, PDZD7, TMC1, LRTOMT, DFNB31, CDH23, GRXCR1, TPRN, NOTCH1, RBPJ, KCNQ1, POU4F3, TGFB3, MCM2, NEUROD1, SLC26A5, USH2A, SLC17A8, SOX2, MAF, DFNA5, PTPN11, OPA1, ROR1, LHX3, PCDH15, ESRRB*	60
	Inner ear morphogenesis (GO:0042472)		
	Inner ear receptor cell development (GO:0060119)		
	Inner ear auditory receptor cell differentiation (GO:0042491)		
	Inner ear receptor cell stereocilium organization (GO:0060122)		
	Auditory receptor cell morphogenesis (GO:0002093)		
	Auditory receptor cell stereocilium organization (GO:0060088)		
	Cochlea morphogenesis (GO:0090103)		
	Ear development (GO:0043583)		
	Outer ear morphogenesis (GO:0042473)		
	Middle ear morphogenesis (GO:0042474)		
	Inner ear receptor cell differentiation (GO:0060113)		
	Cochlea development (GO:0090102)		
	Vestibulocochlear nerve development (GO:0021562)		
	Vestibulocochlear nerve formation (GO:0021650)		
	Auditory receptor cell morphogenesis (GO:0002093)		
	Auditory receptor cell stereocilium organization (GO:0060088)		
	Auditory receptor cell development (GO:0060117)		
	Inner ear development (GO:0048839)		
	Auditory receptor cell fate commitment (GO:0009912)		
Ion transport	Ion transport (GO:0006811)	*KCNQ1, CLIC5, SLC22A4, SLC52A3, PANX1, SLC44A4, KCNJ13, ATP6V1B1, SLC19A2, RAF1, SLC12A2, KCNQ4, LRP2, SLC9A1, SLC5A7, SLC26A4, ATP6V1B2, GJA1, ANKH, CACNA1D, TMC1, LOXHD1, SLC26A5, SLC17A8, CLCNKA, CACNA1H, KCNE1, P2RX2, RYR1, LHFPL5, ATP2B2, MT-CO1, OPA1, MT-CO3, KCNJ10, SLC17A9, BSND, CACNA1B, SURF1, CLCNKB, SLC4A11, CATSPER2, SLC12A1, CDH23, ABCC1, SCN9A, TRPV4, GJC1, SLC52A2, ATP1A3, TGFB3, GRHL2, MARVELD2, LAMA3, NF2, ACTB, FLNA, GJB1, GJB2, SNAI2, CLDN9, PEAK1, ACTG1, IL13, EDN3, DIAPH1, CHD7, FAM115A, NEDD4, PROKR2, HGF, TP63, TNC, ILDR2, OTOF, LRP5, NEUROD1, MITF, TOLLIP, FGF8, FGF3, FGFR3, WLS, RAC1, GRM7, ROR1, PCDH15, NDP, GATA3, FGFR1, KIF5A, FGFR2, MPZ, UBC, PMP22, WFS1, CIB2, POLG, TMPRSS3, PROK2, ADCY1, HOMER2, C10orf2, MET, PTPN11, SMAD4, GJB6, PAX2, EDNRB, PDK3, BCAP31, DMXL2, MYO1C*	113
	Cell junction organization (GO:0034330)		
	Regulation of ion transmembrane transport (GO:0034765)		
	Cell junction assembly (GO:0034329)		
	Cell–cell signaling (GO:0007267)		
	Gap junction assembly (GO:0016264)		
	Regulation of potassium ion transmembrane transport (GO:1901379)		
	Potassium ion transmembrane transport (GO:0071805)		
	Chemical homeostasis (GO:0048878)		
	Sodium ion transmembrane transport (GO:0035725)		
	Regulation of cell junction assembly (GO:1901888)		
	Ion transmembrane transport (GO:0034220)		
Sensory organ development	Sensory organ development (GO:0007423)	*USH1C, KCNQ1, CLIC5, MYO15A, HOXA2, SLC44A4, POU4F3, ATP6V1B1, MFN2, TGFB3, SIX1, SALL1, GRHL2, MCM2, MYO3A, NOTCH1, NIPBL, GJA1, LRP5, NEUROD1, MITF, TMC1, ATOH1, FOXI1, SLC26A5, LRTOMT, USH2A, SIX5, SLC17A8, NEUROG1, FGF8, SOX2, MAF, DFNA5, PTPN11, HMX2, EYA1, HOXA1, NF2, RBPJ, GJB6, HMX3, LHFPL5, DFNB31, OPA1, COL11A1, PAX2, ROR1, LHX3, POU3F4, PCDH15, NDP, GATA3, COL2A1, MSX1, CDH23, GRXCR1, SLITRK6, TRIOBP, MYO7A, TPRN, CHD7, FGFR1, STRC, FGFR2, ESRRB, USH1G, PDZD7*	68
	Sensory organ morphogenesis (GO:0090596)		
	Sensory system development (GO:0048880)		
Sensory signaling	Sensory perception of sound (GO:0007605)	*USH1C, KCNQ1, CLIC5, MYO15A, SLC52A3, CRYM, COL1A1, WFS1, POU4F3, ATP6V1B1, SIX1, KCNQ4, LRP2, SLC26A4, MYO3A, OTOF, NIPBL, CACNA1D, TMPRSS3, CABP2, TMC1, LOXHD1, SLC26A5, HOMER2, LRTOMT, USH2A, SLC17A8, TSPEAR, MARVELD2, GJC3, CLRN1, KCNE1, DFNA5, EYA1, HOXA1, P2RX2, GJB6, GRM7, LHFPL5, ATP2B2, DFNB31, MYO6, COL11A1, PCDH15, COL11A2, ESPN, GRXCR2, NDP, DCDC2, COL2A1, GJB2, OTOA, PAX3, CCDC50, TECTA, COL4A3, COCH, SNAI2, DIAPH1, CDH23, OTOG, GRXCR1, SLITRK6, TRIOBP, GPR98, MYO7A, DFNB59, TPRN, CHD7, FGFR1, MYO1A, OTOGL, STRC, EYA4, EPS8L2, CEACAM16, MYH14, USH1G, PDZD7, FAM65B, SERPINB6, PROK2, OR51V1, GJB4, OPA1, KCNJ10, PAX2, ROR1, POU3F4, EDNRB, RPGR, SCN9A, GJC1, NEUROG1, ABHD12*	95
	Sensory perception (GO:0007600)		
	Response to auditory stimulus (GO:0010996)		
	Detection of mechanical stimulus involved in sensory perception of sound (GO:0050910)		

**Figure 4:**
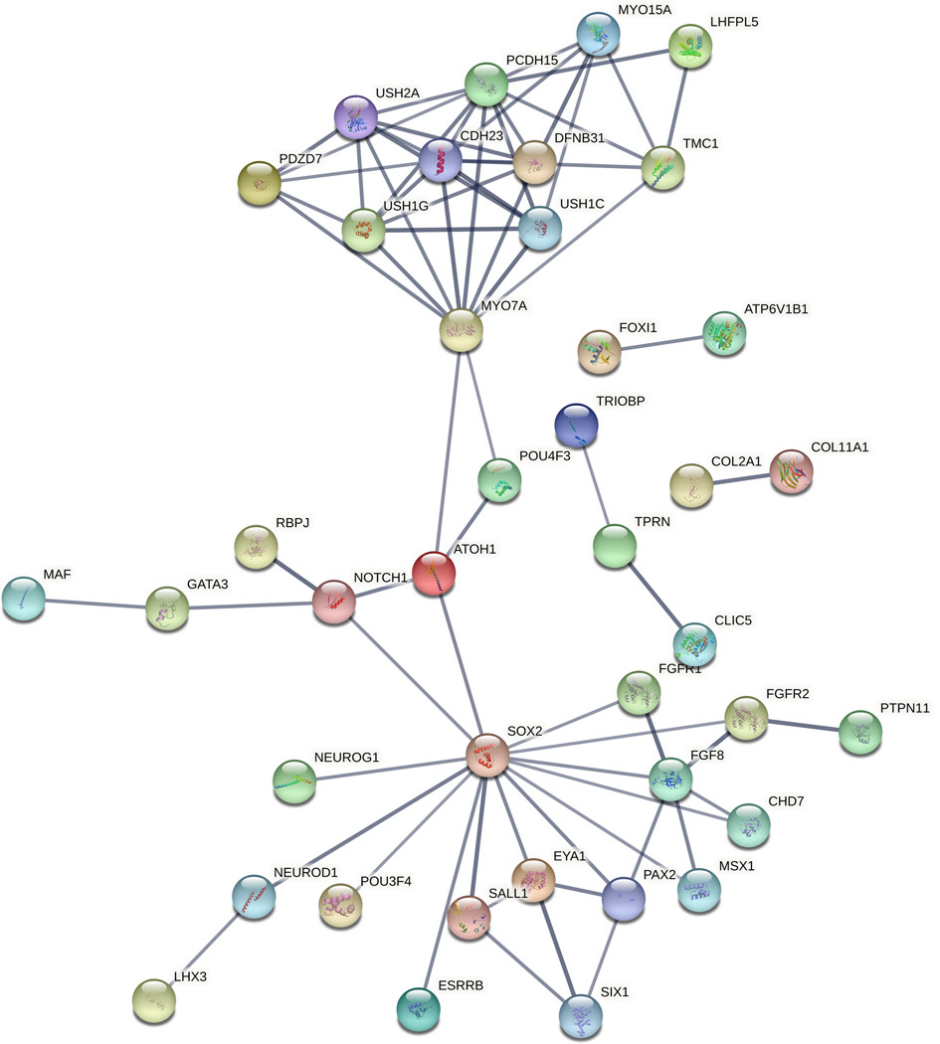
Protein functional network built between ear development group of genes (Group I)

**Figure 5:**
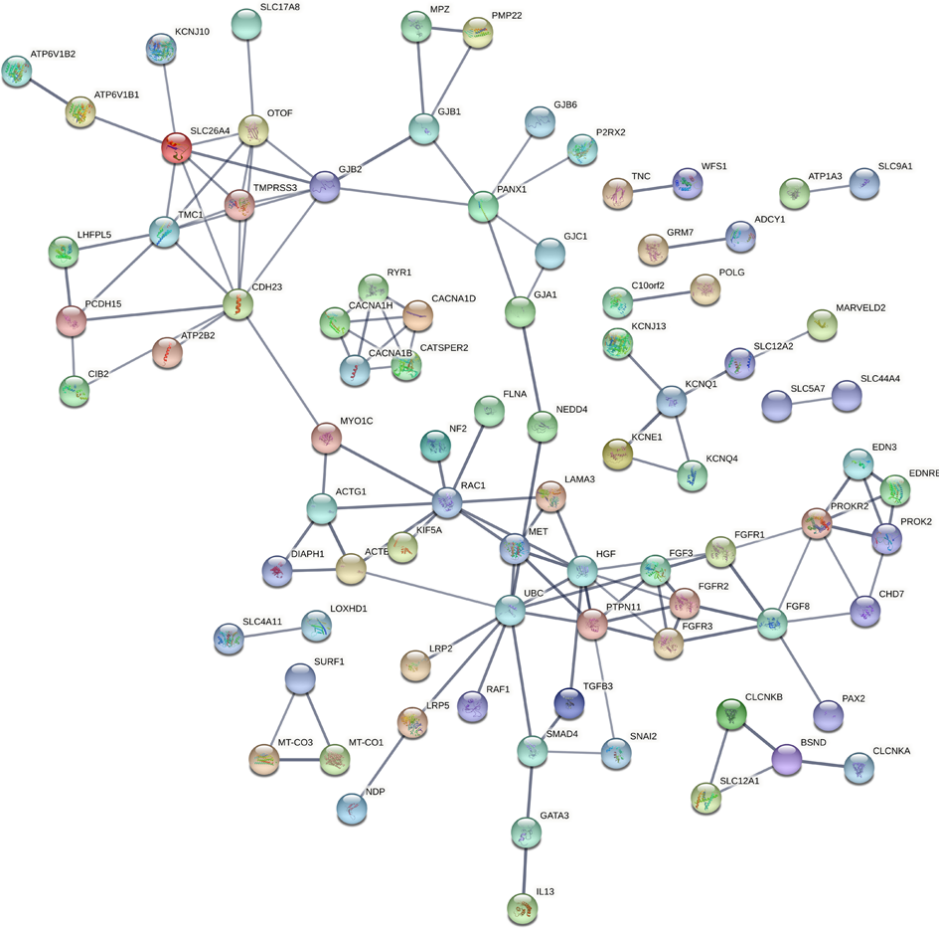
Generated protein interaction network between ion transport group of genes (Group II)

**Figure 6:**
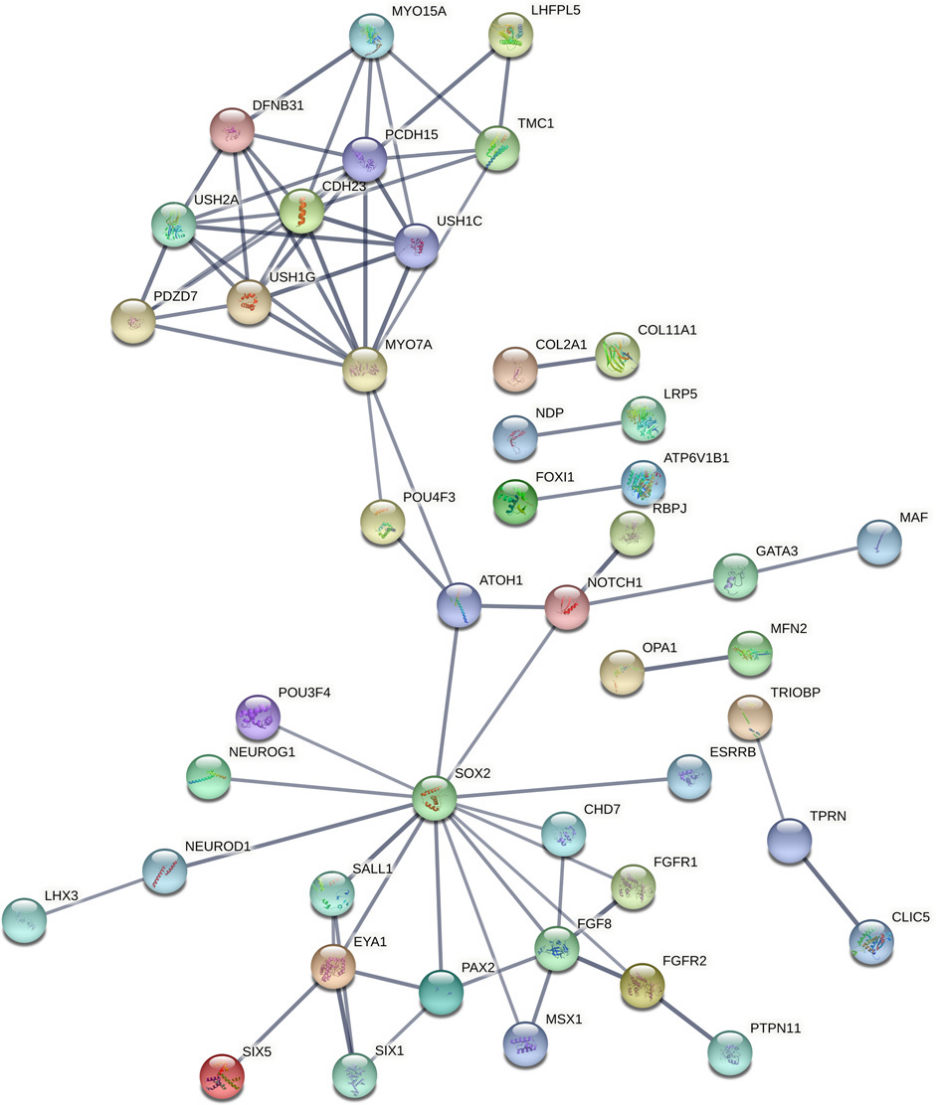
Network represents the strong functional association between sensory organ development group of genes (Group III)

**Figure 7:**
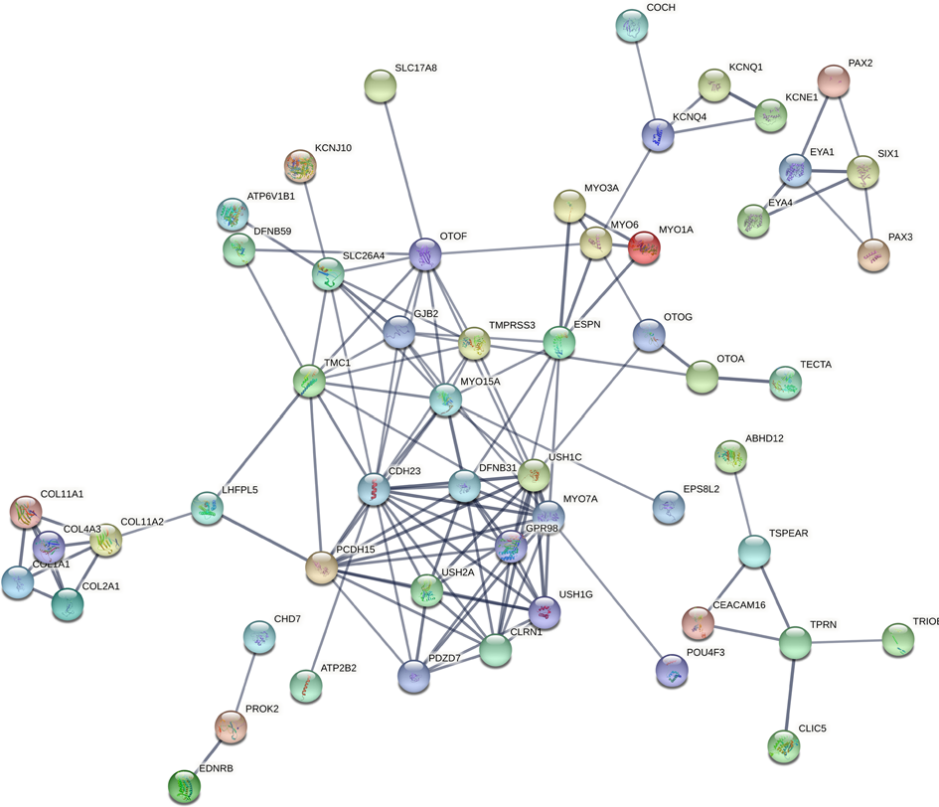
PPI enrichment network of sensory signaling group of genes (Group IV)

These biological processes associated genes have been again processed in networking analysis and generated four individual networks for each group, respectively, at high confidence (0.700). The PPI network in the ear development group of genes was developed with 40 nodes, 72 edges, and <1.0e-16 *P*-value, where *SOX2* (14 interactions) has found as highly interacted gene followed by *MYO7A* and *PCDH15* (10 interactions each) (Figure 4). The ion transport group of genes have built a PPI enrichment network with 86 nodes, 128 edges and <1.0e-16 significant *P*-value, where *HGF* and *UBC* (10 interactions each) have identified as most interacting genes, followed by *CDH23, RAC1* (9 interactions each) (Figure 5). For the sensory organ development group *SOX2* (14 interactions) has found as highly interacted gene, followed by *MYO7A* and *PCDH15* (10 interactions each) in the PPI network built with 45 nodes, 75 edges, and <1.0e-16 significant *P*-value (Figure 6). Lastly between the sensory signaling group genes, the PPI network has been built with 54 nodes, 128 edges, and <1.0e-16 significant *P*-value, where *CDH23* (16 interactions) is the highly interacted gene followed by *MYO7A* (14 interactions) (Figure 7).

From the generated networks it has been observed that, the highly interacted genes *SOX2* has co-expressed with other genes including *EYA1, FGFR1, FGFR2, POU3F4, SALL1* and *CHD7*; likewise gene *MYO7A* has a co-expression with *CDH23* gene in ear development (group I) and sensory organ development (group III) of biological processes. In case of ion transport (group II), both *UBC* and *RAC1* have the co-expression with *ACTB* gene; whereas *CDH23* built a co-expression with *OTOF* and *ATP2B2.* Lastly in sensory signaling category (group IV), the co-expressions found between *CDH23* and three other genes, i.e. *OTOF, MYO7A*, and *ATP2B2;* whereas *MYO7A* has built only one co-expression with *CDH23.* These co-expressed genes of respective biological process groups might be considered as putative genes for NSHL (Table 2).

**Table 2:** Identified highly interacted genes and co-expressed genes in the PPI networks of biological process groups, generated through STRING

Sl. no.	Biological process groups	Highly interacted genes and number of interactions	Co-expressed genes
1	Ear development	*SOX2* (14)	*EYA1, FGFR1, FGFR2, POU3F4, SALL1, CHD7*
		*MYO7A* (10)	*CDH23*
		*PCDH15* (10)	-NA-
2	Ion transport	*HGF* (10)	-NA-
		*UBC* (10)	*ACTB*
		*RAC1* (9)	*ACTB*
		*CDH23* (9)	*OTOF*, *ATP2B2*
3	Sensory organ development	*SOX2* (14)	*EYA1, FGFR1, FGFR2, POU3F4, SALL1, CHD7*
		*MYO7A *(10)	*CDH23*
		*PCDH15* (10)	-NA-
4	Sensory signaling	*CDH23* (16)	*OTOF, MYO7A, ATP2B2*
		*MYO7A* (14)	*CDH23*

### Screening of deleterious SNPs

There were 32, 332, 192, 8, pathogenic SNPs (rsIDs) for the highly interacted genes *SOX2, MYO7A, CDH23, RAC1*, respectively. The number of pathogenic, likely pathogenic SNPs (rsIDs) collected for co-expressed genes identified along with highly interacted genes, i.e. *ACTB, OTOF, ATP2B2, EYA1, FGFR1, FGFR2, POU3F4, CHD7*, and *SALL1* were 55, 122, 4, 53, 99, 86, 29, 370, and 38, respectively (Supplementary File S2). Whereas there was no pathogenic and/or likely pathogenic variant found in dbSNP for the gene *UBC*. These SNPs of 13 genes have been screened in SIFT and predicted deleterious effects in the variants of only 10 genes such as; 4 rsIDs (*SOX2*), 22 rsIDs (*MYO7A*), 28 rsIDs (*CDH23*), 6 rsIDs (*ACTB*), 10 rsIDs (*OTOF*), 5 rsIDs (*EYA1*), 13 rsIDs (*FGFR1*), 23 rsIDs (*FGFR2*), 5 rsIDs (*POU3F4*), and 5 rsIDs *(CHD7)* in respective genes have been predicted as deleterious according to the SIFT score <0.05 (Supplementary File S3), and rest of the other variants of respective genes were not considered further as those found as tolerated. All the deleterious pathogenic variants have again filtered through seven different scoring algorithm-based prediction tools, i.e., PredictSNP, HAPP, PhD-SNP, PolyPhen-1, PolyPhen-2, SIFT, and SNAP simultenously in PredictSNP1 tool. Among the analyzed SNPs, only 3 rsID in *SOX2*, 8 rsIDs in *MYO7A*, 2 rsIDs in *ACTB*, 6 rsIDs in *OTOF*, 1 rsID in *EYA1*, 2 rsIDs in *FGFR1*, 4 rsIDs in *FGFR2*, 5 rsIDs in *POU3F4*, and 1 rsID in *CHD7* have been predicted as deleterious in all the seven tools of PredictSNP1 (Supplementary File S4). But as per the prediction scores the variants of *CDH23* and *RAC1* were not shown deleterious effects in some of the tools used in PredictSNP1, thus these two genes were not considered for deleterious and were not further processed. Lastly the deleterious effects of variants of respective genes have again checked through different prediction tools, i.e. CADD, DANN, FATHMM, FitCons, FunSeq2, and GWAVA processed through PredictSNP2, which resulted in three nsSNPs with rsIDs rs80356586 (Ile^515^Thr), rs80356596 (Leu^1011^Pro), rs80356606 (Pro^1987^Arg) in *OTOF*; two nsSNPs rs121909642 (Pro^722^Ser), rs267606805 (Pro^722^His) in *FGFR1*, and two nsSNPs rs121918506 (Glu^565^Ala), rs121918509 (Ala^628^Thr, Ala^629^Thr) in the gene *FGFR2* have been predicted as deleterious (Table 3) (Supplementary File S5) according to predictSNP2 scoring algorithm. Remaining variants of respective genes were not considered further, as those were not showed deleterious effect in some of the prediction tools used in PredictSNP2.

**Table 3:** Identified most deleterious pathogenic nsSNPs in the target genes of NSHL

Sl. No.	Gene names	UniProt IDs	Most deleterious nsSNPs (deleterious in SIFT, Predict SNP1, PredictSNP2)	Variants and positions
1	OTOF	Q9HC10	rs80356586	Ile^515^Thr
			rs80356596	Leu^1011^Pro
			rs80356606	Pro^1987^Arg
2	FGFR1	P11362	rs121909642	Pro^722^Ser
			rs267606805	Pro^722^His
3	FGFR2	P21802	rs121918506	Glu^565^Ala
			rs121918509	Ala^628^Thr, Ala^629^Thr

All the deleterious nsSNPs in the respective genes *OTOF, FGFR1*, and *FGFR2* have been reported in UniProt and ClinVar databases. But only the nsSNPs rs80356586 (Ile^515^Thr), rs80356596 (Leu^1011^Pro), rs80356606 (Pro^1987^Arg) found in *OTOF* have been reported particularly in NSHL conditions, whereas the nsSNPs rs121909642 (Pro^722^Ser) of *FGFR1* has reported in hypogonadotropic hypogonadism 2 with anosmia, but rs267606805 (Pro^722^His) has not reported in any disease condition. Likewise the nsSNPs of *FGFR2*, rs121918506 (Glu^565^Ala) has reported in Pfeiffer syndrome and craniosynostosis syndrome; and rs121918509 (Ala^628^Thr, Ala^629^Thr) has found in LADD syndrome.

## Discussion

Congenital NSHL has long been considered to be due to genetic mutations. The genetics of NSHL should be explored more to explain the genetic diversity of NSHL. According to previously reported data, *GJB2* is the most frequently associated target gene in NSHL. In the *GJB2* mutant condition, the functional gap junction channel formation is defective [18]. The researchers have been reported the role of *GJB2* mutations in the pathogenesis of NSHL through different experimental analyses [6,19–23]. Though *GJB2* is the most common mutation worldwide in different populations, other important genes include *SLC26A4*, *GJB3*, *GJB6*, *MYO15A*, *MYO7A*, *TMC1*, *CDH23* etc. were also identified in the pathogenesis of NSHL [24–28]. This study reviewed all the described target genes identified experimentally in NSHL during the period between 2009 and 2020 and attempted to study the interactions between them through functional network analysis and identify the possible deleterious variants.

The congenital HL may be due to the defects in the outer, middle (conductive), and inner ear (sensorineural) [18]. The sensorineural type of HL involves multiple mechanisms due to genetic defects leading to abnormal biocellular processes. From the resulted functional network at high confidence through STRING the association between the identified target genes on the basis of biological processes has been analyzed. This provided an opportunity to identify further vital genes and their underlying mechanisms for ear development and morphogenesis (dysfunction of ear and/or cochlea; abnormality in the structural morphogenesis or transformation of ear) [18,29]; ion homeostasis/transport [30], auditory sensory system/sensory signaling [31] which are causative towards disease pathology. The system biology approach has provided the advantages of gene regulatory network analysis to understand the interactive roles of genes in disease pathogenesis [32]. However, the gene–gene interaction network describes the close connections between the genes in a particular pathway, which is easier to interpret and validate the research objectives [33]. Previous studies have reported some other genes as the gene hub in the PPI networks through *in silico* applications; such as Fan et al., in 2014 has identified *TMPRRS3* (interacted with *GJB2, SLC26A4, MYO7A, DFNB59*) as the most interacted target gene in NSHL through network analysis (between 98 genes) by using STRING 9.0 [34]. Likewise, *MYO7A* (interacted with *MYO6, KCTD3, NUMA1, MYH9, KCNQ1, UBC, DIAPH1, PSMC2*, and *RDX*) has reported as the most interacted central gene hub in the PPI network (between 116 HL genes) generated through Enricher and PANTHER databases by Lebeko et al. in 2017 [35]. In another study, the network analysis was done between three groups of HL genes [(i) nonsyndromic group of genes (63 genes), (ii) syndromic or non-syndromic group (107 genes), and (iii) -otic capsule development and malformation group of genes (112 genes)]. By using the ingenuity pathway analysis (IPA) software, from which *TGFB1* (with 35 connections) was found in the central node of the network in the first group (NSHL group of genes) and *MAPK3/MAPK1 MAP* kinase (with 33 gene connections) was identified as the central node of the network in the second group (both syndromic and NSHL group of genes) [23]. Thus, this present study has tried to explore the gene network analysis through STRING database with 382 genes involved in NSHL at high confidence score and identified the highly interacted HL target genes *UBC, HGF, CDH23, RAC1, SOX2, MYO7A*, and *PCDH15* in each of the four biological processes groups have been chosen as gene hub in the NSHL target gene panels. In addition to these hub genes, the co-expressed target genes are also important in disease pathogenesis. The co-expressed networks can identify the possible gene pairs, regulatory genes, similar gene matrixes etc., in the disease conditions, which indicates the simultaneously active target genes in the disease progression [36]. Thus, the present study has also chosen the associated co-expressed genes *ACTB, OTOF, ATP2B2, EYA1, FGFR1, FGFR2, POU3F4, CHD7*, and *SALL1*, along with the target hub genes in all possible functional groups of hearing impairment.

However, it has been reported that inner ear dysfunction is a relatively common consequence of human genetic mutation [37]. In these genetic mutations, the nsSNPs have a vital role in damaging or modifying protein-coding sites, consequently affecting the protein’s structure and function [38]. The analysis of most functionally interactive genes for possible pathogenic non-synonymous variants could identify only in *OTOF, FGFR1*, and *FGFR2* genes through *in silico* tools based on scoring algorithms. The expression of these three genes and their mutational effects in the progression of NSHL has been reported since the last decade. Some of the pathogenic (Arg^798^X, Gly^829^X, Leu^391^Arg, Glu^747^X, Arg^425^X, Tyr^474^X, Trp^717^X, Tyr^1064^X, Gln^1072^X, Arg^1856^Gln, Arg^1172^Gln) and likely pathogenic (Pro^489^Ser, His^513^Arg, Arg^1583^His, Arg^1792^Cys, Arg^1792^His) variants in *OTOF* have been reported in autosomal recessive NSHL cases of Texas, Qatar, and Japan populations [9,39,40].

The pathogenic nsSNPs for all the highly interacted and co-expressed genes have been collected from dbSNP and analyzed in SIFT, PredictSNP1, and PredictSNP2. Among the analyzed nsSNPs, only some nsSNPs rs80356586, rs80356596, rs80356606 in *OTOF*, rs121909642 in *FGFR1*, and rs121918506, rs121918509 in *FGFR2* have been identified as deleterious in all the prediction algorithms. So these variants can be called as most deleterious SNPs in the respective genes, which might leads to NSHL. The gene *OTOF* is responsible for the composition of ribbon synaptic vesicles in cochlear inner hair cells, and the mutations in *OTOF* are responsible for 2–3% of NSHL [9,41]. The association of *OTOF* in NSHL has been experimented in immortal lymphoblastoid cell lines, inner hair cell (IHC) and human embryonic kidney cells (HEK) [42,43]. In this study, the most deleterious nsSNPs rs80356586 (Ile^515^Thr) [44], rs80356596 (Leu^1011^Pro) [45,46], rs80356606 (Pro^1987^Arg) [47] found in *OTOF* gene were reported in UniProt and ClinVar datasets for NSHL cases. Among these three variants, two have been identified in the Turkish population (Ile^515^Thr, Leu^1011^Pro) and one in northern Lebanon populations (Pro^1987^Arg).

The involvement of *FGFR1* has been reported in the development of the auditory sensory epithelium *in vitro* studies on mice [48]. FGFR1 and FGFR2 have also been used in the reference gene panel, which has been used in the genomic diagnosis of NSHL cases in the Spain population earlier [42]. No reports have described either the role of *FGFR1* and *FGFR2*, or on presently predicted nsSNPs rs121909642 (Pro^722^Ser) of *FGFR1* and rs121918506 (Glu^565^Ala), rs121918509 (Ala^628^Thr, Ala^629^Thr) of *FGFR2* in the NSHL in humans.

However, the variants of *FGFR1* and *FGFR2* have been found in hypogonadotropic hypogonadism 2 with anosmia (rs121909642) [49], Pfeiffer syndrome [50], craniosynostosis syndrome [51] (rs121918506), and in LADD syndrome (rs121918509) [52,53], respectively. No disease was found for the variant rs267606805 (P722H) in the ClinVar database. The unreported variants in *FGFR1* and *FGFR2* in NSHL cases indicates the uniqueness of the predicted results.

These deleterious variants might have structural and functional effects on respective proteins, leading to NSHL. Thus, the found variants in the respective gene could be considered potential targets for NSHL after clinical authentication.

## Conclusion

Genetic counselling remains a crucial analysis for patients with NSHL. The advancement in *in-silico* tools and techniques, including GWAS and NGS technologies, are excellent resources for the research community in the present and future research. The present study’s findings, including analyzed biological process-based gene networks and evaluated pathogenic variants in target genes of NSHL would provide some novel insights into further genetic research. This could help in the generation of novel and advanced prediction and diagnosis of NSHL.

## Supplementary Material

Supplementary File S1-S6

## Data Availability

All data generated or analyzed during the present study are included in this article.

## Abbreviations

GWAS: Genome Wide Association Studies
HL: hearing loss
MeSH: Medical Subject Headings
NGS: Next Generation Sequencing
NSHL: non-syndromic hearing loss
nsSNP: non-synonymous single nucleotide polymorphism
PPI: protein–protein interaction
PRISMA: Preferred Reporting Items for Systematic Reviews and Meta-Analysis
