# Supplementary material for: Comprehensive functional network analysis and screening of deleterious pathogenic variants in non-syndromic hearing loss causative genes
Source: Biosci Rep. 2021 Oct 29;41(10):BSR20211865. doi: 10.1042/BSR20211865 (PMC8559308; doi:10.1042/BSR20211865)
Supplement: Supplementary File S1-S6 [file BSR-2021-1865_supp.zip › BSR-2021-1865_suppS6.docx]

| **Section and Topic** | **Item #** | **Checklist item** | **Location where item is reported** |
| --- | --- | --- | --- |
| **TITLE** | | |  |
| Title | 1 | Comprehensive Functional Network Analysis and Screening of Deleterious Pathogenic Variants in Non-Syndromic Hearing loss Causative Genes | Title Page (Line Number 2-3) |
| **ABSTRACT** | | |  |
| Abstract | 2 | Hearing loss is a significant public health problem and causes the most frequent congenital disability in developed societies. The genetic analysis of non-syndromic hearing loss (NSHL) may be considered as a complement to the existent plethora of diagnostic modalities available. This study focuses on exploring more target genes with respective non-synonymous single nucleotide polymorphisms (nsSNPs) involved in the development of NSHL. The functional network analysis and variant study have successfully been carried out from the gene pool retrieved from reported research articles of the last decade. The analyses have been done through STRING. According to predicted biological processes, various variant analysis tools have successfully classified the NSHL causative genes and identified the deleterious nsSNPs, respectively. Among the predicted pathogenic nsSNPs with rsIDs rs80356586 (I515T), rs80356596 (L1011P), rs80356606 (P1987R) in OTOF have been reported in NSHL earlier. The rs121909642 (P722S), rs267606805 (P722H) in FGFR1, rs121918506 (E565A) and rs121918509 (A628T, A629T) in FGFR2 have not reported in NSHL yet, which should be clinically experimented in NSHL. This also this indicates this variant’s novelty as its association in NSHL. The findings and the analysed data have delivered some vibrant genetic pathogenesis of NSHL. This data might be used in the diagnostic and prognostic purposes in non-syndromic congenitally deaf children. | Line Number 25-42 |
| **INTRODUCTION** | | |  |
| Rationale | 3 | Hearing impairment has the highest age-standardized disability globally. It affects nearly 1 in every 1000 livelihoods worldwide. Hearing impairment affects speech development and language acquirement and hinders children's education. The causes of hearing loss (HL) can broadly be classified as conductive, sensorineural, and mixed hearing loss. HL which is predominantly due to genetic aetiology, usually present in an early life without any additional clinical phenotypes.  70% of neonates with HL are presumed to have inherited HL, classified as non-syndromic hearing loss (NSHL). They are not associated with other distinguishing physical findings. NSHL generally follows simple Mendelian inheritance with a 75-80% transmission rate as autosomal recessive, 20% as autosomal dominant, 25% as X-linked, and remaining 1% as mitochondrial mutation. 50% of congenital sensorineural hearing loss is hereditary, caused by genetic mutations of a single gene or combination of multiple genes.  The explosions of genetic information and advancement in technology have radically improved the deep understanding of inherited diseases. In the case of NSHL, genetic correlations are a significant challenge due to wide clinical and genetic heterogeneity. Due to the diverse genetic underlying, the broad subsets of mutated genes associated with the initial development and progression of hearing loss is often indistinguishable.  Management options include surgical treatment of craniofacial abnormalities, hearing aids, and cochlear implants, depending on the degree, and type of hearing loss. But for an improved understanding of the pathophysiology and molecular mechanisms of the underlying hearing loss, the promotion of genetic testing in the advancement of the new treatment can be used to a greater extend. It will help in earlier detection of hearing loss, and thereby early intervention can also be initiated, with a better outcome in the development of speech and hearing.  The improved diagnostic, prognostic, and therapeutic options are the potential translational outcomes of systematic elucidation of NSHL genes. Involvement of gene-encoded proteins in hearing function is expected because the inner ear and hearing mechanism has a very complicated structure. Thus the present study has focused on exploring some other target genes and mutations other than GJB2, which might lead to genetic NSHL through systematic review since the last decade (2009-2020) and in silico analyses like functional network analysis and variant study. | Introduction (Line Number 52-88) |
| Objectives | 4 | To identify the potential target gene for NSHL  To isolate the most deleterious variant in the NSHL associated target gene | Introduction (Line Number 85-88) |
| **METHODS** | | |  |
| Eligibility criteria | 5 | Inclusion Criteria:   1. The information about target genes involved in NSHL experimented on human sample. 2. The articles which are written in the English language only. 3. The full-length article published during 2009-2020.   Exclusion Criteria:   1. The review articles, abstracts, articles written in other languages, letter to the editor, short reports and correspondences. 2. The articles that are based on syndromic hearing loss. 3. Article without any genetic information. | Material & Methods (Line Number 103-116) |
| Information sources | 6 | PubMed (<https://pubmed.ncbi.nlm.nih.gov/>) (Last searched Date: 11/10/2021)  ScienceDirect (<https://www.sciencedirect.com/>) (Last searched Date: 06/2021)  Cochrane Library (<https://www.cochranelibrary.com/>) (Last searched Date: 06/2020)  JSTOR (<https://www.jstor.org/>) (Last searched Date: 06/2020)  STRING (<https://string-db.org/>) (Last searched Date: 6/10/2020)  SIFT (<https://sift.bii.a-star.edu.sg/>) (Last searched Date: 6/01/2021)  PredictSNP1 (<https://loschmidt.chemi.muni.cz/predictsnp1/>) (Last searched Date: 29/01/2021)  PredictSNP2 (<https://loschmidt.chemi.muni.cz/predictsnp2/>) (Last searched Date: 25/02/2021)  UniProt (<https://www.uniprot.org/>) (Last searched Date: 26/02/2021)  ClinVar (<https://www.ncbi.nlm.nih.gov/clinvar/>) (Last searched Date: 8/10/2021) | Materials and Method  (Line number 89-150) |
| Search strategy | 7 | PubMed: Used MeSH terms for search, Publication Year: 2009-2020  ScienceDirect: Used MeSH terms for search, Publication Year: 2009-2020  Cochrane Library: Used MeSH terms for search, Publication Year: 2009-2020  JSTOR: Used MeSH terms for search, Publication Year: 2009-2020  STRING: Putted multiple genes for organism Homo Sapiens with criteria high confidence score 0.700  dbSNP: Searched through gene names with search criteria pathogenic/likely pathogenic clinical significance  SIFT: The rsIDs of single nucleotide polymorphisms were putted  PredictSNP1: The FASTA sequence of target genes and the deleterious amino acid variants were given (Result with effective for all tools used in PredictSNP1 were taken into consideration)  PredictSNP2: The CHR,START,[END,]REFERENCE_ALLELE,ALTERNATIVE_ALLELE in simple format (eg:12,112888172,A,G) of all the effective variants (resulted from PredictSNP1) were analysed against human genome assembly GRCh27/hg19 (Result with deleterious effect for all tools used in PredictSNP2 were taken into consideration)  UniProt: Searched the variant through the name of the target gene.  ClinVar: Searched the name of the gene with the particular variant in the search box. According to the variant we have choose the reference PMID. | Materials and Method  (Line number 89-150) |
| Selection process | 8 | PRISMA method was used to decide whether a study met the inclusion criteria of the review.  3 reviewers independently screened each record and each report retrieved. | Materials and Method  (Line number 152-160) |
| Data collection process | 9 | There is no specific method used to collect data from reports.  3 reviewers collected data from each report independently.  NA | NA |
| Data items | 10a | **Methods used to decide which results to collect:**  Inclusion Criteria for article search  Protein-protein interaction network with high confidence score 0.700  Functional enrichment or biological processes for grouping genes  Variant Analysis:  dbSNP (for collection of pathogenic variants; SIFT (for isolate deleterious variants); PredictSNP1 (for identifying effective variants) and PredictSNP2 (for identifying most deleterious variants on human genomic region) |  |
|  | 10b | NA |  |
| Study risk of bias assessment | 11 | NA |  |
| Effect measures | 12 | NA |  |
| Synthesis methods | 13a | NA |  |
|  | 13b | NA |  |
|  | 13c | NA |  |
|  | 13d | NA |  |
|  | 13e | NA |  |
|  | 13f | NA |  |
| Reporting bias assessment | 14 | NA |  |
| Certainty assessment | 15 | NA |  |
| **RESULTS** | | |  |
| Study selection | 16a |  | Results  Figure 1 (Line Number 156) |
|  | 16b | NA |  |
| Study characteristics | 17 | \| **PubMed ID** \| **Gene Name** \| **Type of Hearing loss** \|  \| **Location** \| **No. of implants** \| \| --- \| --- \| --- \| --- \| --- \| --- \| \| 19707039 \| GJB2 \| Non syndromic \|  \| China \| 115 \| \| 19509082 \| SLC26A4 \| Non syndromic \|  \| China and US \| 116 \| \| 19438934 \| TMIE \| Non syndromic \| Autosomal Recessive \| southeastern Anatolia \| 254 families \| \| 19338775 \| TRMU and MTO1 \| Non syndromic \| Mitochondrial \| Brazil \| 85 \| \| 19157576 \| GJB2 \| Non syndromic \| Autosomal Dominant \| Kerla, India \| 86 and 59 families \| \| 19027181 \| GJB2 \| Non syndromic \| Autosomal Recessive \| Bogotá, Colombia \| 731 \| \| 18985073 \| GJB2 \| Non syndromic \| Autosomal Recessive \| Europe and North America \| 1277 \| \| 18813951 \| SLC26A4 \| Non syndromic \| Autosomal Recessive \| Iran \| 12 families \| \| 21193157 \| POU3F4 \| Non syndromic \| X-linked \| China \| 11 \| \| 21112098 \| GJB2 (p.R32S and p.P225L) \| Non syndromic \| Autosomal Recessive \| Japan \| 126 \| \| 20236118 \| GJB2, GJB6 \| Non syndromic \| Autosomal Recessive \| German-America \| 528 \| \| 20127034 \| GJB2, SLC26A4 \| Non syndromic \|  \| Korea \| 51, 65 \| \| 21996152 \| GJB2 \| Non syndromic \| Sensoneural hearing loss \| **Turkey** \| **65** \| \| 21468573 \| GJB2 (35delG, 167delT, M34T, W24X, W77R and E47X ) \| Non syndromic \| Autosomal Recessive \| Syria \| 50 families \| \| 21454591 \| TMPRSS3 (Y260X) \| Non syndromic \| Autosomal Recessive \| \|  \| \| 21802533 \| ESRRB \| Non Syndromic \| Autosomal Recessive \| Pakistani and Turkish \| 127 families \| \| 22106692 \| GJB2, GJB6 \| Non Syndromic \|  \| Malaysia \| 138 \| \| 21586435 \| GJB2 \| Non Syndromic \| Sensoneural hearing loss (recessive) \| British pakistani, British white \| 177 \| \| 21150918 \| MYO7A \| Both \| Autosomal Dominant \| China \| 2 families \| \| 21916817 \| GJB2, GJB3, GJB6 \| Non Syndromic \|  \| Campania region of southern Italy \| 129 affected, 5 control \| \| 21651318 \| KCNQ4, GJB3 \| Non Syndromic \| Autosomal Dominant \| China \| a four generation family \| \| 21738759 \| GJB2 \| Non Syndromic \| Sensoneural \| Colombia \| 3 control, 5 proband \| \| 21204229 \| COL11A2 \| Non Syndromic \| Autosomal Recessive \| Turkey \| 2 \| \| 21917135 \| SLC26A4 \| Non Syndromic \|  \| Uyghur, Han China \| 199, 151 \| \| 21298213 \| GJB2 \| Non Syndromic \|  \| Korea \| 412 \| \| 21356526 \| DIAPH1, WFS1 \| Non Syndromic \| Sensoneural hearing loss \| China \| 5 generation chinease family \| \| 23053991 \| LRTOMT (c.242G>A) \| Non syndromic \| Autosomal Recessive \| Moroccan \| 105 families, 120 control \| \| 23039283 \| C×26 35delG (GJB2) \| Non syndromic \|  \| Sweden \| 79 \| \| 22899989 \| CDH23 \| Non syndromic \| Autosomal Recessive \| Japan \| 304 \| \| 22717225 \| SLC26A4 \| Non syndromic \| Autosomal Recessive \| Italy \| 2 \| \| 22147502 \| STRC \| Non syndromic \| Autosomal Recessive \| Philadelphia, US, Ein Kerem \| 669 \| \| 22070872 \| GJB2 and GJB6 \| Non syndromic \|  \|  \|  \| \| 22037723 \| GJB2 (R127H, W24X) \| Non syndromic \| Sensoneural \| Eastern Roman \| 45 \| \| 22613756 \| GJB2 \| Non Syndromic \|  \| Kelantan, Malaysia \| 91 \| \| 22382023 \| TMPRSS3 \| Non Syndromic \| Autosomal Recessive \| Morocco \| 80 families \| \| 22186156 \| GJB6 \| Non Syndromic \|  \| China \| 665 \| \| 22281373 \| GJB2, GJB6 \| Non Syndromic \|  \| Slovak \| 273 \| \| 22484064 \| GJB2 \| Non Syndromic \|  \| Greek \| 1 \| \| 21534946 \| TMPRSS3 \| Non Syndromic \| Autosomal Recessive \| Pakistan \| 10 \| \| 23185506 \| SLC26A4 \| Non Syndromic \|  \| China \| 2352 \| \| 22246673 \| CLDN14 \| Non Syndromic \| Autosomal Recessive \| Pakistan \| 4 families \| \| 22695344 \| GJB2 \| Non Syndromic \| Autosomal Recessive \| Iran \| 2322 \| \| 22567152 \| GJB2 \| Non Syndromic \|  \| Belarus \| 391 cases, 757 control \| \| 21348867 \| GPSM2 \| Non Syndromic \| Autosomal Recessive \| Palestine \| 1 family \| \| 22208444 \| GJB2 \| Non Syndromic \|  \| Iran \| 2 families \| \| 22321824 \| GJB2 \| Non Syndromic \|  \| Greek \| 1 \| \| 23073770 \| GJB2 \| Non Syndromic \| Autosomal Recessive \| Iran \| 50 \| \| 23151025 \| SLC26A4 \| Non Syndromic \| Autosomal Recessive \| China \| 80 \| \| 23302201 \| SLC26A4 \| Non Syndromic \|  \| China \| 1 family \| \| 22951369 \| ESRRB \| Non Syndromic \| Autosomal Recessive \| Czechia \| 1 family \| \| 22544735 \| CEACAM16 \| Non Syndromic \| Autosomal Dominant \| Munich \| 1 \| \| 23141447 \| GJB2, SLC26A4 \| Non Syndromic \|  \| China \| 336 \| \| 22938506 \| ACTG1, MYO1F, DIAPH1, POU4F3, EYA4 \| Non Syndromic \| Autosomal Dominant \| Korea \| 8 families \| \| 22097895 \| MARVELD2 \| Non Syndromic \| Autosomal Recessive \| Czech and Central European \| 40 \| \| 26011646 \| STRC \| Non syndromic \| Autosomal Recessive \| Germany \| 95 \| \| 23856379 \| GJB2 (c.405delC ) \| Non syndromic \| Autosomal Recessive \| Tunisia \| 2 \| \| 23856378 \| GJB2 (p.V37I (c.109G > A) and p.R143Q (c.428G > A) \| Non syndromic \| Autosomal Dominant \| Tunisia \| 1 \| \| 23171692 \| GJB2-35delG \| Non syndromic \| Autosomal Recessive \| Turkey \| 94 \| \| 23590985 \| CLDN14 \| Non Syndromic \| Autosomal Recessive \| Morocco \| 80 families \| \| 23273637 \| SLC26A4 \| Non Syndromic \| Autosomal Recessive \| Brazil \| 23 \| \| 23141803 \| GJB2, GJB4, GJA1, GJC3 \| Non Syndromic \| Autosomal Recessive \| Iran \| 34 \| \| 23503914 \| GJB2, GJB6 \| Non Syndromic \|  \| Amazon \| 77 \| \| 23179405 \| GJC3 \| Non Syndromic \|  \|  \|  \| \| 24506997 \| GJB2 \| Non Syndromic \|  \|  \|  \| \| 23750663 \| KCNQ4 \| Non Syndromic \| Autosomal Dominant \| \|  \| \| 23958653 \| TMPRSS3 \| Non Syndromic \| Autosomal Recessive \| Korea \| 40 \| \| 23767834 \| MYO15A, GPR98, TMC1, USH2A, PCDH15 \| Non Syndromic \| Autosomal Recessive \| China \| 190 \| \| 24346070 \| GJB2 \| Non Syndromic \| Autosomal Recessive \| Chile \| 47 \| \| 23523375 \| TMC1 \| Non Syndromic \| Autosomal Recessive \| Iran \| 159 \| \| 23635807 \| MYO6 \| Non Syndromic \| Autosomal Dominant \| Germany \| 1 family \| \| 24260153 \| POU4F3 \| Non Syndromic \| Autosomal Dominant \| Korea \| 1 \| \| 24339547 \| CLDN14, KCNE1 \| Non Syndromic \| Autosomal Recessive \| Morocco \| family \| \| 24551789 \| LRTOMT \| Non Syndromic \| Autosomal Recessive \| Azarbaijan Sharghi, Kordestan, Gilan and Golestan provinces, north and west of Iran, were ascertained \| 157 \| \| 23990876 \| WFS1, COCH, EYA4, MYO6, GJB3, COL11A2, OTOF, STRC, MYO3A \| Non Syndromic \| Autosomal Recessive \| Seoul, Korea \| 145 \| \| 24158611 \| GJB2, GJB6 \| Non Syndromic \|  \| Argentina \| 476 \| \| 23383098 \| MYO7A \| Non Syndromic \| Autosomal Dominant \| China \| 9 \| \| 26171365 \| GJB2 (C202R) \| Non syndromic \| Autosomal Dominant \| Iran \| 1 \| \| 25474651 \| TMPRSS3: c.535G>A \| Non syndromic \| Autosomal Recessive \| Tibet \| 1 \| \| 25218342 \| GJB2, GJB6 \| Non syndromic \|  \| Ibadan, Nigeria \| 81 \| \| 25175280 \| POU3f4 \| Non syndromic \| X-linked \| China \| 4 \| \| 25149889 \| Cx26 \| Non syndromic \| Autosomal Recessive \| Iran \| 1 \| \| 24878468 \| HOXA1 \| Non syndromic \|  \| Middle Eastern population \| 48 \| \| 24793888 \| GJB2, GJB6 \| Non syndromic \|  \| Sicily \| 102 \| \| 24768815 \| ILDR1 \| Non syndromic \| Autosomal Recessive \| Saudi \| 1 family \| \| 24741995 \| EPS8 \| Non syndromic \| Autosomal Recessive \| \|  \| \| 24224790 \| GJB2 \| Non syndromic \|  \| Iran \| 4 \| \| 25458163 \| TMC1 \| Non Syndromic \| Autosomal Recessive \| Tibetan China \| 2 \| \| 24933359 \| DFNA5 \| Non Syndromic \| Autosomal Dominant \| China \|  \| \| 24507663 \| GJB2 \| Non Syndromic \|  \| China \| 619 probands \| \| 24211385 \| P2RX2 \| Non Syndromic \| Autosomal Dominant \| China \|  \| \| 24080506 \| GJB2 (Cx26) \| Non Syndromic \| Autosomal Recessive \| Kuwait \| 100 \| \| 24752540 \| TJP2, CLDN14 \| Non Syndromic \|  \| Korea \| 135 \| \| 24337325 \| GJB2 \| Non Syndromic \|  \| East Asia \|  \| \| 24949729 \| GJB2, MYO15A, TMC1, BSND, TMPRSS3 \| Non Syndromic \| Autosomal Recessive \| Pakistan \| 30 families \| \| 24737404 \| GJB2, SLC26A4 \| Non Syndromic \| Autosomal Recessive \| China \| 176 \| \| 24933359 \| DFNA5 \| Non Syndromic \| Autosomal Dominant \| China \| 1 family \| \| 25250959 \| WFS1 \| Non Syndromic \| Autosomal Dominant \| China \| 6 generation family \| \| 24767429 \| CDH23 \| Non Syndromic \| Autosomal Recessive \| Korea \| 16 \| \| 25242383 \| EYA4 \| Non Syndromic \| Autosomal Dominant \| China \| 1 family \| \| 24007330 \| SLC26A4 \| Both \|  \| East Asia \| 111 \| \| 24359977 \| GJB2 \| Non Syndromic \|  \| China \| 29 probands \| \| 25116015 \| KCNQ4 \| Non Syndromic \| Autosomal Recessive \| China \| 66 \| \| 24926664 \| LRTOMT \| Non Syndromic \| Autosomal Recessive \| Tunis \| 2 \| \| 24211385 \| P2RX2 \| Non Syndromic \| Autosomal Dominant \| China \|  \| \| 25015771 \| SLC26A4 \| Non Syndromic \|  \| China \| 7 families \| \| 24416283 \| TMPRSS3, TMC1, USHIC, CDH23, TMIE \| Non Syndromic \| Autosomal Recessive \| India \| 1739 \| \| 25080041 \| MYO1A, MYO6 \| Non Syndromic \| Autosomal Dominant \| Korea \| 53 \| \| 25317404 \| SLC26A4 \| Non Syndromic \| Autosomal Recessive \| Iran \| 30 families \| \| 24550759 \| ATE1, SLC12A1 \| Non Syndromic \|  \| USA \|  \| \| 25505834 \| MYH9 \| Non Syndromic \| Autosomal Dominant \| Brazil \| 10 \| \| 24941117 \| GJB2, SLC26A4 \| Non Syndromic \|  \| China \| 155 \| \| 24774219 \| GJB2, GJB6 \| Non Syndromic \|  \| Mexico \| 26 \| \| 24482543 \| ADCY1 \| Non Syndromic \| Autosomal Recessive \| Pakistan \| 8 \| \| 25528277 \| MYH7B, NOMO1, GRAPL, PDXDC1, FCGR2C, NBPF4, NPIP, FAM115A, OTOA, PLEKHN1, MEGF6, TMEM51, WLS, IRF2BP2, KIF26B, CNRIP1, MRPL35, SEMA4C, EIF5B, GCC2, GIGYF2, ACOXL, KCNJ13, GAL3ST2 \| Non Syndromic \|  \|  \| 150 cases, 157 controls \| \| 26549381 \| SLC26A4 \| Non Syndromic \| Autosomal Recessive \| China \| 22 \| \| 26497601 \| 12S rRNA (A1555G), COI/tRNASer(UCN) (G7444A) \| Non Syndromic \|  \| China \| 1 \| \| 26445815 \| SLC26A4, MYO15A, MYO7A, CDH23, PCDH15, USH2A, OTOA, ILDR1, TMC1, DFNB59, CABP2, TMPRSS3, GIPC3, LRTOMT, TECTA, ADGRV1, LHFPL5, MYO6, OTOF, PTPRQ, USH1C, CIB2, COL11A2, DFNB31, MARVELD2, POU3F4,, RDX, TMIE, ESPN, GPSM2, GRXCR1, KCNQ1, LOXHD1, MTRNR1, OTOGL, PDZD7, PRPS1, STRC, TRIOBP, WFS1 \| Non Syndromic \| Autosomal Recessive \| \|  \| \| 26426422 \| CIB2 (Arg186Trp) \| Non Syndromic \|  \| Caribbean, America \| \| \| 26416264 \| PDZD7 (854T>G, 1500C>A), CIB2 (223G>A), CDH23, TECTB \| Non Syndromic \| Autosomal Recessive \| Iran \| 201 probands \| \| 26409293 \| GJB2 (35delG), GJB6 \| Non Syndromic \| Autosomal Recessive \| Iran \| 300 \| \| 26399936 \| GJB2, SLC26A4, CDH23, MT-RNR1, MYO15A, OTOF, TMC1, TMPRSS3, TRIOBP, TMIE, DFNB59, WFS1, KCNQ4, COCH, TECTA \| Non Syndromic \| Both (specified in paper) \| Brazil \| 25 \| \| 26381000 \| GJB2 (35delG) \| Non Syndromic \| Autosomal Recessive \| Çukurova, Turkey \| \| \| 26371875 \| TBC1D24 \| Non Syndromic \| Autosomal Recessive \| Morocco \| 3 families \| \| 26331839 \| EYA4 \| Non Syndromic \| Autosomal Dominant \| Dutch family \| 23 \| \| 26282398 \| ATAD5, CDRT1, EPS8L2, NLRP6, OR51V1, TOLLIP \| Non Syndromic \| Autosomal Recessive \| Algeria \| 2 \| \| 26256111 \| TPRN, COCH \| Non Syndromic \| Autosomal Dominant \| South Korea \| 127 \| \| 26252218 \| *GJB2(109G>A(p.V37I), c.257C>G(p.T86R), c.187G>T(p.V63L), (c.235delC, c.176del16, c.299delAT), (c.512insAACG))*, *SLC26A4((c.1167G>A, c.1738_1739delAA, and c.1764_1765insAGGAAAATA), (c.IVS7-2A>G and c.IVS16-6G>A), (c.1079C>T (p.A360V), c.2168A>G (p.H723R), c.754T>C (p.S252P), c.1229C>T (p.T410M), c.1472T>C (p.I491T), c.1595G>T (p.S532I), and c.2007C>G (p.D669E)), (c.1790T>C (p.L597S) and c.147C>G (p.S49R), c.2283A>G (p.T761T) and c.1167G>A (p.G389G)*, *mtDNA12SrRNA(m.1555A>G)* \| Non Syndromic \|  \| China \| 155 \| \| 26226225 \| TMC1 (c.1810C>G (p.Arg604Gly) ) \| Non Syndromic \| Autosomal Recessive \| Morocco \| 1 sibling \| \| 26117665 \| GJB2 (p.Met34Thr and p.Arg184Pro) \| Non Syndromic \| Autosomal Recessive \| Portuguese \| 2 siblings \| \| 26100058 \| SLC26A4 (c.919-2A >G and p.H723R) \| Non Syndromic \| Autosomal Recessive \| China \| 150 cases, 3056 normal \| \| 26095810 \| GJB2 (c.235delC, c.109G>A) \| Non Syndromic \| Autosomal Recessive \| China \| 1481 (NSHL), 190 (GJB2 deafness patients) \| \| 26088551 \| GJB2 (Connexin 26) (R75Q, V37I) \| Non Syndromic \| Autosomal Recessive \| Korea \| 1 \| \| 26061264 \| GJB2 (p.R143W, p.V37I) \| Non Syndromic \| Autosomal Recessive \| Korea \| 588 \| \| 26059209 \| GJB2 (35delG, R32H, W24X) \| Non Syndromic \| Autosomal Recessive \| Ilam, Iran \| 62 \| \| 26043044 \| GJB2 (c.127G>T (p.V43L), c.293G>C (p.R98P), c. 107T>C (p.L36P) and c.187G>T (p.V63L) ) \| Non Syndromic \|  \| China \| 1067 \| \| 26036578 \| KCNQ4 (c.1044_1051del8) \| Non Syndromic \| Autosomal Dominant \| Tokyo, Japan \| 1 \| \| 26015337 \| EYA4 (c.1194delT ) \| Non Syndromic \| Autosomal Dominant \| Korea \| 14 probands \| \| 26011067 \| MYO7A (p.V1880E), TMC1 (p.L416R/p.A438T), PCDH15 (c.1238delT), MYO15A (c.9690+1G>A) \| Non Syndromic \|  \| China \| 6 families \| \| 25991456 \| SLC26A4 (c.1-103T>C, c. 1246A>C), OTOF (c.2485C>T), USH1C (c.238dupC), CDH23 (c.3929C>A, c.3929C>A), USH2A (c.2299delG, c.1724G>T, c.11928G>A, c.7475C>A, c.9203T>C, c.920_923dupGCCA, c.2299delG, c.3407G>A, c.2299delG), KCNQ1 (c.572_576delTGCGC), SOX10 (c.271_275delCCCGT, c.1127C>G), PAX3 (c.241G>T), NIPBL (c.5378T>G) \| Non Syndromic \|  \| African-American, Asian, Caucasian and Hispanic \| 71 \| \| 25963016 \| CDH23 (m.1555A > G and m.3243A > G ) \| Non Syndromic \| Autosomal Recessive \| National Tokyo Medical Center, Chiba Children’s Hospital, National Mie Hospital, National Center for Child Health and Development, Hyogo Prefectural Kobe Children’s Hospital, Keio University School of Medicine, National Hospital Organization Kanazawa Medical Center, Kanagawa Children’s Medical Center, National Hospital Organization Sendai Medical Center, Kanto Rosai Hospital, and Hiroshima Prefectural Hospital \| 621 \| \| 25961296 \| EYA4 (c.511G>C; p.G171R) \| Non Syndromic \| Autosomal Dominant \| China \| 57 \| \| 25941349 \| MET (c.2521T>G (p.F841V)) \| Non Syndromic \|  \| Pakistan \|  \| \| 25930172 \| PCDH15 (c.2367_2369delTGT) \| Non Syndromic and Usher syndrom \| \| China \| 1 family \| \| 25926005 \| EYA1 \| Non Syndromic \|  \| Brazil \| 11 \| \| 25919374 \| PTPRQ (c. 3125 A>G p.D1042G, c.5981 A>G p.E1994G) \| Non Syndromic \| Autosomal Recessive \| China \| 1572 \| \| 25830873 \| COCH, GJB2, PAX3 \| Non Syndromic \|  \| Seoul, Korea \| 6 \| \| 25829320 \| OTOGL (c.6467C>A, c.6474dupA) \| Non Syndromic \| Autosomal Recessive \| China \|  \| \| 25816005 \| HOMER2 \| Non Syndromic \| Autosomal Dominant \| Europe \| 19 \| \| 25809937 \| TECTA, EYA4 (c.1643C>G (p.T548R)), COL11A2 \| Non Syndromic \| Autosomal Recessive \| China \|  \| \| 25785835 \| PRPS1 (p.Ile275Thr and p.Gly306Glu) \| Non Syndromic \|  \| Spain \| 13 \| \| 25781927 \| EYA4 (p.S288X and p.Q393X), GRHL2 \| Non Syndromic \| Autosomal Dominant \| Korea \| 87 \| \| 25759012 \| OSBPL2, SLC17A9 \| Non Syndromic \| Autosomal Dominant \| Germany \| 2 \| \| 25719458 \| OTOGL, SERPINB6, GPR98, PDZ7 (AR), TECTA, MYH14 (AD) \| Non Syndromic \| Both \| Korea \| 11 \| \| 25708704 \| GJB2 (c.551G>C/c.397T>G (p.R184P/p.W133G)) \| Non Syndromic \|  \| Iran \| 3 \| \| 25666562 \| MARVELD2 \| Non Syndromic \| Autosomal Recessive \| Pakistan, Czech Roma \| 800 families \| \| 25636251 \| GJB2 (p.W24X or p.W77X ) \| Non Syndromic \|  \| Pakistan \| 84 families, 86 sporadic partcipants, 100 controls \| \| 25633957 \| COL11A2 (p.Ala37Ser) \| Non Syndromic \| Autosomal Recessive \| Tunisia, Turkey \| 2 families \| \| 25631766 \| PJVK \| Non Syndromic \| Autosomal Recessive \| China \| 65 families \| \| 25601850 \| DCDC2 \| Non Syndromic \| Autosomal Recessive \| Tunisia \| 1 family \| \| 25557914 \| PTPRQ \| Non Syndromic \| Autosomal Recessive \| China \| 1 family (9 members) \| \| 25555641 \| GJB2 (IVS1+1G>A) \| Non Syndromic \| Autosomal Recessive \| Iran \| 103 \| \| 25491636 \| GJB2 (c.71G>A (p.(Trp24*), c.231G>A (p.(Trp77*),c.71G>A (p.(Trp24*), c.231G>A (p.(Trp77*), c.71G>A (p.(Trp24*), c.35delG (p.(Gly12Valfs*2), c.35delG (p.(Gly12Valfs*2), c.71G>A (p.(Trp24*), c.71G>A (p.(Trp24*), CIB2 (c.272T>C (p.(Phe91Ser), HGF (c.482 +1986_8delTGA, c.482 +1986_8delTGA, c.482 +1986_8delTGA), SLC26A4 (c.1337A>G (p.(Gln446Arg), c.1337A>G (p.(Gln446Arg), c.1334T>G (p.(Leu445Trp), c.1337A>G (p.(Gln446Arg), c.716T>A (p.(Val239Asp), c.716T>A (p.(Val239Asp), c.691G>A (p.(Val231Met)), MYO7A (c.397dupC (p.(His133Profs*7), POU3F4 (3.1-Mb deletion), TMC1 (c.1114G>A (p.(Val372Met)), LHFPL5 (c.246delC (p.(Leu84fs*1)) \| Non Syndromic \| Autosomal Recessive \| Pakistan \| 10 families \| \| 25423259 \| TMC1 ( c.2030T>C) \| Non Syndromic \|  \| Iran \| 11 families \| \| 25182139 \| PRPS1 (c.343A>G (p.M115V) and c.925G>T (p.V309F) ) \| Non Syndromic \| X-linked \| Italy \| 17 families, 123 control \| \| 25012701 \| GJB2 (c.35delG and c.-23+1G>A) \| Non Syndromic \| Autosomal Recessive \| Iran \| 418 \| \| 24989646 \| GJB2 (c.109G>A, c.235delC), SLC26A4 (c.919A>G) \| Non Syndromic \| Autosomal Recessive \| China \| 5800 neonates \| \| 24890873 \| MYH9 (R705H) \| Non Syndromic \| Autosomal Dominant \| Helsinki \| 2 families (5 members) \| \| 24853665 \| STRC, CATSPER2, USH2A, TRIOBP (p.R861X and p.R920X), MYO15A, GPR98, TMPRSS3 (p.S99X) \| Non Syndromic \|  \| China \| 63 \| \| 24021014 \| mt-tRNA (C628T) \| Non Syndromic \| Mitochondrial \|  \|  \| \| 26885137 \| GJB2 (35delG, 176del16, 235delC, 299delAT), GjB3 (C538T), SLC26A4 (IVS7-2A>G, A2168G) and Mitochondrial 12S rRNA (A1555G, C1494T) \| Non syndromic \| Autosomal Recessive \| China \| 1 \| \| 26911058 \| GJB2, SLC26A4 \| Non Syndromic \|  \| Henan \| 100 \| \| 26911057 \| GJB2, SLC26A4 \| Non Syndromic \|  \| Guangxi Drovince \| 127 \| \| 26046157 \| GJB2, GJB6, GJA1 \| Non Syndromic \|  \| Cameroon and South Africa \| 205 \| \| 26121829 \| GJB2, GJB3, SLC26A4, WFS1 \| Non Syndromic \|  \| Zibo City, Shandong province \| 135 \| \| 29937786 \| POU3F4 \| Non Syndromic \| x-linked \| China \| 1 family \| \| 26499821 \| SLC26A4 \| Non Syndromic \|  \| China \| 60 \| \| 26911054 \| GJB2, SLC26A4 \| Non Syndromic \|  \| Inner Mongolia Autonomous regior \| 355 \| \| 27965898 \| *NLRP3* \| Syndromic (Muckle-Wells Syndrome) and Non Syndromic \| Autosomal Dominant \| China \| 1 family (10 members) \| \| 27941975 \| *POU3F4* \| Non Syndromic \| X-linked \| Poland \| 30, 10000 (all are cases) \| \| 27862068 \| *GJB2, GJB3, SLC26A4* \| Non Syndromic \|  \| China \| 25 \| \| 27827000 \| *GJB2 E1a (exone 1a)* \| Non Syndromic \| Autosomal Recessive \| Austria \| 30,11,9,6 (all are cases divided in different groups \| \| 27808407 \| *DIAPH1* \| Non Syndromic \| Autosomal Recessive \| China \| 2 families \| \| 27792752 \| *GJB2, SLC26A4, MT-RNR1* \| Non Syndromic \| Autosomal Recessive \| Northern China \| 695 \| \| 27771369 \| *SLC26A4* \| Syndromic (Pendred Syndrome) and Non Syndromic \| \| \|  \| \| 27759032 \| *MYO1A* \| Non Syndromic \| Autosomal Dominant \| North America \| 951 \| \| 27652356 \| *OTOF* \| Non Syndromic \|  \| Oman \| 10 \| \| 27631835 \| *CDH23* \| Syndromic (Usher syndrome) and Non Syndromic \| Autosomal Recessive \| US \| 7 cases, 35 control \| \| 27541164 \| *TBC1D24* \| Non Syndromic \| Recessive \| Pakistan \| 1 family \| \| 27535032 \| *POU4F3* \| Non Syndromic \| Autosomal Dominant \| China \| 1 family \| \| 27503514 \| *HOXA2* \| Non Syndromic \| Autosomal Dominant \| Italy \| 1 \| \| 27481527 \| *GJB2* \| Non Syndromic \| Both \| India \| 1 family \| \| 27469136 \| *STRC* \| Non Syndromic \| Autosomal Dominant \| Japan \| 194 \| \| 27397648 \| *MT-RNR1 (GJB2, TRMU)* \| Non Syndromic \|  \| China \| 200 cases, 120 healthy \| \| 27395428 \| *GJB2, GJB3, SLC26A4, MT-RNR1* \| Non Syndromic \| Autosomal Recessive and Mitochondrial \| South China \| 515 \| \| 27393652 \| *MYH9, MYH14* \| Non Syndromic \| Autosomal Dominant \| Korea \| 75 \| \| 27356075 \| *TGFA/TGFB3/MSX1* \| Non Syndromic \|  \| China \| 343 cases, 272 control \| \| 27344577 \| *ILDR1, OTOF, TMC1, MYO7A, GIPC3, TMPRSS3, TECTA, MYO15A, SLC26A4, MARVELD2, ESPN, PTPRQ, CDH23, MYO6, USH2A, POU3F4, SIX1, TRIOBP, EPS8, CIB2, PCDH15, LRTOMT, USH1G, TMIE, DFNB59, DFNB31, POU4F3* \| Non Syndromic \| Autosomal Recessive \| South Africa, Nigeria, Tunisia, Turkey, Iran, India, Guatemala, and the United States (South Florida) \| 342 \| \| 27260575 \| *CABP2, ILDR1, LHFPL5, LRTOMT, PJVK, TMIE, GJB2, CIB2* \| Non Syndromic \| Autosomal Recessive \| Czech, Europe \| 433 \| \| 27236922 \| *Tmc1* \| Non Syndromic \| Autosomal Dominant \| Lowa, US \| Mouse model \| \| 27169813 \| *GJB2* \| Non Syndromic \| Autosomal Recessive \| Morocco \| 152 families \| \| 27067584 \| *GJB2* \| Non Syndromic \| Autosomal Recessive \| Mauritania, Africa \| 139 \| \| 27066914 \| *GJB2, SLC26A4, mtDNA 12S rRNA* \| Non Syndromic \|  \| Tengzhou, China \| 156 \| \| 27063751 \| *MYO3A* \| Non Syndromic \| Autosomal Recessive \| China \| 1 family \| \| 27045574 \| *GJB2* \| Non Syndromic \| Autosomal Recessive \| Han Chinese origin \| 1 family as case, 200 control \| \| 26969326 \| *GJB2, STRC, SLC26A4, TECTA, MYO15A, MYO7A, USH2A, CDH23, ADCRV1, TMC1, PCDH15, OTOF, TMPRSS3, LOXHD1, OTOA, WFS1, COL11A2, KCNQ4, MYH14, MYO6, ACTG1, PTPRQ, MYH9, OTOGL, TRIOBP, CLDN14, COCH,ESPN,EYA4, LRTOMT, POU3F4, SMPX, TPRN, WHRN, ALMS1, DFNB59, DIABLO, DIAPH1, EYA1, GRXCR1, ILDR1, LHFPL5, MTRNR1, MYO1A, SLC17A8, SLC26A5, TSPEAR, USH1C, USH1G* \| Non Syndromic \| Autosomal Recessive \| USA \| out of 1119, 440 patients have been analyzed \| \| 27033575 \| GJB2, SLC26A4, mtDNA 12S rRNA \| Non Syndromic \|  \| Tianjin \| 225 \| \| 27018795 \| *GJB2*, *SLC26A4*, *CDH23*, *MYO15A*, DFNB59, 12rsRNA, PCDH15, OTOF, TRIOBP, TMC1, MYO1A, MYH14, MYO3A, EYA4, MYO6, MYO7A, TMPRSS3 (AR), MYH9, KCNQ4, TECTA, WFS1 (AD) \| nonsyndromic and syndromic \| Both \| China \| 382 \| \| 27023905 \| SLC22A4 \| Non Syndromic \| Autosomal Recessive \| Tunisia \| A large family \| \| 26900070 \| SLC26A4/pendrin \| Non Syndromic \| Autosomal Recessive \| Korea \| 31 \| \| 26896187 \| GJB2 (rs111033253), GJB6 \| Non Syndromic \| Autosomal Recessive \| Lithuania, Europe \| 158 \| \| 26841241 \| MYO3A \| Non Syndromic \| Autosomal Dominant \| \|  \| \| 26822030 \| TMC1 \| Non Syndromic \| Autosomal Recessive \| China \| 14 \| \| 26818607 \| OTOF \| Non Syndromic \| Autosomal Recessive \| China \| 37 \| \| 26810297 \| MYO15A \| Non Syndromic \| Autosomal Recessive \| China \| 2 generation family \| \| 26797701 \| SLC17A8 \| Non Syndromic \| Autosomal Dominant \| Korea \| 87 \| \| 26791358 \| MYO7A, USH1C, CDH23, PCDH15 \| Non Syndromic, Usher syndrome \| Autosomal Recessive \| Japan \| Among 1373, 227 selected \| \| 26778469 \| GJB2 \| Non Syndromic \| Autosomal Recessive \| Germany \| 506 \| \| 26749107 \| CX26 (p.E47K, p.E47Q, p.H100L, p.H100Y, p.R127L, and p.M195L) \| Both \|  \| Korea \|  \| \| 26746617 \| ACTG1, BSND, CCDC50, CDH23, CEACAM16, CIB2, CLDN14, COCH, COL11A2, CRYM, DFNA5, DFNB31, DFNB59, DIABLO, DIAPH1, ESPN, ESRRB, EYA4, GIPC3, GJB2, GJB3, GJB6, GPSM2, GRXCR1, HGF, ILDR1, KCNQ4, LHFPL5, LOXHD1, LRTOMT, MARVELD2, TSPEAR, USH1C, WFS1, MIR96, MSRB3, MYH14, MYH9, MYO15A, MYO3A, MYO1A, MYO6, MYO7A, OTOA, OTOF, OTOG, PCDH15, PNPT1, POU4F3, PTPRQ, RDX, SERPINB6, SLC17A8, SLC26A4, SLC26A5, STRC (rs12628603), TECTA, TJP2, TMC1, TMIE, TMPRSS3, TPRN, TRIOBP \| Non Syndromic \| Autosomal Recessive \| Korea \| 1 family \| \| 26634621 \| Sox2, Atoh1, Neurog1 (133A > G), and Neurod1 \| Non Syndromic \|  \| East Asia \| 1164 \| \| 27340645 \| GJB2 \| Non syndromic \| Autosomal Recessive \| Raipur, India \| 36 \| \| 29771057 \| GJB2, GJB6 \| Non Syndromic \|  \| China \| 318 \| \| 27247785 \| TMC1 \| Non Syndromic \| Autosomal Recessive \| Iran \| 100 \| \| 27635202 \| MYO15A \| Non Syndromic \| Autosomal Recessive \| Iran \| 30 families \| \| 29434063 \| *Cx26, SLC26A4, CLDN14, TMPRSS3, TMC1, TMIE, USH1C, OTOF* \| Non Syndromic \| Autosomal Recessive \| India \| 45 \| \| 29299381 \| *Mt DNA (m.7510T>C)* \| Non Syndromic \|  \|  \|  \| \| 29196752 \| *STRC, MYO15A, OTOF, TMC1, GJB2, MYO7A, USH2A* \| Non Syndromic \|  \| France \| 207 families \| \| 29174977 \| *mitochondrial MTRNR1* \| Non Syndromic \|  \|  \|  \| \| 29112224 \| *CIB2, IGDCC3, IGDCC4, ISLR, PEAK1* \| Non Syndromic \| Autosoamal Recessive \| Iran, Turkey, Europe \| 6 families \| \| 29106878 \| *GJB3* \| Non Syndromic \| Autosomal Dominant \| China \| 5700 \| \| 29088312 \| *TMEM132* \| Non Syndromic \| Autosoamal Recessive \| \|  \| \| 29048736 \| *PDZD7* \| Non Syndromic \| Autosoamal Recessive \| China \| 122 cases, 1751 control \| \| 28964305 \| *PJVK, MYO15A* \| Non Syndromic \| Autosoamal Recessive \| Morocco \| 3 families \| \| 28958982 \| *SYNE4* \| Non Syndromic \|  \| Turkey \| 2 \| \| 28946916 \| *TECTA* \| Non Syndromic \| Both \| Tokyo, Japan \| 1896 \| \| 28945813 \| *ILDR1* \| Non Syndromic \| Autosomal Recessive \| UAE \| 1 family \| \| 28934385 \| *ATP6V1B1(experimented in mice)* \| Non Syndromic \| Autosomal Recessive \| \|  \| \| 28821934 \| *GJB2* \| Non Syndromic \| Autosomal Recessive \| Austrian-Turkey \| 24 families \| \| 28802383 \| *KCNQ4* \| Non Syndromic \| Autosomal Dominant \| WB, India \| 10 families \| \| 28802351 \| *WFS1(Glu864Gly)* \| Non Syndromic \| Autosomal Dominant \| China \| 1 family \| \| 28790396 \| *POU4F3 (c.982A>G, p.Lys328Glu)* \| Non Syndromic \| Autosomal Dominant \| Taiwan \| 1 \| \| 28780564 \| *SLC26A4* \| Non Syndromic \|  \|  \|  \| \| 28734895 \| *GJB2, SLC26A4 or12S rRNA* \| Non Syndromic \|  \| Wuhan, China \| 101 \| \| 28733840 \| *COCH* \| Non Syndromic \| Autosomal Dominant \| Austria \| 1 family \| \| 28651654 \| *GJB2* \| Non Syndromic \|  \| New Zealand \| 60 \| \| 28647561 \| *SLC17A8* \| Hearing loss \|  \| Korea \| 1 family \| \| 28635225 \| *mitochondrial 12S rRNA (1555A>G or 1494C>T )* \| Non Syndromic \|  \|  \|  \| \| 28601886 \| *AUNA1* \| Non Syndromic \| Autosomal Dominant \| Germany \| 1 family \| \| 28579530 \| *GJB2, mitochondrial tRNAPhe gene (593T>C)* \| Non Syndromic \|  \| China \| 1 family \| \| 28505178 \| *GJB2, GJB3, SLC26A4, MT-RNR1* \| Non Syndromic \|  \| China \| 1 family, 342 control \| \| 28501645 \| *GJB6, CDH23, MYO6, TMC1, OTOF, OTOA* \| Non Syndromic \| Autosomal Recessive \| Qatar \| 80 \| \| 28483220 \| *GJB2* \| Non Syndromic \| Autosomal Recessive \| Iran \| 16 \| \| 28472130 \| *MYO7A* \| Non Syndromic \| Autosomal Recessive \| Morocco \| 2 families \| \| 28428247 \| *Cx26 (N14K, D50N, N54K, M163V, and S183F)* \| Syndromic and Non Syndromic \| Autosomal Dominant \| \|  \| \| 28405014 \| *GJB2* \| Non Syndromic \| Autosomal Recessive \| Russia \| 2569 \| \| 28383030 \| *SLC26A4, GJB2, MYO15A, CDH23* \| Non Syndromic \|  \| Korea \| 28 \| \| 28340560 \| *KCNQ4* \| Non Syndromic \| Autosomal Dominant \| China \| 531 \| \| 28335750 \| *GJB2, GJB3, 12S rRNA, SLC26A4, OTOF* \| Non Syndromic \| Autosomal Recessive (OTOF) \| Guangxi Zhuang Autonomous Region \| 127 \| \| 28263784 \| *TMPRSS3, GJB2* \| Non Syndromic \| Autosomal Recessive \| USA \| 2 \| \| 28173822 \| *PDZD7, COL1A1* \| Non Syndromic \| Autosomal Recessive (PDZD7) and Autosomal Dominant (COL1A1) \| Pakistan \| 4 \| \| 28102197 \| *GJB2 (DFNA3)* \| Non Syndromic \| Autosomal Dominant \| China \| 1 family \| \| 28013291 \| *SLC44A4* \| Non Syndromic \| Autosomal Dominant \| China \| 1 family \| \| 28012541 \| *TECTA* \| Non Syndromic \| Autosomal Recessive \| Iran \| 25 families \| \| 28012540 \| *GJB2, GJB6* \| Non Syndromic \| Autosomal Recessive \| Syria \| 70 families \| \| 28713423 \| *ILDR1* c.942C > A (p.Cys314Ter) \| Non syndromic \| Autosomal Recessive \| Europe \| 2 \| \| 28077706 \| Cx26 (studied in mouse) \| Non syndromic \|  \|  \|  \| \| 29072634 \| GJB2, SLC26A4, TMPRSS3 \| Non Syndromic \| Autosomal Recessive \| Korea \| 88 \| \| 28785060 \| ILDR1, ILDR2 (chicken cochlea) \| Non Syndromic \|  \|  \|  \| \| 28383030 \| GJB2, SLC26A4 \| Non Syndromic \|  \| Korea \| 28 \| \| 30473554 \| *GJB2* \| Non Syndromic \| Autosomal Recessive \| China \| 1 family \| \| 30419932 \| *IARS2* \| Non Syndromic \| Autosomal Recessive \| Iran \| 2 families \| \| 30406641 \| *MARVELD2 (DFNB49)* \| Non Syndromic \|  \| China \| 283 \| \| 30335006 \| *mtDNA(case report)* \| Non Syndromic \|  \| Taiwan \| 1 \| \| 30244537 \| *PNPT1* \| Non Syndromic \| Autosomal Recessive \| Canada \| 3 \| \| 30180840 \| *NEDD4, NEFH, TTN, MON1B, ZAN, SIRPA, ADC, GPR98, LRBA, PAX2, DNAH2, RBPJ, GRM7, SIK3, WFS1* \| Non Syndromic \|  \| London, UK and South Carolina, US \| 20 cases, 10 cases \| \| 30176854 \| *POU3F4* \| Non Syndromic \| X-linked recessive \| China \| 100 \| \| 30175721 \| *MYO6* \| Non Syndromic \| Autosomal Dominant \| China \| 1 family \| \| 30173967 \| *mtDNA (A1555G, A3243G, A7445G, 7472insC)* \| Non Syndromic \|  \|  \|  \| \| 30094485 \| *GJB2* \| Non Syndromic \|  \| Hungary \| 239 cases, 169 controls \| \| 30077349 \| *SLC26A4* \| Non Syndromic \| Autosomal Recessive \| Iran \| 40 \| \| 30068307 \| *GJB2, GJB6, SLC26A4, MTRNR1, MTTS1, MYO15A* \| Non Syndromic \| Autosomal Recessive \| China \| 5 \| \| 30055731 \| *GJB2* \| Non Syndromic \| Autosomal Recessive \| Turkey \| 31 cases, 31 controls \| \| 30047143 \| *HOMER2* \| Non Syndromic \| Autosomal Dominant \| China \| 1 family \| \| 30029624 \| *PCDH15, USH1G* \| Non Syndromic and Usher syndrom \| Autosomal Recessive \| Pakistan \| 4 \| \| 29986705 \| ABHD12, BSND, CLRN1, DIABLO, FTO, HSD17B4, MARVELD2, MYO3A, P2RX2, PTPN11, SLC52A3, TMIE, ACTB, ACTG1, ADGRV1, AIFM1, ALMS1, ANKH, AP1S1, ATP1A3, ATP6V1B1, BCAP31, BCS1L, BRAF, ADCY1, ATP2B2, ATP6V1B2, BDP1, CABP2, CACNA1D, CCDC50, CDH23, CEACAM16, CHD7, CIB2, CISD2, CLCNKA, CLCNKB, CLDN14, CLPP, COL4A6, COL9A2, COL9A3, COQ6, DCAF17, DDX11, DFNA5, DFNB59, CRYM, DCDC2, DIAPH3, DSPP, DIAPH1, DNMT1, ECHS1, EDN3, EDNRB, EPS8L2, ESPN, ESRRB, EYA1, EYA4, FGF3, FGFR3, ELMOD3, EPS8, FAM65B, FBLN1, GATA3, GIPC3, GJB2, GJB3, GJB6, GPSM2, GRHL2, GRXCR1, HARS2, HGF, HOXA1, HOXB1, FGFR1, FGFR2, FOXI1, GRXCR2, ILDR1, KARS, KCNE1, KCNJ10, KCNQ1, KCNQ4, LARS2, LHFPL5, LHX3, LOXHD1, LRP2, LRTOMT, GTF2IRD1, HMX2, HMX3, HOMER2, MASP1, MIR96, MITF, MSRB3, MT-CO1, MT-RNR1, MT-TH, MT-TK, MT-TL1, MT-TS1, MYH9, MYH14, KITLG, MAF, MARS2, MCM2, MYO6, MYO7A, MYO15A, NARS2, NDP, NLRP3, OPA1, OSBPL2, OTOA, OTOF, OTOG, OTOGL, MT-CO3, MT-TA, MT-TE, MT-TS2, PAX3, PCDH15, PDZD7, PEX1, PEX2, PEX3, PEX6, PEX5, PEX26, POU3F4, POU4F3, PRPS1, NDUFA13, NFIX, PNPT1, SEMA3E, PTPRQ, RAF1, RDX, RMND1, SERAC1, SERPINB6, SLC52A2, SIX5, SLC4A11, SLC9A1, SLC26A5, SLITRK6, SMPX, SNAI2, SOX10, SPATA5, STRC, SYNE4, TBC1D24, TECTA, TIMM8A, TJP2, TMC1, TK2, TMEM132E, TMPRSS5, TNC, TMPRSS3, TPRN, TRIOBP, TSPEAR, USH1C, USH1G, USH2A, WFS1, WHRN, XYLT2, TP63 \| \| \| Spain \| 50 \| \| 29955957 \| *GREB1L* \| Non Syndromic \|  \| US \|  \| \| 29926981 \| *GJB2, GJB3, GJB6* \| Non Syndromic \| Autosomal Recessive \| China \| 100 \| \| 29880844 \| *MYO3A* \| Non Syndromic \| Autosomal Dominant \| Brazil \| 36 \| \| 29860631 \| *PDE1C, GJB2, GJB3, SLC26A4* \| Non Syndromic \| Autosomal Dominant \| China \| 5 generation chinease family \| \| 29796015 \| *PDZD7 (mouse)* \| Syndromic and Non Syndromic \| \|  \|  \| \| 29776397 \| *S1PR2* \| Non Syndromic \| Autosomal Recessive \| Iran \| 3 cases, 7 normal \| \| 29703829 \| *CEACAM16* \| Non Syndromic \| Autosomal Recessive \| Iran \| 2 families \| \| 29634755 \| *GJB2, SLC26A4, MTRNR1* \| Non Syndromic \|  \| China \| 142417 neonates \| \| 29605370 \| *GIPC3* \| Non Syndromic \| Autosomal Recessive \| Iran \| 6 cases, 50 healthy \| \| 29605365 \| *GJB2, SLC26A4, mtDNA 12S rRNA* \| Non Syndromic \| Both \| Mainland China \| 299 \| \| 29551497 \| *SLITRK6* \| Non Syndromic \| Autosomal Recessive \| Morocco \| 1 family \| \| 29533536 \| *TMC1* \| Non Syndromic \| Autosomal Recessive \| China \| 110 \| \| 29501320 \| *SLC26A4* \| Non Syndromic \| Autosomal Recessive \| China \| 1 \| \| 29501291 \| *GJB2* \| Non Syndromic \| Autosomal Recessive \| Iran \| 70 \| \| 29472286 \| *CAPN5* \| Non Syndromic \|  \|  \| 1 \| \| 29453195 \| *IFNLR1* \| Non Syndromic \| Autosomal Dominant \| China \| 3 generation family \| \| 29447821 \| *CLDN14* \| Non Syndromic \| Autosomal Recessive \| China \| 500 \| \| 29408584 \| *MtDNA* \| Non Syndromic \|  \| China \| 97 cases, 376 control \| \| 29400105 \| *MYO7A* \| Non Syndromic \| Autosomal Dominant \| China \| 15 cases, 151 controls \| \| 29348176 \| *mitochondrial 12S rRNA, tRNA (4317A→G)* \| Non Syndromic \|  \|  \|  \| \| 29320412 \| *GJB2, SLC26A4* \| Non Syndromic (GJB2, SLC26A4), Syndromic (SLC26A4, Pendred syndrome) \| Autosomal Recessive \| Austria \| 19 \| \| 29293505 \| *CHD7, HDAC8, MITF, NEFL, RYR1 (recessive and Dominant), SF3B4, TECTA, MIR96, TMC1, MYH14, WFS1, GJB2, OTOF, SLC26A4, TMPRSS3, USH2A* \| Non Syndromic \|  \| Slovenia, BiH \| 56 \| \| 29287889 \| *EYA4, PAX3* \| Non Syndromic \|  \|  \| 2 \| \| 29287849 \| *CDH23* \| Non Syndromic \| Autosomal Recessive \| China \| 1 \| \| 29266521 \| *DFNA5* \| Non Syndromic \| Autosomal Dominant \| Europe, East Asia, Iran \| 5 families \| \| 30575790 \| miR-183/96/182 (Clic5, Rdx, Ezr, Rac1, Myo1c, Pvrl3 and Sox2) (tested in mice) \| Non syndromic \|  \|  \|  \| \| 29541197 \| SLC26A4 \| Non syndromic \|  \| Anhui, China \| 1 \| \| 29501291 \| GJB2 \| Non Syndromic \| Autosomal Recessive \| Iran \| 70 \| \| 29511501 \| MYO7A, EYA1 \| Non Syndromic \|  \| Iran \| 2 families \| \| 29308629 \| PTPRQ, MYO1A \| Non Syndromic \| Autosomal Recessive \| Iran \| 1 case, 200 control \| \| 30155266 \| EYA4 \| Non Syndromic \| Autosomal Dominant \| Japan \| 1 \| \| 31231018 \| *MYH14* \| Non Syndromic \|  \| Canada \| 3 generation family \| \| 31176026 \| *TMC1* \| Non Syndromic \|  \| Iran \| 1 family \| \| 31163360 \| *GHRL2, SLC26A4, GJB2, TECTA, CLDN14, EYA4* \| Non Syndromic \|  \| Caucasians and African America \| 430 \| \| 31129248 \| *PDZD7* \| Non Syndromic \|  \| China \| 1 family \| \| 31028865 \| *KCNQ4* \| Non Syndromic \|  \| Pakistan \| 4 \| \| 30953472 \| *MYO15A* \| Non Syndromic \|  \| China \| 15 \| \| 30935366 \| *TECTA* \| Non Syndromic \|  \| Korea \| 4 \| \| 30762455 \| *GJB2, SLC26A4, MT-RNR1* \| Non Syndromic \| Autosomal Dominant, Autosomal Recessive, Mitochondrial \| China \| 90 \| \| 30760222 \| *LOXHD1* \| Non Syndromic \| Autosomal Recessive \| China \| 1 \| \| 30730013 \| *GJB2, LRTOMT* \| Non Syndromic \| Autosomal Recessive \| Egypt \| 103 cases, 100 control \| \| 30693673 \| *GJB2, SLC26A4, mtDNA 12s rRNA* \| Non Syndromic \|  \| Shanxi Province, China \| 1201 cases, 300 control \| \| 30682115 \| *ACTG1 (AD), CCDC50 (AD), CDH23 (AR), CLDN14 (AR), COCH, COL11A, CRYM, DIAPH1, DSPP, GSDME (AD), ESPN, ESRRB (AR), EYA4, GJB6 (BOTH), GRHL2, KCNQ4 (AD), LHFPL5, MARVELD2 (AR), MYH14, MYH9 (AD), MYO15A, MYO3A (AR), MYO6, MYO7A (BOTH), OTOA, PCDH15, PJVK (AR), POU3F4 (X- linked), RDX (AD), SLC26A5, STRC, TECTA (AR), TMC1, TMIE (BOTH), TMPRSS3, TRIOBP, USH1C, WFS1 (AR), WHRN* \| Non Syndromic \|  \| Taiwan \| 41 cases, 40 control \| \| 30335140 \| *TBC1D24(studied in mice)* \| Non Syndromic \|  \| Italy \|  \| \| 30205178 \| *TRMU* \| Non Syndromic \| Autosomal Recessive \| Iran \| 4 cases \| \| 31301639 \| MYO15A (R3191C) \| Non Syndromic \| Autosomal Recessive \| Kazakh, China \| 1 family \| \| 31370293 \| GJB2 (c.427C>T, c.35delG, c.645delT, 154G>T p.(Val52Phe), c.94C>A (p.Arg32Ser)), MYO15A (c.3757-2A>G, c.843C>A) \| Non Syndromic \|  \| Peruvian Population, South America \| 133 \| \| 31852093 \| GJB2 (c.113T>C, c.235delC) \| Non Syndromic \| Autosomal Recessive \| China \| 3 \| \| 31095577 \| OTOF, DIAPH1, KCNQ4, GJB3, GJB6, MYH14, DFNA5, WFS1, TECTA, COCH, EYA4, MYO7A, COL11A2, POU4F3, MYH9, ACTG1, MYO6, SLC17A8, GRHL2, HGF, ILDR1, CIB2, TMC1, CRYM, CCDC50, MYO1A, MIRN96, TJP2, DIABLO, CEACAM16, GJB2, MYO15A, SLC26A4, TMIE, TMPRSS3, OTOF, CDH23, GIPC3, STRC, USH1C, OTOA, PCDH15, RDX, GRXCR1, TRIOBP, CLDN14, MYO3A, WHRN, ESRRB, ESPN, MARVELD2, DFNB59, SLC26A5, LRTOMT, LHFPL5, PNPT1, MSRB3, PRPS1, POU3F4, SMPX, COL4A6, LOXHD1, TPRN, GPSM2, PTPRQ, KARS, SERPINB6, CABP2, USH2A \| Non Syndromic \| Autosomal Recessive \| Japan \| 2265 \| \| 29752989 \| MARVELD2 (c.1555delinsAA) \| Non Syndromic \| Autosomal Recessive \| Iran \| 20 families \| \| 31846914 \| GJB2, GJB3, GJB6 \| Non syndromic \| Autosomal Recessive \| Sivas, Turkey \| 53 \| \| 31579092 \| MYO15A (c.9611_9612+8delTGGTGAGCAT) \| Non syndromic \| Autosomal Recessive \| Iran \| 1 \| \| 31781163 \| *CLDN14* \| Non syndromic \|  \| Yemen \| 3 families \| \| 31700827 \| *GJB2* \| Non syndromic \| Autosomal Recessive \| Iran \| 80 \| \| 31846914 \| GJB2, GJB3, and GJB6 \| Non syndromic \|  \| Sivas, Turkey \| 53 \| \| 30973918 \| GJB2, SLC26A4 \| Non syndromic \|  \| China \| 220 \| \| 30741891 \| SLC26A4 \| Non syndromic \|  \|  \| 103 \| \| 30476627 \| LHFPL5 \| Non syndromic \| Autosomal Recessive \| Oman \| 1 family \| \| 31731535 \| GJB2, GJB6 \| Non Syndromic \| Autosomal Recessive \| Cameroon \| 93 \| \| 31472357 \| GJB2 \| Non Syndromic \|  \|  \| 1 \| \| 31445392 \| CDH23 \| Non Syndromic \|  \| China \| 1 family \| \| 31231791 \| TRRAP \| Non Syndromic \| Autosomal Dominant \| China \| 4 \| \| 31554319 \| TECTA \| Non Syndromic \| Autosomal Dominant \| Japan \| 812 \| \| 31655630 \| PTPRQ \| Non Syndromic \| Autosomal Dominant \| Germany \| 14 \| \| 31547530 \| LOXHD1 \| Non Syndromic \| Autosomal Recessive \| Japan \| 8074 \| \| 32860223 \| GJB2, MYO15A, LOXHD1, TMPRSS3, CDH23, OTOG, OTOF, AIFM1, CABP2, DIAPH1, PTPRQ, RDX, SLC26A4, TBC1D24, TECTA, TMC1 \| Non Syndromic \| Autosomal Recessive \| Czechia \| 421 \| \| 32776944 \| THOC1 \| Non Syndromic \| Autosomal Dominant \| China \| 15 \| \| 30651814 \| GJB2, GJB3, SLC26A4 \| Non Syndromic \| Autosomal Recessive \| Linyi, China \| 116 \| \| 30989077 \| GJB2 \| Non Syndromic \|  \| Iran \| 21 \| \| 31162818 \| GJB2, GJB6 \| Non Syndromic \| Autosomal Dominant \| Iran \| 131 \| \| 30804975 \| GJB2, GJB6, MYO7A, MYO15A, FOXI1, KCNJ10, SLC26A4, TMIE, TMC1, TMPRSS3, OTOF, CDH23, ATP2B2, GIPC3, STRC, OTOG, USH1C, TECTA, OTOA, PCDH15, RDX, GRXCR1, TRIOBP, CLDN14, MYO3A, WHRN, ESRRB, ESPN, MYO6, GJA1, HGF, ILDR1, MARVELD2, DFNB59, SLC26A5, LRTOMT, LHFPL5, BSND, MSRB3, LOXHD1, TPRN, GPSM2, PTPRQ, SERPINB6, GJB3 \| Non Syndromic \| Autosomal Recessive \| China \| 1 \| \| 30804975 \| ACTG1, CCDC50, CEACAM16, COCH, CRYM, DFNA5, DIABLO, DIAPH1, DSPP, EYA4, GJB2, GJB3, GJB6, GRHL2, KCNQ4, MIR96, MYH14, MYH9, MYO1A, MYO6, MYO7A, POU4F3, SIX1, SLC17A8, TECTA, TJP2, TMC1, WFS1, DIAPH \| Non Syndromic \| Autosomal Dominant \| China \| 1 \| \| 30804975 \| PRPS1, POU3F4, SMPX \|  \| X-linked \| China \| 1 \| \| 31053783 \| GJB2, TMPRSS3, MYO15A, OTOF, STRC \| Non Syndromic \| Autosomal Recessive \| Africans/African-America \| 68 \| \| 31527525 \| OTOA \| Non Syndromic \| Autosomal Recessive \| Japan \| 2262 \| \| 30518198 \| GJB2 \| Non Syndromic \| Autosomal Recessive \| Kurdish population, Iran \| 45 families \| \| 30942159 \| EYA4 \| Non Syndromic \| Autosomal Dominant \| China \| 1 family \| \| 31434872 \| kKCNQ4 \| Non Syndromic \| Autosomal Dominant \| Korea \| 397 \| \| 31801140 \| MET \| Non Syndromic \| Autosomal Recessive \| Morocco \| 1 \| \| 31322239 \| CDH23 \| Non Syndromic \| Autosomal Recessive \| China \| 1 family \| \| 31454969 \| PDZD7 \| Non Syndromic \| Autosomal Recessive \| Korea \| 2 \| \| 31432506 \| PLS1 \| Non Syndromic \| Autosomal Dominant \| Turkey \| 1 family \| \| 30733538 \| MYO15A, GJB2, MYO7A, SLC26A4, TMC1, ESPN, POU3F4, MYH14, EYA1 \| Non Syndromic \|  \| Vietnam \| 87 \| \| 30991779 \| *GJB2,GJB3,SLC26A4, mtDNA* \| Non Syndromic \|  \| China \| 492 \| \| 32066420 \| MYO6 \| Non Syndromic \| Sensorineural (AR) \| Australia \| 1 family \| \| 31898538 \| MYO15A, MITF \| Non syndromic \| Autosomal Recessive \| America \| 2 \| \| 32143290 \| *MYO6* \| Non syndromic \| Autosomal Dominant \| Japan \| 8074 families \| \| 32115674 \| CDH23 \| Non syndromic/Syndromic \| \| Oman \| 1 family \| \| 32087478 \| DIAPH1 \| Non syndromic \| Autosomal Dominant \| China \| 13 members (1 family) \| \| 31995783 \| KCNQ4 \| Non syndromic \| Autosomal Dominant \| China \|  \| \| 31906439 \| *CDC14A* \| Non syndromic \| Autosomal Recessive \| Iran & Pakistan \| 12 families \| \| 31875531 \| OTOF \| Non Syndromic \| Autosomal Recessive \| Jammu & Kashmir, India \| 1 \| \| 31706454 \| BSND, ACTG1, CDH23 \| Non Syndromic \|  \|  \| 27 \| \| 32517708 \| LRTOMT \| Non Syndromic \| Autosomal Recessive \| Iran \| 1 family \| \| 32107407 \| EYA4 \| Non Syndromic \| Autosomal Dominant \| Germany \| 98 \| \| 30837189 \| GJB2, GJB6 \| Non Syndromic \|  \| Brazil \| 53 \| \| 31669356 \| GJB2 \| Non Syndromic \| Autosomal Recessive \| Iran \| 100 \| \| 32115674 \| CDH23 \| Both \|  \| Oman \| 1 family \| \| 32623615 \| MYO15A \| Non Syndromic \| Autosomal Recessive \| Iran \| 1 \| \| 32390314 \| POU4F3 \| Non Syndromic \| Autosomal Dominant \| China \|  \| \| 32682410 \| GIPC3, LOXHD1, TMPRSS3, TECTA, MYO15A, DFNB59, TRIOBP \| Non Syndromic \|  \| Pakistan \| 5 families \| \| 32487028 \| TRIOBP \| Non Syndromic \| Autosomal Recessive \| China \| 1 \| \| 32658972 \| SLC12A2 \| Non Syndromic \|  \|  \|  \| \| 32382995 \| KCNQ4, TECTA, WFS1 \| Non Syndromic \|  \| America, Japan \| 921 (Europe/America), 162 (Japan) \| \| 32251972 \| SLC26A4 \| Both \|  \| Naples, Italy \| 1 \| \| 32649979 \| HOXA2 \| Non Syndromic \| Both \| China \| 2 families \| \| 32742378 \| CRYM \| Non Syndromic \| Autosomal Dominant \| China \| 1 family \| \| 23767834 \| MYO6, TECTA, POU4F3, COCH \| Non Syndromic \| Autosomal Dominant \| China \| 190 \| \| 23767834 \| MTTS1 \| Non Syndromic \| Mitochondrial \| China \| 190 \| \| 28221712 \| MYH14 \| Non Syndromic \| Autosomal Dominant \| Korea \| 2 \| \| 26849169 \| PDZD7 \| Non Syndromic \| Autosomal Recessive \| \| 2 \| \| 28576516 \| SLC26A4 \| Non Syndromic \| Autosomal Recessive \| China \| 3 \| \| 31200317 \| GJB2 \| Non Syndromic \|  \| Algeria \| 91 \| \| 28225033 \| GJB2, GJB3, SLC26A4, MT-RNR1, MT-TS1 \| Non Syndromic \|  \| China \| 12 \| \| 28640090 \| GJB2, SLC26A4, 12s rRNA \| Non Syndromic \|  \| China \| 71 \| \| 25788563 \| CDH23, SLC26A4, MYO15A, COL11A2, MYO7A, OTOF \| Non Syndromic \| Autosomal Dominant \| Japan \| 1120 cases, 269 controls \| \| 29982980 \| MPZL2 \| Non Syndromic \| Autosomal Recessive \| Turkey, Iran \| 2 families \| \| 30582396 \| SLC26A4, MYO6, PJVK, CDH23 \| Non Syndromic \| Autosomal Recessive \| Iran \| 100 \| \| 29255404 \| CIB2 \| Non Syndromic \|  \|  \|  \| \| 29151245 \| USH2A \| Non Syndromic \| Autosomal Dominant \| \| 2 \| \| 30344259 \| SERPINB6, TMIE, ESPN (AR), COCH, ACTG1, KCNQ4, GJB3 (AD) \| Non Syndromic \| Both \| Moravia-Silesia \| 200 \| \| 27693694 \| TPRN \| Non Syndromic \| Autosomal Recessive \| \|  \| \| 26188157 \| GJB2, GJB6, SLC26A4 \| Non Syndromic \| Autosomal Recessive \| India \| 215 \| \| 29575629 \| GJB2 \| Non Syndromic \| Autosomal Dominant \| \| 3 \| \| 26036852 \| TMPRSS3 \| Non Syndromic \| Autosomal Recessive \| Caucasian \| 5 \| \| 25792669 \| LOXHD1 \| Non Syndromic \|  \| Japan \| 1314 \| \| 31389194 \| SLC26A4, KCNQ4, MYO7A, MYO15A, TMPRSS3, ESPN, TMC1, GIPC3, LHFPL5, WFS1, DFNB59, GRXCR1, ESRRB, LRTOMT \| Non Syndromic \|  \| Pakistan \| 40 \| \| 30872718 \| WFS1, DIAPH1, SLC26A4, MYO3A, CDH23, PCDH15, USH1C, TRIOBP, MYO15A, GJB2, GJB3, MYO7A \| Non Syndromic \|  \|  \|  \| \| 29741433 \| DSPP \| Non Syndromic \| Autosomal Dominant \| China \| 8 \| \| 28367085 \| GJC3 \| Non Syndromic \|  \|  \|  \| \| 28900455 \| GJB2, GJB4 \| Non Syndromic \| Autosomal Recessive \| Iran \| 2 \| \| 28990112 \| SLC26A4 \| Non Syndromic \|  \| China \| 52 \| \| 29559740 \| GJB2, SLC26A4, mtDNA 12s rRNA \| Non Syndromic \| X-linked \| China \| 59 \| \| 25992148 \| GJB2 \| Non Syndromic \| Autosomal Recessive \| \| 67 cases, 5 controls \| \| 29595809 \| GJB2, STRC, LOXHD1, MYO15A, USH2A, SOX10, CDH23, CEACAM16, COCH, TMC1, COL2A1, \| Non Syndromic \|  \|  \| 80 \| \| 31552524 \| STRC \| Non Syndromic \| Autosomal Recessive \| Prague, Czech \| 11 \| \| 28677207 \| LRP5 \| Non Syndromic \| Autosomal Recessive \| China \| 29 \| \| 26079994 \| TMC1 \| Non Syndromic \| Autosomal Dominant \| China \| 5315 \| \| 31160754 \| GJB2 \| Non Syndromic \| Autosomal Recessive \| \|  \| \| 28051029 \| POU3F4 \| Non Syndromic \| X-linked recessive \| China \| 1 \| \| 30589569 \| GJB2, SLC26A4, GJB3, mt-RNA1 \| Non Syndromic \|  \| China \| 130 \| \| 27081546 \| KCNQ4, IMMP2L, DOCK4 \| Non Syndromic \| Autosomal Dominant \| Brazil \| 132 \| \| 31661684 \| CABP2 \| Non Syndromic \| Autosomal Recessive \| Iran \| 2 \| \| 30826590 \| MYO7A \| Non Syndromic \|  \|  \|  \| \| 25792667 \| MYO15A \| Non Syndromic \| Autosomal Recessive \| Japan \| 1120 \| \| 30816908 \| GJB2 \| Non Syndromic \| Autosomal Recessive \| China \| 1 \| \| 30579064 \| MYO15A \| Non Syndromic \| Autosomal Recessive \| Iran \| 24 families \| \| 27999687 \| POU4F3 \| Non Syndromic \| Autosomal Dominant \| China \| 4 generation family \| \| 26783197 \| GJB2, SLC26A4, mtDNA 12s rRNA \| Non Syndromic \|  \| China \| 117 \| \| 21811972 \| GJB2, MARVELD2, HGF, TRIOBP, USH1C, GRXCR1, LHFPL5, TMC1, NF2, DFNA5, GPSM2, MYO7A, GJB6, POLR1D, DIAPH3 \| Non Syndromic \|  \| China \| 52 \| \| 25255398 \| USH1G \| Non Syndromic \| Autosomal Recessive \| Duach \|  \| \| 29773520 \| GJB2 \| Non Syndromic \|  \| Brazil \| 100 \| \| 31250571 \| MYO15A, OTOF, RDX \| Non Syndromic \|  \| China \| 131 \| \| 29016196 \| GJB2 \| Non Syndromic \| Autosomal Recessive \| UAE \| 50 cases, 120 control \| \| 29287879 \| SMPX \| Non Syndromic \| x-linked \| North America \| 3 generation family \| \| 25077649 \| OSBPL2 \| Non Syndromic \| Autosomal Dominant \| China \| 7 generation family \| \| 31107121 \| GJB2, SLC26A4, mtDNA12SrRNA \| Non Syndromic \|  \| China \| 314 \| \| 30758234 \| STRC \| Non Syndromic \| Autosomal Recessive \| UAE \| 109 cases, 50 control \| \| 30245514 \| COL11A1 \| Non Syndromic \| Autosomal Dominant \| Europe \| 48 \| \| 29590114 \| PPIP5K2 \| Non Syndromic \| Autosomal Recessive \| Pakistan \| 2 families \| \| 26264712 \| CDH23 \| Non Syndromic \| Autosomal Recessive \| Korea \| 438 \| \| 27657680 \| DMXL2 \| Non Syndromic \| Autosomal Dominant \| China \| 21 \| \| 23936043 \| TNC \| Non Syndromic \| Autosomal Dominant \| China \| 70 \| \| 28281779 \| GJB2, SLC26A4, LHFPL5, USH2A, ESPN, MYO7A, LRTOMT, PCDH15, USH2A, EPS8L2 \| Non Syndromic \|  \| Pakistan \| 12 families \| \| 31397523 \| PLS1 \| Non Syndromic \| Autosomal Dominant \| Europe \| 3 families \| \| 26075227 \| GJB2, GJB6 \| Non Syndromic \| Autosomal Recessive \| \|  \| \| 31175426 \| CLDN9 \| Non Syndromic \| Autosomal Recessive \| Turkey \|  \| \| 26873147 \| mt-DNA, GJB2 \| Non Syndromic \|  \|  \| 178 \| \| 27068579 \| TPRN, CLDN14, DFNB59, ESPN, ESRRB, GIPC3, GPSM2, GRXCR1, HGF, ILDR1, LHFPL5, LOXHD1, LRTOMT, MARVELD2, MSRB3, MTAP, MYO3A, MYO15A, CABP2, CACNA1D, OTOA, OTOF, PTPRQ, RDX, SERPINB6, SLC26A5, TMPRSS3, TRIOBP, TMIE, STRC, SLC26A4, FOXI1, KCNJ10, CDH23, PCDH15, DFNB31, USH1C, MYO7A (AR), COL11A2, GJB2, GJB3, GJB6, MYO6, TECTA, TMC1 (AR & AD), ACTG1, CCDC50, COCH, CRYM, DFNA5, DIABLO, DIAPH1, EYA4, GRHL2, KCNQ4, MIR96, MYH4, MYO1A, POU4F3, SLC17A8, TJP2, WFS1, MYH9, DIAPH3 (AD), POU3F4, PRPS1, SMPX (x-LINKED) \| Non Syndromic \|  \| Germany \| 131 \| \| 30368370 \| SLC26A4 \| Non Syndromic \| Autosomal Recessive \| China \| 15 families \| \| 25333454 \| GJB4 \| Non Syndromic \|  \| China \| 253 \| \| 27063752 \| GJB2 \| Non Syndromic \|  \| China \| 300 cases, 484 controls \| \| 28271504 \| WFS1, GJB2 \| Non Syndromic \| Autosomal Dominant \| Japan \| 74 \| \| 32149082 \| LOXHD1 \| Non Syndromic \| Autosomal Recessive \| China \| 4 families, 200 controls \| \| 31273342 \| ABCC1 \| Non Syndromic \| Autosomal Dominant \| China \| 28 \| \| 29692870 \| MYO15A, MYO7A, TMC1, PCDH15 \| Non Syndromic \|  \| Uyghur \| 12 families \| \| 25149764 \| GJB2, mt-RNR1, SLC26A4 \| Non Syndromic \|  \|  \| 141 \| \| 27530448 \| mt-RNR1, MT-TS1 \| Non Syndromic \|  \| India \| 729 \| \| 24599119 \| SLC26A4 \| Non Syndromic \|  \| Japan \| 1511 \| \| 20206386 \| TMIE \| Non Syndromic \|  \| China \| 250 cases, 120 control \| \| 26968074 \| MYO7A \| Non Syndromic \| Autosomal Recessive \| China \| 120 \| \| 22617145 \| GJB3, GJB6 \| Non Syndromic \| Autosomal Recessive \| Korea \| 215 \| \| 30068397 \| SLC26A4 \| Non Syndromic \|  \| Brazil \| 88 \| \| 24586623 \| TECTA \| Non Syndromic \| Autosomal Dominant \| China \| 3187 \| \| 22289209 \| SLC26A4, FOXI1 \| Non Syndromic \|  \| China \| 21 \| \| 27621663 \| OTOF \| Non Syndromic \| Autosomal Recessive \| Ashkenazi Jewish \| \| \| 31035178 \| GJB2, GJB3, SLC26A4, mt-DNA, 12SRNA \| Non Syndromic \|  \| China \| 506 \| \| 29849560 \| MYO15A \| Non Syndromic \| Autosomal Recessive \| China \| 4 \| \| 25761933 \| GJB2, SLC26A4, mtDNA \| Non Syndromic \|  \| China \| 484 \| \| 23714752 \| COL4A6 \| Non Syndromic \| X-linked \| Hungary \| 3 generation family \| \| 25393658 \| GJB2 \| Non Syndromic \| Autosomal Recessive \| India \| 1 family \| \| 30174017 \| SMPX \| Non Syndromic \| X-linked \| China \| 60 \| \| 31215297 \| GJB2, GJB6 \| Non Syndromic \|  \| Iran \| 400 \| \| 23826813 \| GJB2, mt12srRNA \| Non Syndromic \|  \| China \| 658 cases, 462 controls \| \| 32562050 \| COCH \| Non Syndromic \| Autosomal Recessive \| Pakistan, Europe,Middle Easter, Unknown \| \| \| 19657183 \| CX29 \| Non Syndromic \|  \| Taiwan \| 253 \| \| 27886419 \| PRPS1 \| Non Syndromic \| Autosomal Recessive \| Korea \| 42 \| \| 24729539 \| TBC1D24 \| Non Syndromic \| Autosomal Dominant \| Europe \| four generation family \| \| 24781754 \| CLIC5 \| Non Syndromic \| Autosomal Recessive \| Turkey \| 213 \| \| 30123251 \| CDH23, EYA4, LOXHD1, MYO7A \| Non Syndromic \|  \| China \| 10 families \| \| 19299023 \| MYO7A \| Non Syndromic \|  \| Taiwan \| 231 cases, 100 controls \| \| 20100600 \| 12srRNA \| Non Syndromic \|  \| China \| 1642 \| \| 28053790 \| POU4F3 \| Non Syndromic \| Autosomal Dominant \| China \| 16 \| \| 26308726 \| MYO15A \| Non Syndromic \| Autosomal Recessive \| China \| four generation family \| \| 28541280 \| GJB2, SLC26A4 \| Non Syndromic \| Autosomal Recessive \| China \| 80 \| \| 27082237 \| RAI1, SLC26A4, OTOF \| Non Syndromic \|  \| South Siberia \| 163 \| \| 28901477 \| mt-DNA 12srRNA, GJB2, SLC26A4 \| Non Syndromic \|  \| China \| 283 \| \| 32235586 \| TMPRSS3 \| Non Syndromic \| Autosomal Recessive \| Taiwan \| 230 cases, 120 controls \| \| 25802247 \| GRXCR1 \| Non Syndromic \| Both \| Japan \| 1120 \| \| 20602916 \| TJP2 \| Non Syndromic \|  \| Israel \| 58 \| \| 28075205 \| MYO7A, MYO6, KCTD3, NUMA1, MYH9, KCNQ1, UBC, DIAPH1, PSMC2, RDX \| Non Syndromic \|  \| sub-Saharan Africa \| 82 cases, 250 controls \| \| 22796198 \| SLC26A4 \| Non Syndromic \|  \| China \| 195 \| \| 25008054 \| TECTA \| Non Syndromic \| Autosomal Dominant \| Mongolia, China \|  \| \| 29484430 \| MITF \| Non Syndromic \| Autosomal Dominant \| China \| 26 \| \| 28542515 \| SMPX \| Non Syndromic \| X-linked \| China \| 338 cases, 295 controls \| \| 29710868 \| PANX1 \| Non Syndromic \|  \|  \|  \| \| 31997689 \| MYO15A, MYO7A \| Non Syndromic \| Autosomal Recessive \| Iran \| 100 \| \| 23834103 \| GJB2, SLC26A4, mtDNA 12S rRNA \| Non Syndromic \|  \| Tibetan, Tu nationality, and Mongolian probands from the northwest of China \| 189 \| \| 24660976 \| mt-RNR1 \| Non Syndromic \|  \| India \| 36 \| \| 26279247 \| PCDH15, MT-TS1 \| Non Syndromic \| Autosomal Dominant \| China \| 11 \| \| 20947814 \| TECTA \| Non Syndromic \| Autosomal Dominant \| Korea \| 62 \| \| 32728090 \| MITF \| Non Syndromic \| Autosomal Recessive \| USA \| 130 \| \| 25388789 \| COCH, ACTG1, TMC1, POU4F3 \| Non Syndromic \| Autosomal Dominant \| China \| 23 \| \| 22154049 \| GJB2, SLC26A4, GJB3, 12s rRNA \| Non Syndromic \|  \| China \| 179 \| \| 27247933 \| GJB2, SLC26A4, mtDNA12SrRNA \| Non Syndromic \|  \| China \| 339 \| \| 23717403 \| KCNQ4 \| Non Syndromic \| Autosomal Dominant \| Japan \| 287 \| \| 21660509 \| GIPC3 \| Non Syndromic \|  \| Pakistan \|  \| \| 19876648 \| GJC3 \| Non Syndromic \|  \|  \|  \| \| 31419744 \| GJB2 \| Non Syndromic \| Autosomal Recessive \| Iran \| 5 \| \| 27014650 \| TRIOBP \| Non Syndromic \| Autosomal Recessive \| Iran \| 1 \| \| 26668150 \| TJP2, GJB2 \| Non Syndromic \| Autosomal Dominant \| China \| 686 \| \| 20130915 \| GJA1 \| Non Syndromic \|  \|  \|  \| \| 25788564 \| PTPRQ \| Non Syndromic \|  \| Japan \| 220 \| \| 19814620 \| GJB2, GJB3, GJB6, WFS1 \| Non Syndromic \|  \| Croatia \| 58 \| \| 31393079 \| AARS, ABHD12, AIFM1, DNMT1, FIG4, GBE1, GJB1, GJB3, GLA, DNAJB2, INF2, KIF5A, MFN2, MPZ, MYH14, NDRG1, NEFL, PDK3, PEX12, PEX7, PHYH, PMP22, POLG, PRPS1, SBF2, SCN9A, SETX, SH3TC2, SLC5A7, SLC25A46, SOX10, SPTLC1, SURF1, TRPV4, TTR, TYMP \| Non Syndromic \|  \| French \| 3412 \| \| 24206587 \| MYO15A \| Non Syndromic \| Autosomal Recessive \| China \| 56 cases, 108 controls \| \| 21392827 \| GJB2, GJB6 \| Non Syndromic \|  \| South Africa \| 182 \| \| 22172221 \| GJB2 \| Non Syndromic \| Autosomal Recessive \| Iran \| 50 families \| \| 33288323 \| GJB2 \| Non Syndromic \|  \| Taiwan \| 1517 \| \| 33205915 \| TMC1, CDH23 \| Non Syndromic \| Autosomal Recessive \| Iran \| 4 generation family \| \| 31347505 \| SLC26A4 \| Non Syndromic \|  \| China \| 634 cases, 220 controls \| \| 28003573 \| DIAPH1 \| Non Syndromic \| Autosomal Dominant \| Korea \| 1 \| \| 21731760 \| GJB2, GJB6 \| Non Syndromic \|  \|  \|  \| \| 26440088 \| ILDR1 \| Non Syndromic \| Autosomal Recessive \| Iran \| 2 families \| \| 31103816 \| MYO6 \| Non Syndromic \| Autosomal Dominant \| \|  \| \| 33062705 \| LOXHD1 \| Non Syndromic \| Autosomal Recessive \| China \| 4 families \| \| 28099493 \| COCH \| Non Syndromic \| Autosomal Dominant \| China \| 2 families \| \| 31827252 \| HARS2 \| Non Syndromic \| Autosomal Recessive \| Europe \| 3 \| \| 26196677 \| MCM2 \| Non Syndromic \| Autosomal Dominant \| China \| 76 cases, 145 normal \| \| 29876232 \| mitochondrial 12S rRNA and tRNASer(UCN) \| Non Syndromic \|  \| China \| 1 family \| \| 23901193 \| mt-RNR1 \| Non Syndromic \|  \| Brazil \| 78 \| \| 25162826 \| GJB2 \| Non Syndromic \|  \| Cameroon, South Africa \| 205 \| \| 25451287 \| GJB2 \| Non Syndromic \|  \| China \|  \| \| 20170898 \| TPRN \| Non Syndromic \| Autosomal Recessive \| Morocco \| 1 family \| \| 24814232 \| OTOF \| Non Syndromic \| Autosomal Recessive \| Korea \| 71 \| \| 24053799 \| OTOF \| Non Syndromic \| Autosomal Recessive \| Japan \| 160 cases, 192 normal \| \| 24785695 \| GJB6, GJA1 \| Non Syndromic \|  \| Cameroon, South Africa \| \| \| 19426954 \| KCNJ10, SLC26A4 \| Non Syndromic \| Autosomal Recessive \| US \| 2 families \| \| 33269433 \| ESRRB, CDH23, TMIE, CABP2 \| Non Syndromic \|  \| Pakistan \| 5 families \| \| 30943474 \| MYO15A \| Non Syndromic \| Autosomal Recessive \| Iran \| 3 \| \| 25807530 \| NARS2 \| Non Syndromic \| Autosomal Recessive \| \| 1 family \| \| 22241583 \| mt-DNA \| Non Syndromic \|  \|  \|  \| \| 20170899 \| TPRN \| Non Syndromic \| Autosomal Recessive \| Pakistan \| 1 family \| \| 23504403 \| GJB2 \| Non Syndromic \|  \| Pakistan \| 70 \| \| 19101659 \| GJB2 \| Non Syndromic \| Autosomal Recessive \| France \| 1 \| \| 23510777 \| GIPC3 \| Non Syndromic \| Autosomal Recessive \| Saudi Arab \| 5 \| \| 27176802 \| SLC26A4, GJB3 \| Non Syndromic \| Autosomal Dominant \| China \| 1 family \| \| 28198501 \| GJB2 \| Non Syndromic \| Autosomal Recessive \| China \| 118 cases, 242 normal \| \| 24341454 \| GJB2, SLC26A4, mt-DNA 12srRNA \| Non Syndromic \|  \| China \| 235 \| \| 32485727 \| CDH23 \| Non Syndromic \| Autosomal Recessive \| Iran \| 3 \| \| 27466889 \| GJB2, GJB3, GJB6, KCNQ4, MYO7A, MYO15A, mt-RNR1, mt-TS1, SLC26A4, SLC26A5, TMC1 \| Non Syndromic \|  \| Newzealand \| 64 \| \| 25788562 \| LRTOMT \| Non Syndromic \| Autosomal Recessive \| Japan \| 106 \| \| 29207085 \| GJB2 \| Non Syndromic \|  \| China \|  \| \| 23865914 \| MYO15A \| Non Syndromic \| Autosomal Recessive \| Korea \| 13 \| \| 19125024 \| GJB2 \| Non Syndromic \|  \| Brazil \| 300 \| \| 19744334 \| GJB2, SLC26A4 \| Non Syndromic \|  \| China \| 284 \| \| 28273078 \| GJB2, GJB6, SLC26A4, mt-RNR1, mt-TS1 \| Non Syndromic \|  \| Europe \| 136 \| \| 26096904 \| GJB2, GJB6 \| Non Syndromic \|  \| Eastern Sicily \| 196 \| \| 21122151 \| GJB2 \| Non Syndromic \|  \| China \| 212 \| \| 21368133 \| CEACAM16 \| Non Syndromic \| Autosomal Dominant \| \|  \| \| 29620237 \| ACTG1 \| Non Syndromic \| Autosomal Dominant \| Korea \| 1 family \| \| 24526180 \| TMPRSS3 \| Non Syndromic \| Autosomal Recessive \| Korea \| 51 \| \| 22610276 \| COCH \| Non Syndromic \| Autosomal Dominant \| Korea \| 1 family \| \| 22567367 \| GJB2 \| Non Syndromic \| Autosomal Recessive \| \| 201 \| \| 24875298 \| GJB2 \| Non Syndromic \|  \| Germany \| 30 cases, 9 normal \| \| 29258540 \| WFS1 \| Non Syndromic \| Autosomal Dominant \| Korea \| 1 family \| \| 21689626 \| CDH23 \| Non Syndromic \|  \|  \|  \| \| 25331638 \| TMEM132E \| Non Syndromic \| Autosomal Recessive \| China \| 1 family \| \| 25227905 \| MYO6 \| Non Syndromic \| Autosomal Dominant \| China \| 1 family \| \| 28583500 \| GJB2. SLC26A4, 12srRNA \| Non Syndromic \|  \| China \| 380 \| \| 23506231 \| ACTG1 \| Non Syndromic \| Autosomal Dominant \| Korea \| 6 \| \| 19309289 \| MYO15A \| Non Syndromic \| Autosomal Recessive \| Tunis \| 77 \| \| 25239229 \| SLC26A4 \| Non Syndromic \| Autosomal Recessive \| Iran \| 4 families \| \| 24616153 \| MYO1A \| Non Syndromic \| Autosomal Dominant \| Germany \| 109 \| \| 25390158 \| SLC26A4 \| Non Syndromic \| Autosomal Recessive \| Iran \| 165 \| \| 22068689 \| mt-rRNA \| Non Syndromic \|  \|  \|  \| \| 30740825 \| OTOA \| Non Syndromic \|  \| Korea \|  \| \| 25153233 \| GJB2 \| Non Syndromic \| Autosomal Dominant \| Jewish Ashkenazi \| 4 generation family \| \| 27562378 \| MITF, SOX10, CHD7, PTPN11, KMT2D \| Non Syndromic \| Autosomal Recessive \| Turkey, Iran, USA \| 102 \| \| 26173970 \| CIB2 \| Non Syndromic \|  \| Pakistan \|  \| \| 29568747 \| TRIOBP, LHFPL5, CDH23, PCDH15, MYO7A \| Non Syndromic \| Autosomal Recessive \| Iran \| 5 families \| \| 23669344 \| SERPINB6 \| Non Syndromic \| Autosomal Recessive \| \|  \| \| 32519820 \| MYO3A \| Non Syndromic \| Autosomal Dominant \| Germany \| 3 generation family \| \| 23434199 \| GJB2, GJb6 \| Non Syndromic \| Autosomal Recessive \| Tunis \| 95 \| \| 19888295 \| OTOF, PJVK, TMHS, SLC26A4, CDH23, MYO7A, TECTA, OTOA, MYO15A, TMPRSS3 \| Non Syndromic \|  \| Palestinian origin \| 286 families \| \| 19818876 \| 12srRNA \| Non Syndromic \|  \| China \| 1742 \| \| 24194196 \| MYO7A \| Non Syndromic \| Autosomal Recessive \| Arab \| 2 families \| \| 21046548 \| COCH \| Non Syndromic \| Autosomal Dominant \| America \| 4 generation family \| \| 23076972 \| POU3F4 \| Non Syndromic \| x-linked \| Korea \| 6 families \| \| 21255762 \| ILDR1 \| Non Syndromic \| Autosomal Recessive \| Pakistan \|  \| \| 31972369 \| SCD5 \| Non Syndromic \| Autosomal Dominant \| China \| 1 family \| \| 23690975 \| TMC1 \| Non Syndromic \| Autosomal Recessive \| China \| 50 \| \| 23213405 \| Taperin \| Non Syndromic \| Autosomal Recessive \| \|  \| \| 31116475 \| KARS \| Non Syndromic \|  \|  \|  \| \| 24612839 \| GJB2, GJB3, SLC26A4, WFS1 \| Non Syndromic \|  \| China \| 701 \| \| 32770655 \| SLC26A4 \| Non Syndromic \|  \| China \| 2 cases, 500 controls \| \| 27375115 \| MYO15A \| Non Syndromic \| Autosomal Recessive \| Indonesia \|  \| \| 24387994 \| TBC1D24, CACNA1H, IGFALS, PRSS27, SRRM2, THOC6 \| Non Syndromic \|  \|  \|  \| \| 22363784 \| GIPC3 \| Non Syndromic \| Autosomal Recessive \| Turkey \| 3 \| \| 21250555 \| TMC1 \| Non Syndromic \| Autosomal Recessive \| Iran \| 2 families \| \| 32486382 \| GSDME \| Non Syndromic \| Autosomal Dominant \| Europe \| 1 family \| \| 29148562 \| CDH23 \| Non Syndromic \| Autosomal Recessive \| India \| 116 \| \| 26752218 \| SLC26A4 \| Non Syndromic \|  \| Brazil \| 58 \| \| 19393408 \| GJB2 \| Non Syndromic \|  \|  \| 38 \| \| 21388256 \| GJB2 \| Non Syndromic \| Autosomal Recessive \| Iran \| 114 \| \| 19376484 \| 12srRNA \| Non Syndromic \|  \| China \| 1 family \| \| 22617256 \| PJVK \| Non Syndromic \| Autosomal Recessive \| Pakistan \|  \| \| 24816743 \| TECTA \| Non Syndromic \| Autosomal Dominant \| Korea \| 21 \| \| 21633365 \| POU3F4 \| Non Syndromic \| x-linked \| Pakistan \|  \| \| 25328248 \| CLDN14, MYO3A, ESRRB, ESPN, MYO6, HGF, ILDR1, MARVELD2, COL11A2, PJVK, SLC26A5, LRTOMT, LHFPL5, MSRB3, LOXHD1, TPRN, PTPRQ, SERPINB6, GJB3, SANS, USH2A, VLGR1, USH3A, PDZD7 \| Non Syndromic \| Autosomal Recessive \| \|  \| \| 22736430 \| MYO15A \| Non Syndromic \| Autosomal Recessive \| Iran \| 140 \| \| 19929408 \| GJB2 \| Non Syndromic \|  \| Portugal \| 3 families \| \| 20563649 \| GJB2, GJB6 \| Non Syndromic \|  \| Brazil \| 77 \| \| 20593197 \| GJB2, GJB3, GJB4, GJC3 \| Non Syndromic \|  \| Taiwan \| 253 \| \| 32617096 \| MYO15A \| Non Syndromic \| Autosomal Recessive \| China \| 1 family \| \| 22906306 \| OTOF \| Non Syndromic \| Autosomal Recessive \| Iran \| 38 families \| \| 19645626 \| MYH9 \| Non Syndromic \| Autosomal Dominant \| Japan \| 157 \| \| 24256046 \| GJB2 \| Non Syndromic \|  \| China \| 2398 \| \| 21031134 \| MYO7A \| Non Syndromic \| Autosomal Recessive \| Tunis \| 2 \| \| 19715472 \| GJB2 \| Non Syndromic \| Autosomal Recessive \| Iran-Turkey \| 202 families \| \| 22288896 \| TMC1 \| Non Syndromic \| Autosomal Recessive \| North Africa, the Middle East, and parts of South Asia \| 476 \| \| 23606368 \| POU3F4 \| Non Syndromic \|  \| Israel \|  \| \| 24551843 \| GJB2, GJB6 \| Non Syndromic \| Autosomal Recessive \| Arab \| 1 \| \| 21185009 \| MSRB3 \| Non Syndromic \| Autosomal Recessive \| Pakistan \| 2 \| \| 32987832 \| TBC1D24 \| Non Syndromic \| Both \| Pakistan \|  \| \| 19603065 \| CACNA1B, EDF1, PTGDS, EHMT1, QSOX2, NOTCH1, MIR126, MIR60 \| Non Syndromic \| Autosomal Recessive \| Pakistan \|  \| \| 31150550 \| GJB2, GJB3, GJB6 \| Non Syndromic \| Autosomal Recessive \| India \| 25 families \| \| 21117948 \| GJB2, MYO15A, TMIE, TMC1, OTOF, CDH23, MYO7A, SLC26A4, PCDH15, LRTOMT, SERPINB6, TMPRSS3 \| Non Syndromic \| Autosomal Recessive \| Turkey \| 49 families \| \| 25288386 \| GJB2, GJB6 \| Non Syndromic \| Autosomal Recessive \| Mexico \| 78 \| \| 20096468 \| GJB2 \| Non Syndromic \|  \| North-West Romania \| 75 \| \| 22389666 \| GJB2, SLC26A4, GJB6, POU3F4, mtDNA 12S rRNA \| Non Syndromic \|  \| Tibetan \| 114 \| \| 21868108 \| GJB2 \| Non Syndromic \| Autosomal Recessive \| China \| 2 \| \| 27230773 \| MT-RNR1, MT-TK \| Non Syndromic \|  \| Brazil \| 152 cases, 104 normal \| \| 31427568 \| OSBPL2 \| Non Syndromic \| Autosomal Dominant \| \|  \| \| 31854501 \| TMC1 \| Non Syndromic \| Autosomal Recessive \| Saudi \| 366 families \| \| 21722859 \| SMAC/DIABLO \| Non Syndromic \| Autosomal Dominant \| China \| 6 generation family \| \| 19692489 \| SLC26A4 \| Non Syndromic \|  \|  \|  \| \| 22578326 \| GPSM2 \| Non Syndromic \| Autosomal Recessive \| US and Canada \| 8 families \| \| 31016883 \| TMPRSS3 \| Non Syndromic \| Autosomal Recessive \| China \| 300 \| \| 23256547 \| 12srRNA, GJB2, GJB3, SLC26A4 \| Non Syndromic \|  \| China \|  \| \| 21078986 \| COCH, EYA4, OTOF, GJB2, STRC, MYO6, MYO7A, PCDH15, USH2A, KCNQ4, WFS1, MYH14, CDH23 \| Non Syndromic \| Both \| USA \|  \| \| 30896630 \| SLC26A4, GJB2, MYO15A, TRIOBP, PTPRQ, MYO7A, mt-RNR1, OTOG, TMC1, WFS1, GJB3, ESPN, MYO6, DIAPH3, DSPP, DIAPH1, MITF, CDH23, PCDH15, OTOF, KCNQ4, USH2A, EDN3, KCNQ1 \| Non Syndromic \|  \| China \| 58 \| \| 20642360 \| MYO15A \| Non Syndromic \| Autosomal Recessive \| Turkey \| 104 families \| \| 30446579 \| PEX26 \| Non Syndromic \| Autosomal Recessive \| \| 1 \| \| 29713870 \| ELMOD3 \| Non Syndromic \| Autosomal Dominant \| China \| 5 generation chinease family \| \| 29607572 \| MYO6 \| Non Syndromic \| Autosomal Dominant \| Korea \| 81 families \| \| 19939467 \| GJB2 \| Non Syndromic \|  \|  \| 318 \| \| 27867666 \| PCDH15 \| Non Syndromic \|  \|  \|  \| \| 19204907 \| SLC26A4 \| Non Syndromic \|  \| Africa-America \| 47 \| \| 28695016 \| TMPRSS3 \| Non Syndromic \| Autosomal Recessive \| China \| 150 families \| \| 26748055 \| CDH23 \| Non Syndromic \| Autosomal Recessive \| \|  \| \| 19274735 \| MYO15A \| Non Syndromic \| Autosomal Recessive \| Iran \| 2 families \| \| 25262649 \| CDH23, CLDN14, CLRN1, COL11A2, DFNB31, DFNB59, ESPN, ESRRB, GIPC3, GJB2, GJB3, GJB6, GPR98, GPSM2, GRHL2, GRXCR1, HGF, ILDR1, LHFPL5, LOXHD1, LRTOMT, MARVELD2, MYO15A, MYO3A, MYO6, MYO7A, OTOA, OTOF, PCDH15, RDX, SLC26A4, SLC26A5, STRC, TECTA, TMC1, TMIE, TMPRSS3, TPRN, TRIOBP, USH1C, USH1G, USH2A (AR); ACTG1, COCH, COL11A2, CRYM, DFNA5, DIAPH1, DSPP, GJB2, GJB3, GJB6, KCNQ4, MYH14, MYH9, MYO1A, MYO6, MYO7A, POU4F3, TECTA, TMC1, WFS1 (AD) \| Non Syndromic \| Both \|  \|  \| \| 30123247 \| MYO3A \| Non Syndromic \|  \|  \|  \| \| 26552864 \| mt-TFB1 \| Non Syndromic \|  \|  \|  \| \| 31858762 \| USH1C \| Non Syndromic \| Autosomal Dominant \| Korea \| 48 \| \| 20021999 \| PRPS1 \| Non Syndromic \| X-linked \| China \| 106 cases, 29 normal \| \| 22103400 \| GJB2, GJB6 \| Non Syndromic \|  \| Qatar \| 126 \| \| 21704276 \| SLC26A4 \| Non Syndromic \| Autosomal Recessive \| US \| 86 \| \| 19576567 \| HGF \| Non Syndromic \| Autosomal Recessive \| India and Pakistan \| 40 families \| \| 23638949 \| GJB2, SLC26A4, mt-DNA \| Non Syndromic \|  \| China \| 227 cases, 200 normal \| \| 22037723 \| GJB2 \| Non Syndromic \|  \| Bulgaria \| 51 \| \| 33095980 \| GJB2, OTOF, USH1C, MYO15A, TBC1D24, OTOA, TMC1, EDNRB, SALL1, USH1G, TWNK, CDH23 \| Non Syndromic \|  \| China \| 21 families \| \| 21816241 \| LHFPL5 \| Non Syndromic \| Autosomal Recessive \| Tunis \| 129 \| \| 31198993 \| TOP2B \| Non Syndromic \| Autosomal Dominant \| China \| 6 \| \| 30874365 \| POU3F4 \| Non Syndromic \| X-linked \|  \|  \| \| 23554706 \| GJB2, GJB3, GJB6, SLC26A4, SLC26A5, 12SrRNA, tRNA \| Non Syndromic \|  \| China \| 135 \| \| 31247458 \| CDH23 \| Non Syndromic \|  \|  \|  \| \| 32802042 \| TMC1, MYO15A \| Non Syndromic \| Autosomal Recessive \| China \| 2 families \| \| 32048449 \| OTOF, CDH23, PCDH15, ADGRV1, PDZD7, KARS, OTOG, GRXCR2, MYO6, GRHL2, POU3F4, ACTG1, ALX3, BSND, CABP2, CCDC50, CIB2, CLDN14, CLPP, CLRN1, COCH, COL11A1, COL11A2, COL2A1, COL4A3, COL4A4, COL4A5, COL4A6, COL9A1, COL9A2, COMT2, CRYM, DFNA5, DFNB31, DFNB59, DIABLO, DIAPH1, DIAPH3, DNAJC17, DSPP, ECM1, EDN3, EDNRB, ELMOD3, ESPN, ESRRB, EYA1, EYA4, FGF3, FGF8, FGFR1, FGFR3, FLNA, FOXI1, FREM1, GATA3, GIPC3, GJB2, GJB3, GJB6, GPSM2, GRXCR1, HARS, HARS2, HGF, HMX1, HOXA2, HSD17B4, IL13, ILDR1, KARS, KCNE1, KCNJ10, KCNQ1, KCNQ4, KITLG, KRT9, LAMA3, LARS2, LHFPL5, LRTOMT, GRXCR2, MARVELD2, MIr-96, MIr-182, MIr-183, MITF, MSRB3, MYH14, MYH9, MYO15A, MYO1A, MYO3A, MYO6, NDP, NF2, OTOA, P2RX2, PABPN1, PAX3, PCDH15, PNPT1, POLR1C, POLR1D, POU3F4, POU4F3, PROK2, PROKR2, PRPS1, PTPRQ, RDX, RPGR, SALL1, SALL4, SEC23A, SEMA3E, SERPINB6, SIX1, SIX5, SLC17A8, SLC19A2, SLC26A5, SMAD4, SMPX, SNAI2, SOX10, STRC, TBC1D24, TECTA, THOC1, TIMM8A, TJP2, TMC1, TMIE, TMPRSS3, TNC, TRIOBP, TRMU, TSPEAR, USH1C, USH1G, USH2A, WFS1, WHRN \| Non Syndromic \|  \| China \| 12 families \| \| 22852811 \| mt-RNR1 \| Non Syndromic \|  \| India \| 3030 cases, 200 control \| \| 32864763 \| GJB2; GJB6; MYO7A; MYO15A; FOXI1; KCNJ10; SCL26A4; TMIE; TMC1; TMPRSS3; OTOF; CDH23; ATP2B2; GIPC3; STRC; OTOG; USH1C; TECTA; OTOA; PCDH15; RDX; GRXCR1; TRIOBP; CLDN14; MYO3A; WHRN; ESRRB; ESPN; MYO6; GJA1; HGF; ILDR1; MARVELD2; DFNB59; SLC26A5; LRTOMT; LHFPL5; BSND; MSRB3; LOXHD1; TPRN; GPSM2; PTPRQ; SERPINB6; GJB3 (AR), ACTG1; CCDC50; CEACAM16; COCH; CRYM; DFNA5; DIABLO; DIAPH1; DSPP; EYA4; GJB2; GJB3; GJB6; GRHL2; KCNQ4; MIR96; MYH14; MYH9; MYO1A; MYO6; MYO7A; POU4F3; SIX1; SLC17A8; TECTA; TJP2; TMC1; WFS1; DIAPH3 (AD); PRPS1; POU3F4; SMPX (X-linked); MT-RNR1; MT-TS1 \| Non Syndromic \| Autosomal Recessive \| Iran \|  \| \| 30173967 \| mt-DNA \| Non Syndromic \|  \| Syria \| 50 families \| \| 24645897 \| GJB2 \| Non Syndromic \|  \| China \| 107 cases, 61 normal \| \| 26561413 \| CDH23 CLDN14 COL11A2 DFNB31/WHRN DFNB59/PJVK ESPN ESRRB GIPC3 GJB2 GJB3 GJB6 GPSM2 GRXCR1 HGF ILDR1 KARS LHFPL5 LOXHD1 LRTOMT/COMT2 MARVELD2 MSRB3 MYO15A MYO3A MYO6 MYO7A OTOA OTOF PCDH15 PTPRQ RDX SERPINB SLC26A4 SLC26A5 TBC1D24 TECTA TMC1 TMIE TMPRSS3 TPRN TRIOBP USH1C (AR); ACTG1 CCDC50 CEACAM COCH DFNA5 DIAPH1 EYA4 GJB2 GJB3 GJB6 GRHL2 KCNQ4 MIR96 MYH14 MYH9 MYO1A MYO6 MYO7A POU4F3 SIX1 SLC17A8 SMAC/DIABLO TBC1D24 TECTA TJP2 TMC1 WFS1 (AD); POU3F4 PRPS1 SMPX (x-linked) \| Non Syndromic \| Autosomal Recessive \| Turkey \| 21 \| \| 20373850 \| TMC1 \| Non Syndromic \| Autosomal Recessive \| Algeria, Iran, Iraq, Lebanon, Pakistan, Tunisia, and Turkey \| 11 \| \| 25792666 \| POU3F4 \| Non Syndromic \| X-linked \| Japan \| 194 \| \| 19371219 \| GJB2, GJB6 \| Non Syndromic \|  \| Italy \| 734 \| \| 26482070 \| GJB2 \| Non Syndromic \|  \| Portuguese \| 502 \| \| 22285650 \| SLC26A4, FOXI1 \| Non Syndromic \|  \| Italy \| 19 \| \| 24913888 \| GJB3 \| Non Syndromic \|  \|  \|  \| \| 32711451 \| MYH14 \| Non Syndromic \| Autosomal Dominant \| China \| 4 generation family \| \| 19270079 \| CDH23 \| Non Syndromic \|  \|  \|  \| \| 20583176 \| GJB2 \| Non Syndromic \| Autosomal Dominant \| \| 3 \| \| 19107147 \| PCDH15 \| Non Syndromic \| Autosomal Recessive \| island of Newfoundland \| 1 family \| \| 24729547 \| TBC1D24 \| Non Syndromic \| Autosomal Dominant \| \| 17 \| \| 25589040 \| CEACAM16 \| Non Syndromic \| Autosomal Dominant \| China \| 5 generation chinease family \| \| 31586237 \| GJB2 \| Non Syndromic \|  \|  \|  \| \| 19941053 \| GJB2 \| Non Syndromic \|  \| Turkey \| 151 \| \| 32567228 \| WFS1 \| Non Syndromic \|  \| China \| 384 \| \| 29048421 \| OTOF \| Non Syndromic \|  \| Saudi-Arab \| 33 \| \| 24522190 \| GJIC \| Non Syndromic \| Autosomal Dominant \| Canada \|  \| \| 20602914 \| GPSM2 \| Non Syndromic \|  \| Palestinia \| 1 family \| \| 22903915 \| GJB2 \| Non Syndromic \| Autosomal Recessive \| Iran \| 114 families \| \| 29800624 \| OTOG \| Non Syndromic \| Autosomal Recessive \| China \| 1 \| \| 21254920 \| GJA7 \| Non Syndromic \|  \| Turkey, South Africa, United Kingdom, United States, and China \| 341 \| \| 31656313 \| TMEM132E \| Non Syndromic \| Autosomal Recessive \| China \| 1 family \| \| 30235673 \| GJB2, GJB3, SLC26A4, mt-RNR1 \| Non Syndromic \|  \| China \| 1252 \| \| 19732867 \| LOXHD1, MYO3a, PJVK \| Non Syndromic \| Autosomal Recessive \| USA \|  \| \| 25989237 \| GJB2, GJB6 \| Non Syndromic \| Autosomal Recessive \| Syria \| 41 \| \| 32884365 \| MARVELD2 \| Non Syndromic \| Autosomal Recessive \| Pakistan \| 1 \| \| 26226137 \| MYO15A, MYO7A, SLC26A4, TMPRSS3, TMC1, CDH23, ILDR1, OTOF, PCDH15, TMIE, CABP2, ESRRB, GIPC3, LOXHD1, USH1C, CIB2, COL11A2, DFNB59, FAM65B, GPR98, GRXCR1, MARVELD2, MYO6, OTOA, OTOGL, RDX, STRC, TECTA, TPRN, TRIOBP, USH1G \| Non Syndromic \| Autosomal Recessive \| Turkey, Iran, Mexico, Ecuador, and Puerto Rico \| 160 families \| \| 32428919 \| MYO7A \| Non Syndromic \| Autosomal Dominant \| China \| 2 families \| \| 33169910 \| COL11A1 \| Non Syndromic \| Autosomal Dominant \| Germany \| 2 families \| \| 26361564 \| GJB2, SLC26A4, 12SrRNA, and GJB3 \| Non Syndromic \| Autosomal Recessive \| China \| 318 \| \| 25189242 \| GJB2 \| Non Syndromic \|  \| Greek \| 146 \| \| 32279305 \| MYO15A, MYO7A, SLC26A4, GJB2, ADGRV1, BSND, CDH23, GRXCR1, ILDR1, LOXHD1, MYO6, OTOF, PCDH15, POU3F4, PTPRQ, STRC, TECTA, TMC1, TMIE, TMPRSS3, TPRN, TRIOBP, WFS1, \| Non Syndromic \|  \| Egypt \| 48 families \| \| 33316915 \| CDH23 \| Non Syndromic \| Autosomal Recessive \| Saudi-Arab \| 4 \| \| 23226338 \| TRIOBP, TMC1, LOXHD1, TMPRSS3, MYO15A, GIPC3, ILDR1, MYO7A, TECTA \| Non Syndromic \| Autosomal Recessive \| Turkey \| 17 families \| \| 29961571 \| MPZL2 \| Non Syndromic \| Autosomal Recessive \| Duach \| 120 \| \| 25062256 \| ESRRB, TMC1, OTOA, STRC \| Non Syndromic \| Autosomal Recessive \| Turkey \| 103 \| \| 20937258 \| GJB2, GJB6 \| Non Syndromic \| Autosomal Recessive \| Iran \| 2 \| \| 28862181 \| TMC1 \| Non Syndromic \|  \| India \| 47 families \| \| 12746422 \| GJB2 \| Non Syndromic \|  \| India \| 215 \| \| 12833397 \| GJB2 \| Non Syndromic \| Autosomal Recessive \| India \| 45 families \| \| 18570691 \| GJB2 \| Non Syndromic \|  \| India \| 100 \| \| 18941476 \| GJB2 \| Non Syndromic \|  \| India \| 530 \| \| 20086291 \| GJB2, GJB6 \| Non Syndromic \|  \| India \| 303 \| \| 19465004 \| GJB2 \| Non Syndromic \|  \| India \| 210 \| \| 23120683 \| GJB2 \| Non Syndromic \|  \| India \| 288 \| \| 25393658 \| GJB2 \| Non Syndromic \|  \| India \| 19 \| \| 30168495 \| GJB2 \| Non Syndromic \|  \| India \| 316 families \| \| 29921236 \| GJB2 \| Non Syndromic \|  \| India \| 160 families \| \| 29542069 \| GJB2 \| Non Syndromic \|  \| India \| 15 \| \| 33614373 \| GJB2 \| Non Syndromic \|  \| India \| 368 \| \| 34113375 \| GJB2, OTOF, CDH23, MYO15A, OTOG, SLC26A4 \| Non Syndromic \|  \| India \| 52 \| \| 20632892 \| GJC3 \| Non Syndromic \|  \| India \| 123 \| \| 33168789 \| GIPC3 \| Non Syndromic \|  \| India \| 469 \| \| <https://doi.org/10.1016/j.mcp.2015.03.008> \| ADCY1, BDP1, CABP2, ELMOD3, EPS8, GPSM2, GRXCR2, KARS, OTOGL, TBC1D24, TMEM132E, TPRN, TSPEAR, CLIC5, COL4A6, SMPX \| Non Syndromic (AR) \|  \|  \| \| <https://doi.org/10.1016/j.mcp.2015.03.008> \| CEACAM16, P2RX2, OSBPL2, TBC1D24, TNC \| Non Syndromic (AD) \|  \|  \| \| https://doi.org/10.1016/j.ijporl.2008.10.003 \| GJB6, GJB2, GJB3, GJA1, SLC26A4, SLC26A5 \| Hearing loss (AR) \|  \|  \| \| https://doi.org/10.1016/j.bbadis.2008.10.017 \| MYO1F, MYO1C \| Non Syndromic (AR) \|  \|  \| \| https://doi.org/10.1016/S1672-2930(09)50020-7 \| GJB2, SLC26A4 \| Non Syndromic (AR) \|  \|  \| \| https://doi.org/10.1016/j.ijporl.2009.06.009 \| GJB2, GJB6 \| Non Syndromic (AR) \|  \|  \| \| https://doi.org/10.1016/j.bbrc.2010.04.132 \| POU4F3 \| Non Syndromic (AD) \|  \|  \| \| https://doi.org/10.1016/j.ijporl.2010.06.002 \| SLC26A4, FOXI1, KCNJ10 \| Non Syndromic \|  \|  \| \| https://doi.org/10.1590/S1808-86942010000400004 \| GJB2, GJB6 \| Non Syndromic (AR) \|  \|  \| \| https://doi.org/10.1016/j.ijporl.2009.11.014 \| GJB2, GJB6 \| Non Syndromic (AR) \|  \|  \| \| https://doi.org/10.1016/j.ijporl.2010.03.004 \| GJB2, GJB6 \| Non Syndromic \|  \|  \| \| https://doi.org/10.1016/j.ijporl.2011.11.019 \| GJB2 \| Non Syndromic (AR) \|  \|  \| \| https://doi.org/10.1016/j.ijporl.2012.02.056 \| SLC26A4 \| Non Syndromic (AR) \|  \|  \| \| https://doi.org/10.1016/j.ijporl.2012.11.031 \| GJB2, SLC26A4 \| Non Syndromic (AR) \|  \|  \| \| https://doi.org/10.1016/j.gene.2013.06.044 \| OTOGL \| Hearing loss \|  \|  \| \| https://doi.org/10.1016/j.ijporl.2013.07.023 \| SLC26A4 \| Non Syndromic \|  \|  \| \| https://doi.org/10.1016/j.gene.2014.03.033 \| LOXHD1, TMPRSS3, TECTA, MYO15A \| Hearing loss \|  \|  \| \| https://doi.org/10.1016/j.ijporl.2014.01.008 \| GJB2, GJB6, GJB3, SLC26A4 \| Non Syndromic \| China \|  \| \| https://doi.org/10.1016/j.ijporl.2014.09.016 \| GJB2, GJB6 \| Non Syndromic (AR) \|  \|  \| \| https://doi.org/10.1016/j.gene.2015.04.039 \| BCS1L \| Hearing loss (AR) \|  \|  \| \| https://doi.org/10.1016/j.ijporl.2015.10.030 \| GJB2 \| Non Syndromic \|  \|  \| \| https://doi.org/10.1016/j.ijporl.2015.11.018 \| STRC, ACTG1, BSND, CCDC50, CDH23, CEACAM16, CIB2, CLDN14, COCH, COL11A2, CRYM, DFNA5, DFNB59, DFNB31, DIAPH1, ESPN, ESRRB, EYA4, GIPC3, GJB2, GJB3, GJB6, GPSM2, GRHL2, GRXCR1, HGF, ILDR1, KCNQ4, LHFPL5, LOXHD1, LRTOMT, MARVELD2, MIR96, MSRB3, MYH14, MYH9, MYO15A, MYO1A, MYO3A, MYO6, MYO7A, OTOA, OTOF, OTOG, PCDH15, PNPT1, POU4F3, PTPRQ, RDX, SERPINB6, SLC17A8, SLC26A5, TECTA, TJP2, TMC1, TMIE, TMPRSS3, TPRN, TRIOBP, TSPEAR, USH1C, WFS1 \| Non Syndromic (AR) \|  \|  \| \| https://doi.org/10.1016/j.mgene.2016.10.006 \| GJB2 \| Non Syndromic (AR) \|  \|  \| \| https://doi.org/10.1016/j.genrep.2017.10.002 \| OTOA \| Non Syndromic \|  \|  \| \| https://doi.org/10.1016/j.mgene.2017.04.007 \| PTPRQ \| Hearing loss \|  \|  \| \| https://doi.org/10.1016/j.ijporl.2017.09.018 \| ILDR1, MYO6 \| Non Syndromic \|  \|  \| \| https://doi.org/10.1016/j.ijporl.2019.05.036 \| GJB2, GJB6 \| Non Syndromic \|  \|  \| \| https://doi.org/10.1016/j.ijporl.2020.110247 \| STRC \| Non Syndromic \|  \|  \| \| https://doi.org/10.1016/j.ejmg.2019.103796 \| GJB2 \| Non Syndromic (AR) \|  \|  \| \| https://doi.org/10.1016/j.scr.2020.101910 \| GJB2 \| Non Syndromic \|  \|  \| \| https://doi.org/10.1016/j.scr.2020.101795 \| GJB2 \| Hearing loss \|  \|  \| \| https://doi.org/10.1016/j.ajhg.2016.04.015 \| CDC14A \| Profound Hearing loss (AR) \|  \| \| https://doi.org/10.1016/j.ygeno.2014.07.009 \| GJB2, SLC26A4, MT-RNR1 \| Non Syndromic \|  \|  \| \| https://doi.org/10.1016/j.ejmg.2009.06.004 \| EYA4 \| Non Syndromic \|  \|  \| \| DOI: 10.1159/000320154 \| ACTB, BZW, OCM, MACC1, NXPH1, PRPS1L1, RAC1, RPA3 \| Non Syndromic (AR) \|  \|  \| \| https://doi.org/10.1016/j.ijporl.2020.110481 \| MPZL2 \| Non Syndromic \| Morocco \|  \| \| https://doi.org/10.1016/j.ijporl.2020.110286 \| 12S rRNA \| Non Syndromic \| UAE \|  \| \| https://doi.org/10.1016/j.ijporl.2020.110043 \| GJB2 \| Non Syndromic \| Vilnius, Lithuania \| 122 children \| \| https://doi.org/10.1016/j.jgg.2020.07.008 \| PI4KB \| Non Syndromic \| China \| 57 \| \| https://doi.org/10.1016/j.anl.2020.05.008 \| GJB2 \| Non Syndromic \|  \| 24 cases \| \| https://doi.org/10.1016/j.ejmg.2019.103733 \| HARS2 \| Non Syndromic \|  \| 3 \| \| https://doi.org/10.1016/j.ijporl.2019.109607 \| GJB2 \| Non Syndromic \| Iran \|  \| \| https://doi.org/10.1016/j.mito.2020.03.005 \| Mt-RNA \| Non Syndromic \| China \| 887 \| \| https://doi.org/10.1016/j.ijporl.2018.02.037 \| mt-DNA, GJB2 \| Non Syndromic \|  \|  \| \| https://doi.org/10.1016/j.ijporl.2017.03.008 \| MYO15A \| Non Syndromic \| Iran \| 1 \| \| https://doi.org/10.1016/j.devcel.2017.09.026 \| ESRP1 \| Non Syndromic \|  \|  \| \| https://doi.org/10.1016/j.ijporl.2009.01.005 \| TMC1 \| ARNSHL \| Turkey \| 35 \| \| https://doi.org/10.1016/j.hgmx.2016.08.001 \| GJB2 \| NSHL \| Mexico \| 1 family and 100 control \| \| Consanguinity and Hereditary Hearing Loss in Qatar \| LOXHD1, BDP1, GJB2, MYO15A \| NSHL \| Qatar \|  \| \| doi:10.2307/26487724 \| NLRP3 \| Autosomal dominant NSHL \|  \| \| doi:10.1073/pnas.1522512113 \| ROR1 \| Sensorineural \| Miami \|  \| \| https://www.jstor.org/stable/25708727 \| DIAPH3, DIAPH1 \| Autosomal dominant NSHL \| Japan \|  \| \| https://doi.org/10.1016/j.ijporl.2019.02.021 \| MYO7A \| NSHL \|  \| 200 \| \| https://doi.org/10.1016/j.ijporl.2011.11.009 \| GJB2, GJb3, SLC26A4, 12srRNA \| NSHL \| China \| 179 \| |  |
| Risk of bias in studies | 18 | NA |  |
| Results of individual studies | 19 | **Table 1: Studied function classes and associated target genes involved in non-syndromic hearing loss**   \| **Function Classes** \| **Included Functions and GO IDs** \| **Associated Genes** \| **Number of Genes** \| \| --- \| --- \| --- \| --- \| \| Ear Development \| Ear morphogenesis (GO:0042471) \| USH1C, CLIC5, MYO15A, HOXA2, SLC44A4, ATP6V1B1, SIX1, SALL1, MYO3A, NIPBL, ATOH1, FOXI1, NEUROG1, FGF8, HMX2, EYA1, HOXA1, GJB6, HMX3, LHFPL5, COL11A1, PAX2, POU3F4, GATA3, COL2A1, MSX1, SLITRK6, TRIOBP, MYO7A, CHD7, FGFR1, STRC, FGFR2, USH1G, PDZD7, TMC1, LRTOMT, DFNB31, CDH23, GRXCR1, TPRN, NOTCH1, RBPJ, KCNQ1, POU4F3, TGFB3, MCM2, NEUROD1, SLC26A5, USH2A, SLC17A8, SOX2, MAF, DFNA5, PTPN11, OPA1, ROR1, LHX3, PCDH15, ESRRB \| 60 \| \| Inner ear morphogenesis (GO:0042472) \| \| Inner ear receptor cell development (GO:0060119) \| \| Inner ear auditory receptor cell differentiation (GO:0042491) \| \| Inner ear receptor cell stereocilium organization (GO:0060122) \| \| Auditory receptor cell morphogenesis (GO:0002093) \| \| Auditory receptor cell stereocilium organization (GO:0060088) \| \| Cochlea morphogenesis (GO:0090103) \| \| Ear development (GO:0043583) \| \| Outer ear morphogenesis (GO:0042473) \| \| Middle ear morphogenesis (GO:0042474) \| \| Inner ear receptor cell differentiation (GO:0060113) \| \| Cochlea development (GO:0090102) \| \| Vestibulocochlear nerve development (GO:0021562) \| \| Vestibulocochlear nerve formation (GO:0021650) \| \| Auditory receptor cell morphogenesis (GO:0002093) \| \| Auditory receptor cell stereocilium organization (GO:0060088) \| \| Auditory receptor cell development (GO:0060117) \| \| Inner ear development (GO:0048839) \| \| Auditory receptor cell fate commitment (GO:0009912) \| \| Ion Transport \| Ion transport (GO:0006811) \| KCNQ1, CLIC5, SLC22A4, SLC52A3, PANX1, SLC44A4, KCNJ13, ATP6V1B1, SLC19A2, RAF1, SLC12A2, KCNQ4, LRP2, SLC9A1, SLC5A7, SLC26A4, ATP6V1B2, GJA1, ANKH, CACNA1D, TMC1, LOXHD1, SLC26A5, SLC17A8, CLCNKA, CACNA1H, KCNE1, P2RX2, RYR1, LHFPL5, ATP2B2, MT-CO1, OPA1, MT-CO3, KCNJ10, SLC17A9, BSND, CACNA1B, SURF1, CLCNKB, SLC4A11, CATSPER2, SLC12A1, CDH23, ABCC1, SCN9A, TRPV4, GJC1, SLC52A2, ATP1A3, TGFB3, GRHL2, MARVELD2, LAMA3, NF2, ACTB, FLNA, GJB1, GJB2, SNAI2, CLDN9, PEAK1, ACTG1, IL13, EDN3, DIAPH1, CHD7, FAM115A, NEDD4, PROKR2, HGF, TP63, TNC, ILDR2, OTOF, LRP5, NEUROD1, MITF, TOLLIP, FGF8, FGF3, FGFR3, WLS, RAC1, GRM7, ROR1, PCDH15, NDP, GATA3, FGFR1, KIF5A, FGFR2, MPZ, UBC, PMP22, WFS1, CIB2, POLG, TMPRSS3, PROK2, ADCY1, HOMER2, C10orf2, MET, PTPN11, SMAD4, GJB6, PAX2, EDNRB, PDK3, BCAP31, DMXL2, MYO1C \| 113 \| \| Cell junction organization (GO:0034330) \| \| Regulation of ion transmembrane transport (GO:0034765) \| \| Cell junction assembly (GO:0034329) \| \| Cell-cell signalling (GO:0007267) \| \| Gap junction assembly (GO:0016264) \| \| Regulation of potassium ion transmembrane transport (GO:1901379) \| \| Potassium ion transmembrane transport (GO:0071805) \| \| Chemical homeostasis (GO:0048878) \| \| Sodium ion transmembrane transport (GO:0035725) \| \| Regulation of cell junction assembly (GO:1901888) \| \| Ion transmembrane transport (GO:0034220) \| \| Sensory Organ Development \| Sensory organ development (GO:0007423) \| USH1C, KCNQ1, CLIC5, MYO15A, HOXA2, SLC44A4, POU4F3, ATP6V1B1, MFN2, TGFB3, SIX1, SALL1, GRHL2, MCM2, MYO3A, NOTCH1, NIPBL, GJA1, LRP5, NEUROD1, MITF, TMC1, ATOH1, FOXI1, SLC26A5, LRTOMT, USH2A, SIX5, SLC17A8, NEUROG1, FGF8, SOX2, MAF, DFNA5, PTPN11, HMX2, EYA1, HOXA1, NF2, RBPJ, GJB6, HMX3, LHFPL5, DFNB31, OPA1, COL11A1, PAX2, ROR1, LHX3, POU3F4, PCDH15, NDP, GATA3, COL2A1, MSX1, CDH23, GRXCR1, SLITRK6, TRIOBP, MYO7A, TPRN, CHD7, FGFR1, STRC, FGFR2, ESRRB, USH1G, PDZD7 \| 68 \| \| Sensory organ morphogenesis (GO:0090596) \| \| Sensory system development (GO:0048880) \| \| Sensory Signalling \| Sensory perception of sound (GO:0007605) \| USH1C, KCNQ1, CLIC5, MYO15A, SLC52A3, CRYM, COL1A1, WFS1, POU4F3, ATP6V1B1, SIX1, KCNQ4, LRP2, SLC26A4, MYO3A, OTOF, NIPBL, CACNA1D, TMPRSS3, CABP2, TMC1, LOXHD1, SLC26A5, HOMER2, LRTOMT, USH2A, SLC17A8, TSPEAR, MARVELD2, GJC3, CLRN1, KCNE1, DFNA5, EYA1, HOXA1, P2RX2, GJB6, GRM7, LHFPL5, ATP2B2, DFNB31, MYO6, COL11A1, PCDH15, COL11A2, ESPN, GRXCR2, NDP, DCDC2, COL2A1, GJB2, OTOA, PAX3, CCDC50, TECTA, COL4A3, COCH, SNAI2, DIAPH1, CDH23, OTOG, GRXCR1, SLITRK6, TRIOBP, GPR98, MYO7A, DFNB59, TPRN, CHD7, FGFR1, MYO1A, OTOGL, STRC, EYA4, EPS8L2, CEACAM16, MYH14, USH1G, PDZD7, FAM65B, SERPINB6, PROK2, OR51V1, GJB4, OPA1, KCNJ10, PAX2, ROR1, POU3F4, EDNRB, RPGR, SCN9A, GJC1, NEUROG1, ABHD12 \| 95 \| \| Sensory perception (GO:0007600) \| \| Response to auditory stimulus (GO:0010996) \| \| Detection of mechanical stimulus involved in sensory perception of sound (GO:0050910) \|   **Table 2: Identified Highly Interacted Genes and Co-expressed Genes in the PPI Networks of Biological Process Groups, Generated Through STRING**   \| **Sl. No.** \| **Biological Process Groups** \| **Highly interacted Genes & No. of Interactions** \| **Co-expressed Genes** \| \| --- \| --- \| --- \| --- \| \| 1 \| Ear Development \| SOX2 (14) \| EYA1, FGFR1, FGFR2, POU3F4,  SALL1, CHD7 \| \| MYO7A (10) \| CDH23 \| \| PCDH15 (10) \| -NA- \| \| 2 \| Ion Transport \| HGF (10) \| -NA- \| \| UBC (10) \| ACTB \| \| RAC1 (9) \| ACTB \| \| CDH23 (9) \| OTOF, ATP2B2 \| \| 3 \| Sensory Organ Development \| SOX2 (14) \| EYA1, FGFR1, FGFR2, POU3F4,  SALL1, CHD7 \| \| MYO7A (10) \| CDH23 \| \| PCDH15 (10) \| -NA- \| \| 4 \| Sensory Signalling \| CDH23 (16) \| OTOF, MYO7A, ATP2B2 \| \| MYO7A (14) \| CDH23 \|   **Table 3: Identified most deleterious pathogenic nsSNPs in the target genes of NSHL**   \| **Sl. No.** \| **Gene Names** \| **UniProt IDs** \| **Most Deleterious nsSNPs (Deleterious in SIFT, Predict SNP1, PredictSNP2)** \| **Variants and Positions** \| \| --- \| --- \| --- \| --- \| --- \| \| 1 \| OTOF \| Q9HC10 \| rs80356586 \| Ile515Thr \| \| rs80356596 \| Leu1011Pro \| \| rs80356606 \| Pro1987Arg \| \| 2 \| FGFR1 \| P11362 \| rs121909642 \| Pro722Ser \| \| rs267606805 \| Pro722His \| \| 3 \| FGFR2 \| P21802 \| rs121918506 \| Glu565Ala \| \| rs121918509 \| Ala628Thr, Ala629Thr \|     **Fig. 1:** Flowchat of systematic review followed by PRISMA guidelines  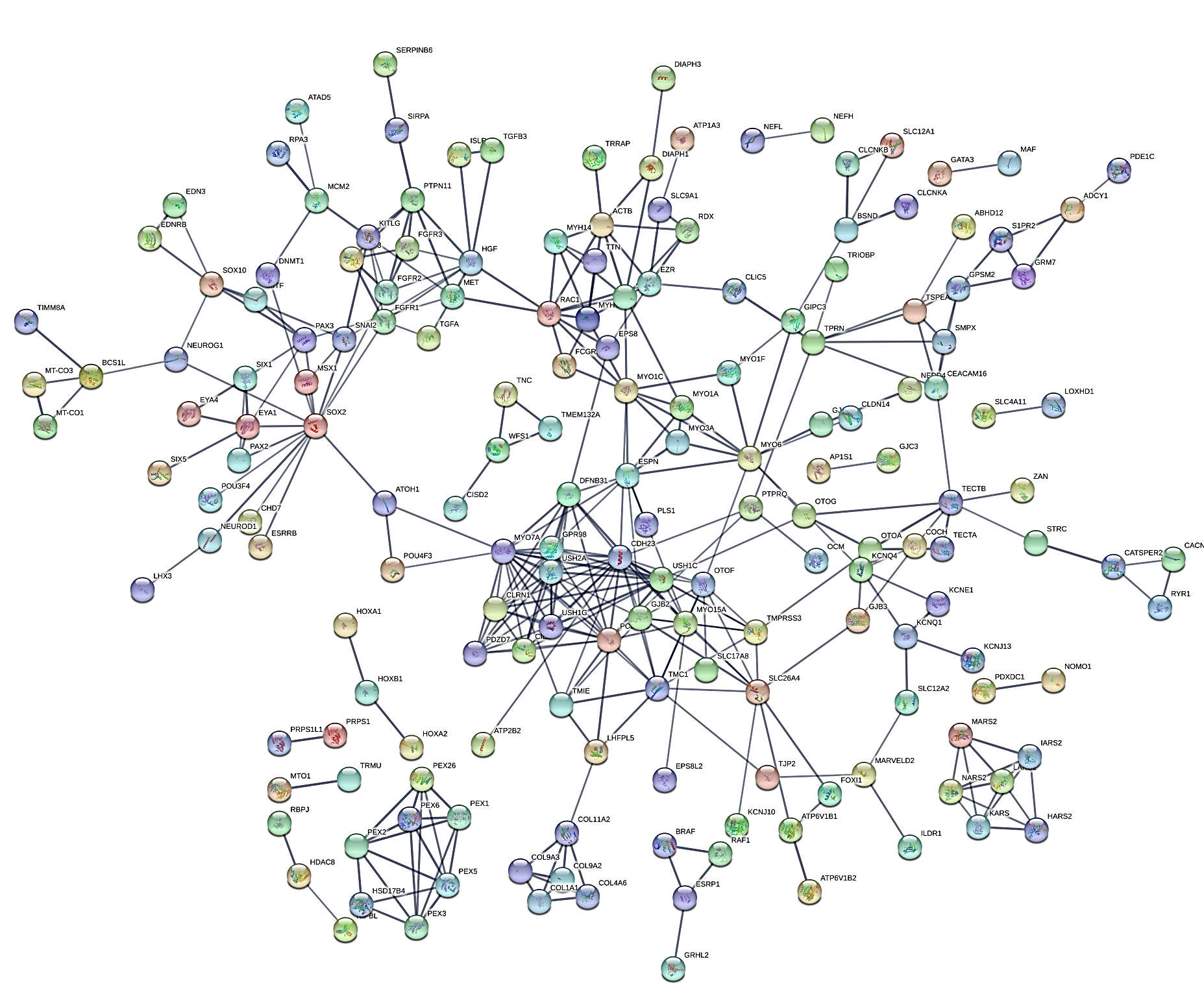  **Fig. 2:** Protein-Protein interaction network between all the reviewed target genes (382) through STRING  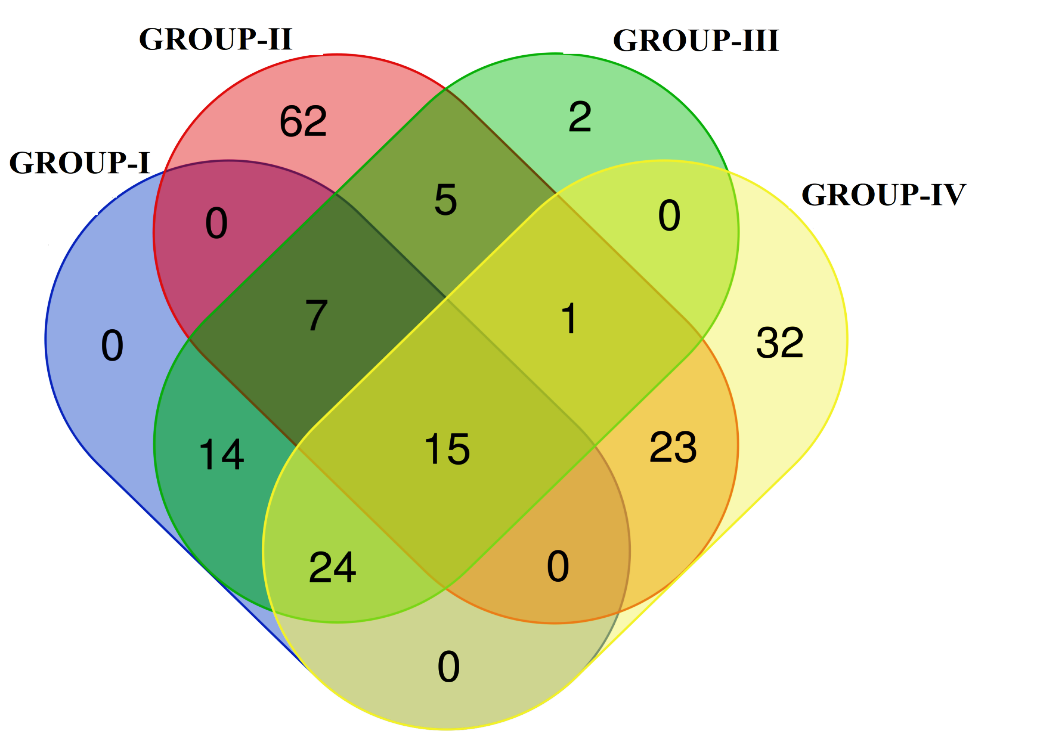  **Fig. 3:** Number of unique and commonly identified genes in four different groups represented through venn diagram  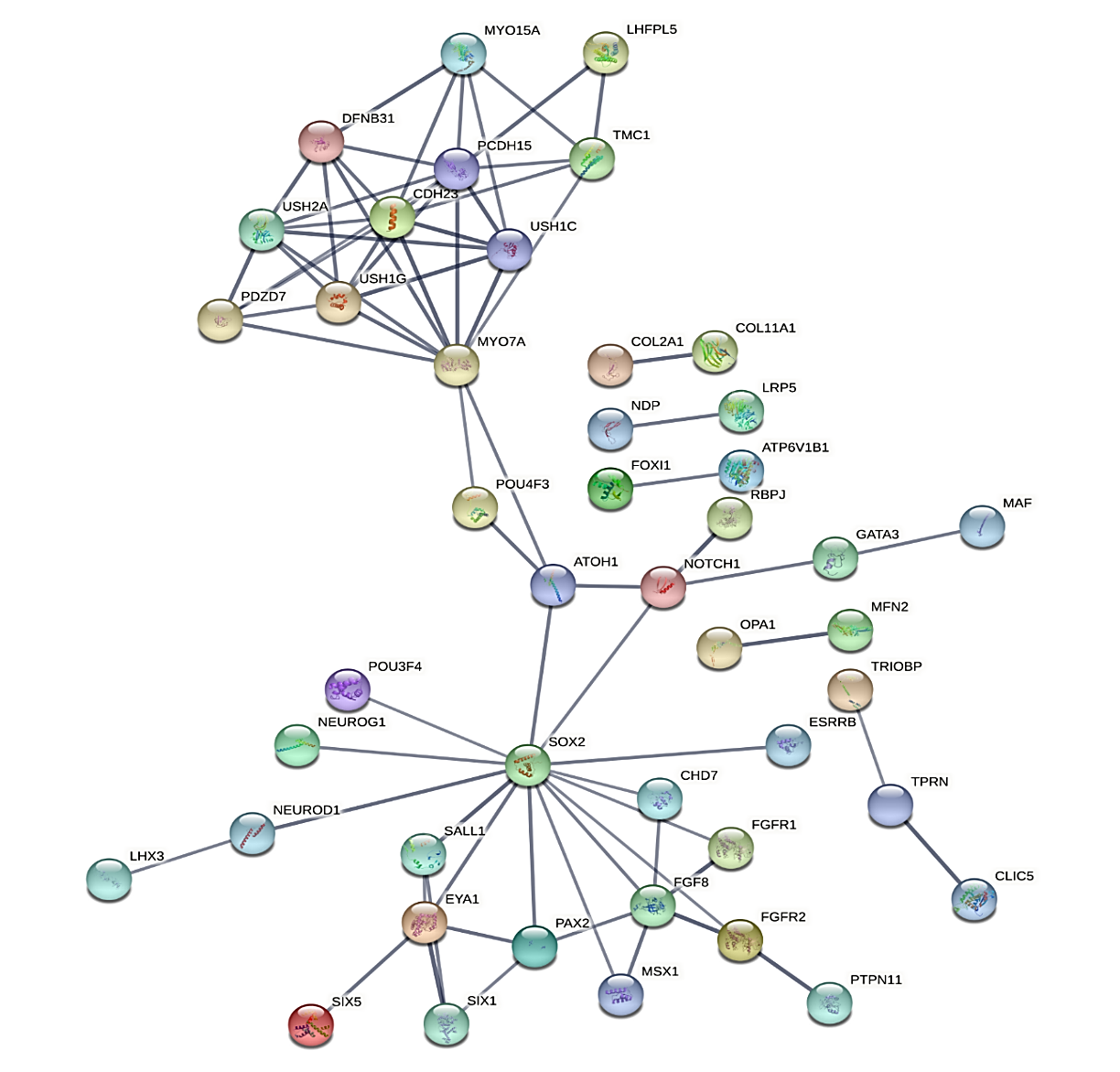  **Fig. 4:** Protein functional network built between ear development group of genes (Group I)  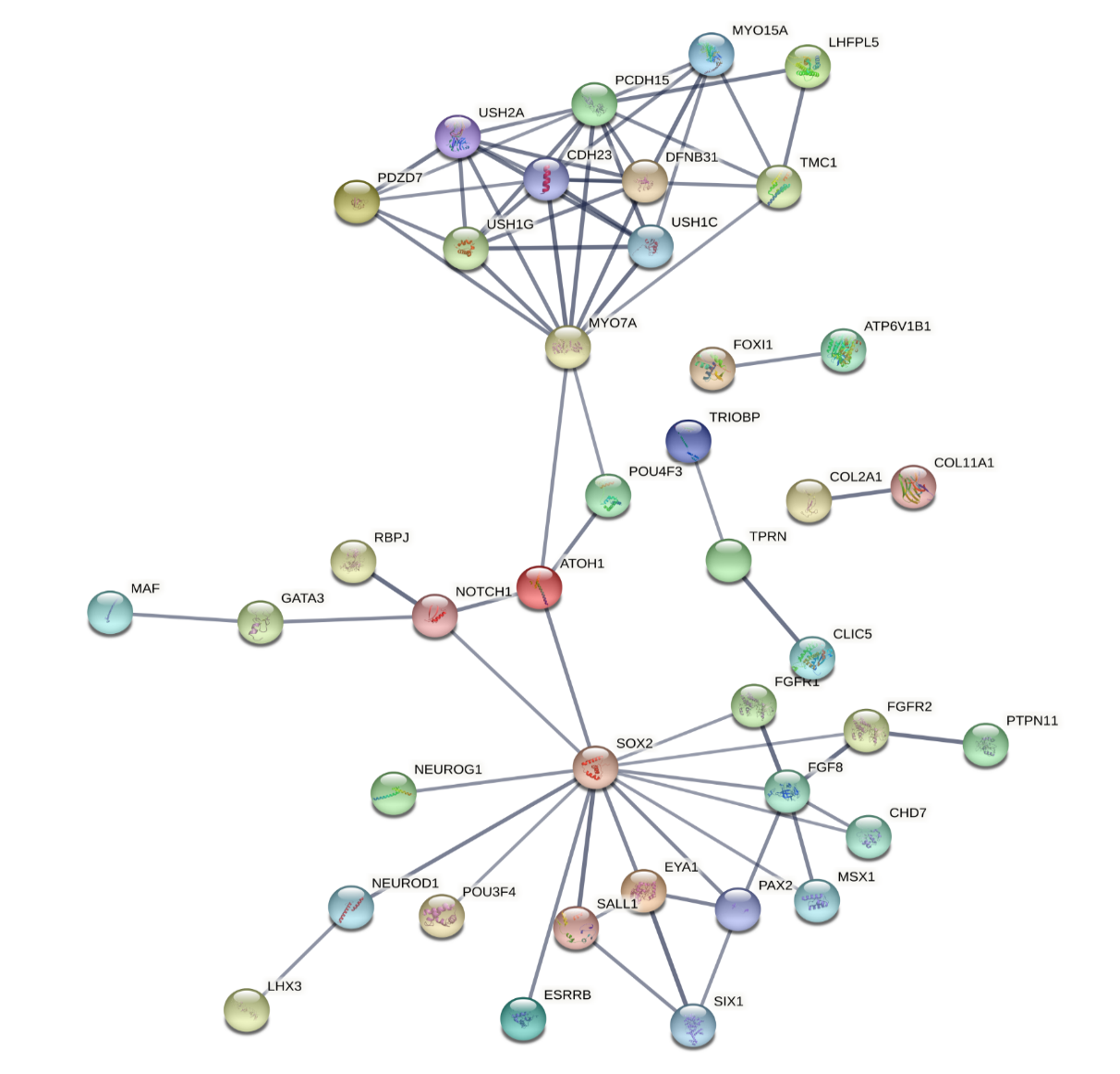  **Fig. 5:** Generated protein interaction network between ion transport group of genes (Group II)  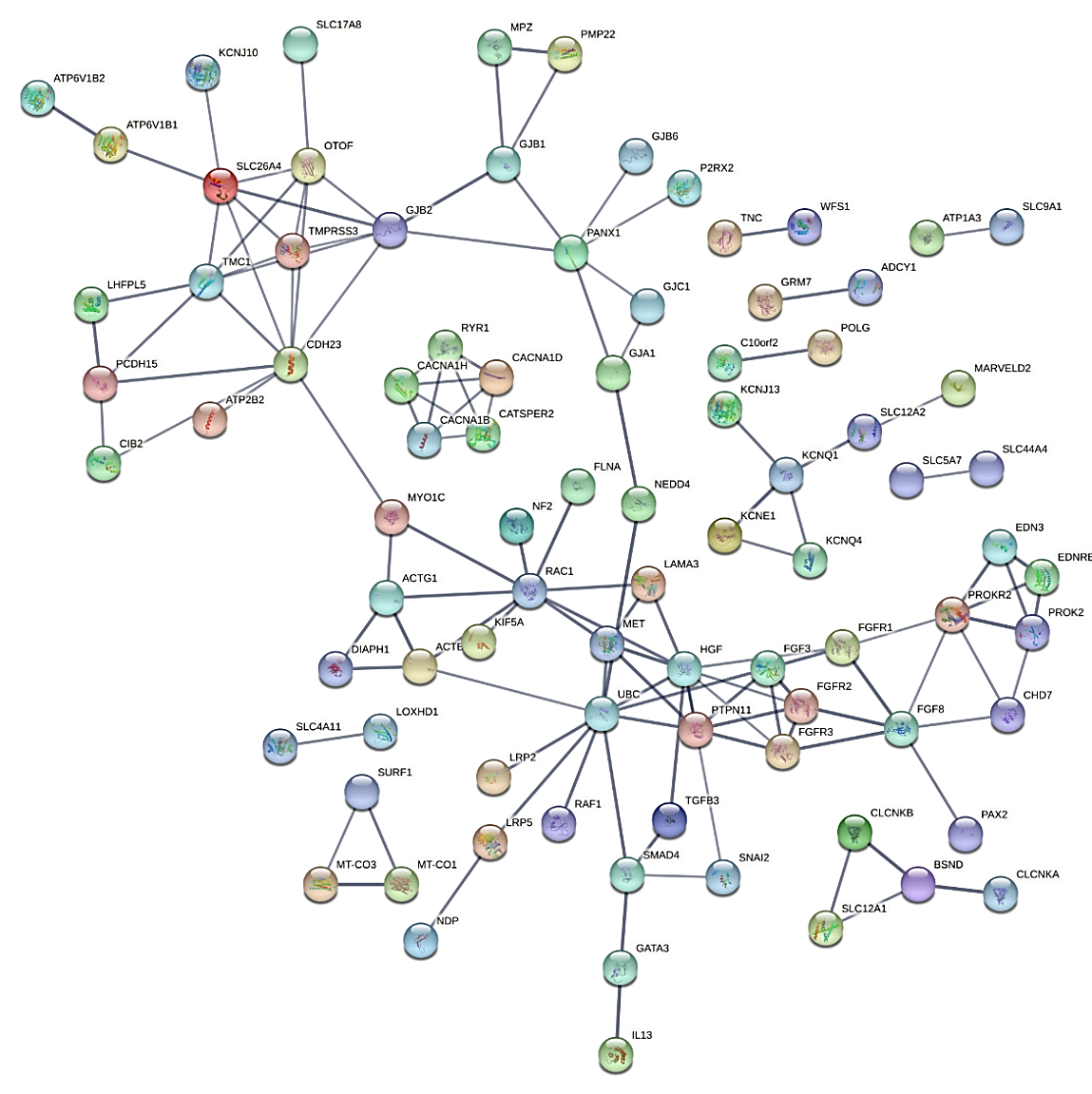  **Fig. 6:** Network represents the strong functional association between sensory organ development group of genes (Group III)  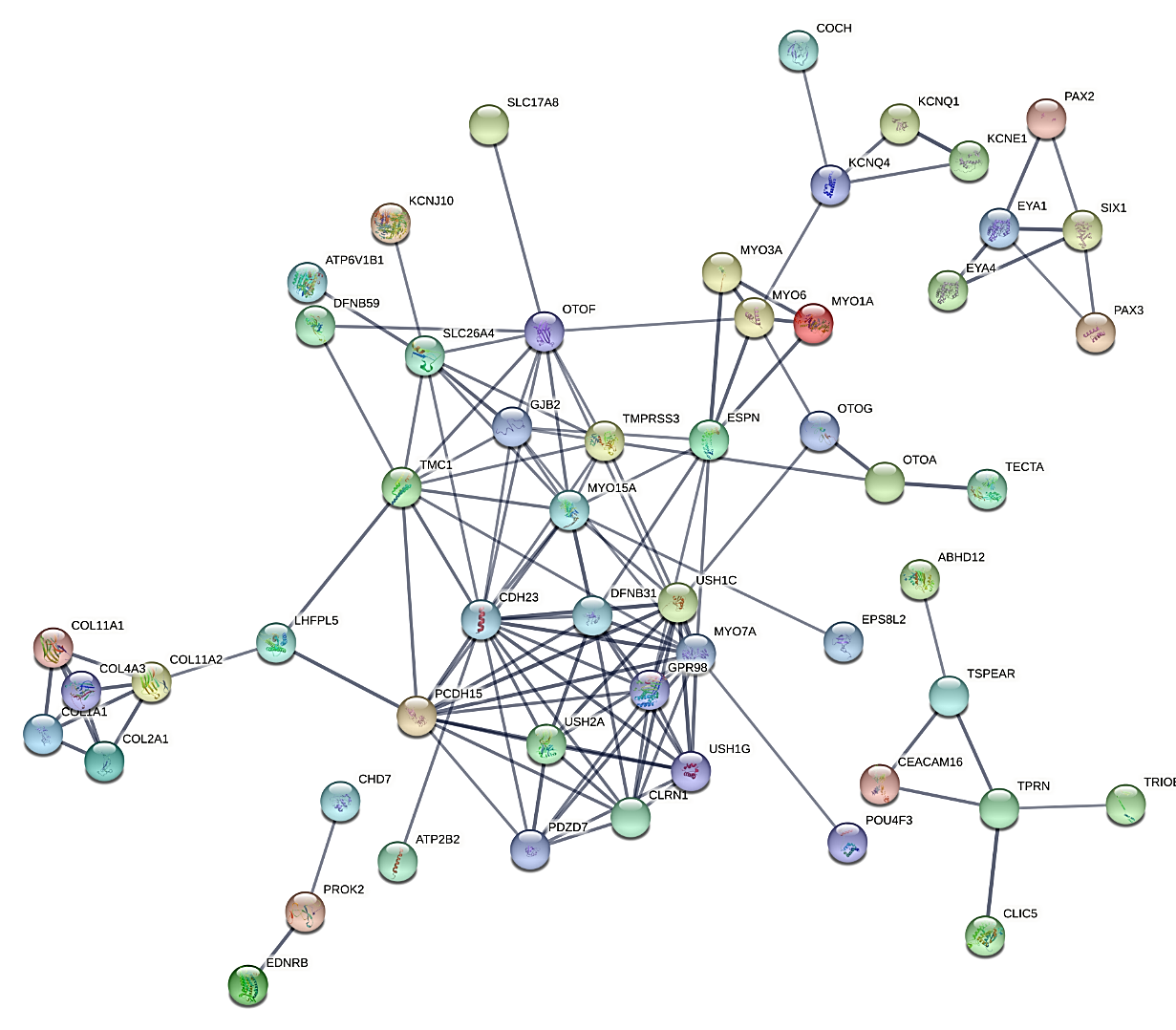  **Fig. 7:** PPI enrichment network of sensory signalling group of genes (Group IV) | Results (Table 1: Line Number 185, 192) Table 2 (Line Number 214) Table 3 (Line Number 241)  Fig. 1 (Line Number 156)  Fig. 2 ( Line Number 165)  Fig. 3 ( Line Number 192)  Fig. 4 ( Line Number 197)  Fig. 5 ( Line Number 200)  Fig. 6 ( Line Number 2013)  Fig. 7 ( Line Number 205) |
| Results of syntheses | 20a | NA |  |
|  | 20b | NA |  |
|  | 20c | Used different methodologies  Different study population |  |
|  | 20d | NA |  |
| Reporting biases | 21 | NA |  |
| Certainty of evidence | 22 | NA |  |
| **DISCUSSION** | | |  |
| Discussion | 23a | The pathogenic nsSNPs for all the highly interacted and co-expressed genes have been collected from dbSNP and analysed in SIFT, PredictSNP1 and PredictSNP2. Among the analysed nsSNPs, only some nsSNPs rs80356586, rs80356596, rs80356606 in OTOF, rs121909642 in FGFR1, and rs121918506, rs121918509 in FGFR2 have been identified as deleterious in all the prediction algorithms. So these variants can be called as most deleterious SNPs in the respective genes, which might leads to NSHL. The gene OTOF is responsible for the composition of ribbon synaptic vesicles in cochlear inner hair cells, and the mutations in OTOF are responsible for 2-3% of NSHL [9,41]. The association of OTOF in NSHL has been experimented in immortal lymphoblastoid cell lines, inner hair cell (IHC) and human embryonic kidney cells (HEK) [42, 43]. In this study, the most deleterious nsSNPs rs80356586 (Ile515Thr) [44], rs80356596 (Leu1011Pro) [45,46], rs80356606 (Pro1987Arg) [47] found in OTOF gene were reported in UniProt and ClinVar datasets for NSHL cases. Among these three variants, two have been identified in the Turkish population (Ile515Thr, Leu1011Pro) and one in northern Lebanon populations (Pro1987Arg).  The involvement of FGFR1 has been reported in the development of the auditory sensory epithelium in vitro studies on mice [48]. FGFR1 and FGFR2 have also been used in the reference gene panel, which has been used in the genomic diagnosis of NSHL cases in the Spain population earlier [42]. No reports have described either the role of FGFR1 and FGFR2, or on presently predicted nsSNPs rs121909642 (Pro722Ser) of FGFR1 and rs121918506 (Glu565Ala), rs121918509 (Ala628Thr, Ala629Thr) of FGFR2 in the NSHL in humans.  However, the variants of FGFR1 and FGFR2 have been found in hypogonadotropic hypogonadism 2 with anosmia (rs121909642) [49], pfeiffer syndrome [50], craniosynostosis syndrome [51] (rs121918506), and in LADD syndrome (rs121918509) [52,53] respectively. No disease was found for the variant rs267606805 (P722H) in the ClinVar database. The unreported variants in FGFR1 and FGFR2 in NSHL cases indicates the uniqueness of the predicted results. | Discussion (Line Number 312-331) |
|  | 23b | NA |  |
|  | 23c | - We have reviewed the articles published between 2009-2020 - Only original research articles have been included in the study - Only the reported target genes have been retrieved from the articles | Materials & Method (Line Number 106-109) |
|  | 23d | The identified most deleterious variants of the respective target genes may be clinically validated through molecular approaches in future research, which can be used in the genetic diagnosis of NSHL patients in future diagnosis. |  |
| **OTHER INFORMATION** | | |  |
| Registration and protocol | 24a | The review was not registered. |  |
|  | 24b | A protocol was not prepared. |  |
|  | 24c | NA |  |
| Support | 25 | Indian Council of Medical Research (ICMR), New Delhi, India |  |
| Competing interests | 26 | NA |  |
| Availability of data, code and other materials | 27 | The collected target genes are available in the research articles collected from online databases including (<https://pubmed.ncbi.nlm.nih.gov/>), ScienceDirect (https://www.sciencedirect.com/), Cochrane Library (https://www.cochranelibrary.com/) and JSTOR (https://www.jstor.org/) | Materials & Method (Line Number 90-99) |

*From:*  Page MJ, McKenzie JE, Bossuyt PM, Boutron I, Hoffmann TC, Mulrow CD, et al. The PRISMA 2020 statement: an updated guideline for reporting systematic reviews. BMJ 2021;372:n71. doi: 10.1136/bmj.n71

For more information, visit: <http://www.prisma-statement.org/>
